# Identification of *TBX15* as an adipose master *trans* regulator of abdominal obesity genes

**DOI:** 10.1186/s13073-021-00939-2

**Published:** 2021-08-02

**Authors:** David Z. Pan, Zong Miao, Caroline Comenho, Sandhya Rajkumar, Amogha Koka, Seung Hyuk T. Lee, Marcus Alvarez, Dorota Kaminska, Arthur Ko, Janet S. Sinsheimer, Karen L. Mohlke, Nicholas Mancuso, Linda Liliana Muñoz-Hernandez, Miguel Herrera-Hernandez, Maria Teresa Tusié-Luna, Carlos Aguilar-Salinas, Kirsi H. Pietiläinen, Jussi Pihlajamäki, Markku Laakso, Kristina M. Garske, Päivi Pajukanta

**Affiliations:** 1 Department of Human Genetics, David Geffen School of Medicine at UCLA, Los Angeles, USA; 2 Bioinformatics Interdepartmental Program, UCLA, Los Angeles, USA; 3 Computational and Systems Biology Interdepartmental Program, UCLA, Los Angeles, USA; 4 Institute of Public Health and Clinical Nutrition, University of Eastern Finland, Kuopio, Finland; 5 Department of Medicine, David Geffen School of Medicine at UCLA, Los Angeles, USA; 6 Department of Computational Medicine, David Geffen School of Medicine at UCLA, Los Angeles, USA; 7 Department of Genetics, University of North Carolina at Chapel Hill, Chapel Hill, North Carolina USA; 8 Center for Genetic Epidemiology, Department of Preventative Medicine, Keck School of Medicine, University of Southern California, Los Angeles, USA; 9 Tecnologico de Monterrey, Escuela de Medicina y Ciencias de la Salud, Ave. Morones Prieto 3000, Monterrey, N.L. México 64710; 10 Unidad de Investigación de Enfermedades Metabólicas, Instituto Nacional de Ciencias Médicas y Nutrición Salvador Zubirán, Mexico City, Mexico; 11 Departamento de Endocrinología y Metabolismo del Instituto Nacional de Ciencias Médicas y Nutrición Salvador Zubirán, Mexico City, Mexico; 12 Departamento de Cirugía, Instituto Nacional de Ciencias Médicas y Nutrición, Mexico City, Mexico; 13 Unidad de Biología Molecular y Medicina Genómica, Instituto de Investigaciones Biomédicas UNAM/ Instituto Nacional de Ciencias Médicas y Nutrición Salvador Zubirán, Mexico City, Mexico; 14 Obesity Research Unit, Research Program for Clinical and Molecular Metabolism, Faculty of Medicine, University of Helsinki, Helsinki, Finland; 15 Obesity Center, Endocrinology, Abdominal Center, Helsinki University Central Hospital and University of Helsinki, Helsinki, Finland; 16 Department of Medicine, Endocrinology and Clinical Nutrition, Kuopio University Hospital, Kuopio, Finland; 17 Department of Medicine, University of Eastern Finland and Kuopio University Hospital, Kuopio, Finland; 18 Institute for Precision Health at UCLA, Los Angeles, USA

**Keywords:** Transcriptional regulation of abdominal obesity, Master transcription factor, *Trans* regulation of genes, Waist-hip ratio adjusted for body mass index (WHRadjBMI), Type 2 diabetes (T2D), Polygenic risk score (PRS)

## Abstract

**Background:**

Obesity predisposes individuals to multiple cardiometabolic disorders, including type 2 diabetes (T2D). As body mass index (BMI) cannot reliably differentiate fat from lean mass, the metabolically detrimental abdominal obesity has been estimated using waist-hip ratio (WHR). Waist-hip ratio adjusted for body mass index (WHRadjBMI) in turn is a well-established sex-specific marker for abdominal fat and adiposity, and a predictor of adverse metabolic outcomes, such as T2D. However, the underlying genes and regulatory mechanisms orchestrating the sex differences in obesity and body fat distribution in humans are not well understood.

**Methods:**

We searched for genetic master regulators of WHRadjBMI by employing integrative genomics approaches on human subcutaneous adipose RNA sequencing (RNA-seq) data (*n* ~ 1400) and WHRadjBMI GWAS data (*n* ~ 700,000) from the WHRadjBMI GWAS cohorts and the UK Biobank (UKB), using co-expression network, transcriptome-wide association study (TWAS), and polygenic risk score (PRS) approaches. Finally, we functionally verified our genomic results using gene knockdown experiments in a human primary cell type that is critical for adipose tissue function.

**Results:**

Here, we identified an adipose gene co-expression network that contains 35 obesity GWAS genes and explains a significant amount of polygenic risk for abdominal obesity and T2D in the UKB (*n* = 392,551) in a sex-dependent way. We showed that this network is preserved in the adipose tissue data from the Finnish Kuopio Obesity Study and Mexican Obesity Study. The network is controlled by a novel adipose master transcription factor (TF), *TBX15*, a WHRadjBMI GWAS gene that regulates the network in *trans*. Knockdown of *TBX15* in human primary preadipocytes resulted in changes in expression of 130 network genes, including the key adipose TFs, *PPARG* and *KLF15*, which were significantly impacted (FDR < 0.05), thus functionally verifying the *trans* regulatory effect of *TBX15* on the WHRadjBMI co-expression network.

**Conclusions:**

Our study discovers a novel key function for the *TBX15* TF in *trans* regulating an adipose co-expression network of 347 adipose, mitochondrial, and metabolically important genes, including *PPARG*, *KLF15*, *PPARA*, *ADIPOQ*, and 35 obesity GWAS genes. Thus, based on our converging genomic, transcriptional, and functional evidence, we interpret the role of *TBX15* to be a main transcriptional regulator in the adipose tissue and discover its importance in human abdominal obesity.

**Supplementary Information:**

The online version contains supplementary material available at 10.1186/s13073-021-00939-2.

## Background

Obesity predisposes individuals to multiple cardiometabolic disorders, including type 2 diabetes (T2D) [1, 2]. Furthermore, as the world faces one of the worst infectious-disease outbreaks in a century, new data are emerging showing that obesity and male sex are key risk factors for severe forms of COVID-19 infection in individuals less than 60 years of age [3, 4]. However, the underlying genes and regulatory mechanisms orchestrating the sex differences in obesity and body fat distribution are not well understood.

Obesity is clinically diagnosed by a body mass index (BMI) greater than 30 kg/m^2^, while severe obesity is defined as BMI greater than 40 kg/m^2^. However, as BMI cannot reliably differentiate fat from lean mass, the metabolically detrimental abdominal obesity has been more accurately estimated using waist-hip ratio (WHR), which even after adjusting for BMI (WHRadjBMI) is still highly heritable (heritability ~ 0.22–0.61 )[5–8]. WHRadjBMI is a well-established surrogate for abdominal adiposity and body fat distribution, and it has also been correlated with direct imaging assessments of abdominal fat in observational studies [9–11]. It is also recognized as a strong predictor of T2D [12].

Previous studies have demonstrated that WHRadjBMI is a sexually dimorphic trait, reflecting the physiological differences in body fat and muscle mass, with males in general exhibiting more muscle mass and females more fat mass when matched for BMI and age [13, 14]. Furthermore, WHRadjBMI shows large differences in the narrow sense heritability between males (~ 20%) and females (~ 50%) [8, 15]; yet, the biological mechanisms underlying abdominal adiposity and its sex-specific characteristics have remained largely elusive. Previous genome-wide association studies (GWAS) have shown that WHRadjBMI GWAS genes are enriched for adipose-expressed genes with known adipose tissue functions, whereas BMI GWAS genes are enriched for genes expressed primarily in the brain [16]. To advance the discovery of unknown genetic and molecular mechanisms regulating abdominal adiposity and the sex-specific distribution of body fat, we searched for genetic master regulators of WHRadjBMI by employing integrative genomics approaches on human subcutaneous adipose RNA sequencing (RNA-seq) data (*n* ~ 1400) and WHRadjBMI GWAS, transcriptome-wide association studies (TWAS), and polygenic risk score (PRS) data from the WHRadjBMI GWAS cohorts and the UK Biobank (UKB) (*n* ~ 700,000). Finally, we verified our genomic results using functional studies in a human primary cell type that is crucial for adipose tissue function.

One possible regulatory mechanism of gene expression is transcription factor (TF) binding to the promoters of multiple genes across many chromosomes, which causes them to be co-regulated and co-expressed [17–19]. We hypothesized that adipose co-expression networks can be used to identify novel TFs that *trans* regulate multiple co-expressed target genes important for WHRadjBMI.

We provide novel genomic evidence, verified by our functional studies in human primary preadipocytes, for the causal role of *TBX15* in controlling accumulation of abdominal fat and adiposity. Our study discovers a new key function for the TBX15 TF in *trans* regulating an adipose co-expression network of 347 adipose, mitochondrial, and metabolically important genes, including *PPARG*, *KLF15*, *PPARA*, *ADIPOQ*, and 35 obesity GWAS genes.

## Methods

### Study cohorts

#### METabolic Syndrome In Men (METSIM) cohort used for discovery of WHRadjBMI co-expression network

The participants in the METSIM cohort (*n* = 10,197) are Finnish males recruited at the University of Eastern Finland and Kuopio University Hospital, Kuopio, Finland, as described previously [20–22]. The study was approved by the local ethics committee and all participants gave written informed consent. The median age of the METSIM participants is 57 years (range 45–74 years). The METSIM participants were genotyped using the OmniExpress (Illumina) genotyping array and phased and imputed using SHAPEIT2 v2.17 [23] and IMPUTE2 v2.3.2 [24], respectively. A random subset of the METSIM men underwent an abdominal subcutaneous adipose needle biopsy, with 335 unrelated individuals (IBD < 0.2 using a genetic relationship matrix calculated in PLINK v1.9 [25]) analyzed here using RNA-seq [22, 26].

#### UK Biobank (UKB) cohort used for construction of PRS

The UKB is a large cohort (*n* = 502,617) consisting of data from individuals collected across the UK starting in 2006 [27, 28]. To avoid hidden confounders from ancestry and relatedness, we used the subset of these individuals who are unrelated and of European ancestry (*n* = 392,551). The genotyping was performed using one of two arrays for over 800,000 different variants [28, 29]. The genotypes were then imputed using the Haplotype Reference Consortium (HRC) as well as UK 10 K panel and the 1000 Genomes panel [28, 29]. The genotypes were filtered for variants with MAF < 1% and violation of Hardy-Weinberg Equilibrium (*p* < 1 × 10^−6^) before using them for construction of the polygenic risk scores (PRSs) for WHRadjBMI.

#### Kuopio OBesity Study (KOBS) cohort used for testing of *TBX15* in extreme obesity

The participants in the longitudinal Kuopio OBesity Study (KOBS) cohort (*n* = 168) consist of Finnish obese individuals undergoing bariatric surgery and participating in a 1-year follow-up, recruited at the University of Eastern Finland and Kuopio University Hospital, Kuopio, Finland, as described previously [30–33]. The study was approved by the local ethics committee and all participants gave written informed consent. All participants underwent a pre-screening for a detailed medical history, and the inclusion criterion was a pre-surgery BMI of ≥ 40 kg/m^2^ or 35 kg/m^2^ with a significant comorbidity, such as type 2 diabetes (T2D). A total of 168 individuals with subcutaneous adipose RNA-seq data at the time of bariatric surgery and one year after the surgery were analyzed in our study [33]. Refined phenotypic measurements and clinical characteristics were also measured at both time points [30–33].

#### Mexican Obesity Study (MOSS) cohort used for independent replication of WHRadjBMI co-expression network preservation

The participants in the on-going longitudinal Mexican Obesity Study (MOSS) cohort are recruited at the Instituto Nacional de Ciencias Medicas y Nutricion (INCMN), Mexico City, as described in detail in Miao et al. [34]. Briefly, the MOSS cohort consists of Mexican obese individuals undergoing bariatric surgery and participating in a 1-year follow-up. A total of 43 individuals with subcutaneous adipose RNA-seq data on both time points [34] were analyzed in our study. The study was approved by the local ethics committee, and all participants gave written informed consent. All participants underwent a pre-screening for a detailed medical history, and the inclusion criteria were a pre-surgery BMI of ≥ 33 kg/m^2^, no weight loss or gain after an intensive diabetes control program, and good pancreatic reserve for individuals with T2D. Individuals were excluded if they had a medical condition that limited their life expectancy to less than 5 years. The biopsy samples were taken from subcutaneous adipose tissue at the time of bariatric surgery and 1 year after the surgery. Refined phenotypic measurements and clinical characteristics were also measured at both time points. To control for admixed ancestry of Mexican individuals, we called variants from the RNA-seq data following the GATK pipeline [35]. We used the recommended parameters of -window 35, -cluster 3, and filtering FS > 30 and QD < 2 and only included variants with MAF > 5% and an average read depth ≥ 30. To ensure the quality of our genotypes, we combined the MOSS and 1000 Genomes Project genotype data [36] and performed principal component analysis (PCA) and observed that the MOSS individuals clustered well with the individuals of Amerindian descent. We used these variants called from RNA-seq data to calculate the genotype PCs for the correction of ancestry.

### Alignment of RNA-seq data

We performed alignment of subcutaneous adipose RNA-seq data (*n* = 335) from the METSIM cohort [26] using STAR v2.5.2 [37] with GENCODE v19 annotation of the genome and hg19 version of the human genome, as we described earlier with minor changes [22, 38]. Briefly, a 2-pass alignment was performed on 75 base-pair (bp) reads with only uniquely mapped reads counted for gene expression. We discovered that the expression of many genes and technical factors are correlated with the percentage of mitochondrial reads. To avoid the influence of the mitochondrial read number on the data, we excluded the mitochondrial reads from the RNA-seq data when calculating the FPKMs and technical factors. We used FastQC to verify the RNA-seq quality, based on metrics, such as GC content, duplication levels, and sequence quality scores, as well as Picard Tools v2.9.0 to obtain the technical factors from the standard RNA-seq metrics (option CollectRNAseqMetrics), including the median 5′ to 3′ bias, percentage of intronic reads, and median coverage from the aligned reads.

### Weighted Gene Co-expression Analysis

To find co-expression networks in the METSIM adipose RNA-seq cohort, we performed weighted gene co-expression analysis (WGCNA) v1.68 [39] on FPKMs from the subcutaneous adipose RNA-seq data (*n* = 335) from the METSIM cohort [26]. To prevent the influence of technical factors from sequencing and RNA-seq alignment, we included 14 technical factors that were determined by STAR v2.5.2 [37] and Picard Tools v2.9.0. The FPKMs were filtered for genes expressed (FPKM> 0) in at least 90% of individuals and inverse normal transformed after correcting for technical factors to avoid spurious associations and outlier effects (see above). Phenotypes used for associations with co-expression networks in WGCNA v 1.68 [39] were inverse normal transformed after correcting for age, age^2^. The fasting serum insulin levels were corrected for T2D status as well as age and age^2^ and then inverse normal transformed. To ensure scale-free network topography, we used a power of 10 for the power function to determine co-expression network membership. All other parameters in WGCNA v 1.68 [39] were kept at their default values.

Selection of parameters for WGCNA [39] is based on the assumption that the degree of connection of genes in a gene expression network should follow a power law, thus approximating a scale-free network, as described in the original WGCNA paper [39]. Briefly, this requires the testing of different values for a soft-thresholding power to determine at which power the gene expression network starts to approximate a scale-free network. The optimal value is best determined by the scale-free topography model fit parameter, as determined by WGCNA [39], with the ideal soft-thresholding power being the lowest power at which the gene expression networks approximate a scale-free topography because this retains the highest amount of connectivity between genes (Additional file 1: Fig. S1).

### Co-expression network preservation

Using WGCNA v1.68 [39, 40], we confirmed the preservation of the co-expression networks from the METSIM subcutaneous adipose RNA-seq [26] (*n* = 335) in the subcutaneous adipose (v8, *n* = 581), visceral adipose (v7, *n* = 277), and skeletal muscle (v8, *n* = 298) RNA-seq data from the independent GTEx cohort [41, 42] and the Mexican MOSS cohort [34]. We further subdivided the GTEx [41] cohort to males (*n* = 387, *n* = 149, *n* = 153, subcutaneous adipose, visceral adipose, skeletal muscle, respectively) and females (*n* = 194, *n* = 84, *n* = 145, subcutaneous adipose, visceral adipose, skeletal muscle, respectively) and then for the subcutaneous adipose data, lean (BMI < 25, *n*_Males_ = 102, *n*_Females_ = 78) and obese (BMI > 30, *n*_Males_ = 119, *n*_Females_ = 41) individuals of each sex. We did not subdivide the visceral and skeletal muscle data into lean and obese categories by BMI as the samples sizes would have been below the recommended minimum threshold for network preservation (*n* = 20). The analysis of the MOSS cohort [34] was also not done in a sex-specific manner due to the small sample size. When sample sizes are above the recommended minimum threshold (*n* = 20), the *Z*_Summary_ score value should not be sensitive to the sample size, and so the relative difference in the number of males and females or lean and obese individuals was not a concern. We calculated FPKMs from the RNA-seq data and technical factors from STAR v2.5.2 [37] and Picard Tools v2.9.0, as described above. We corrected the expression data for technical factors as well as age, age^2^, sex, race, RIN, and then inverse normal transformed the data. The GTEx v8 cohort data [41] was also corrected for sequencing platform, sequencing protocol (PCR-based or PCR-free), time from death to RNA collection, and 5 genotype principal components (PCs) to correct for ancestry. The MOSS cohort [34] was corrected for an additional three genotype PCs using PLINK v1.9 [25] to account for ancestry. Default parameters in WGCNA v1.68 [39] were used for the co-expression network preservation analysis. Accordingly, a preservation 10 > *Z*_Summary_ > 2 was considered as weakly to moderately preserved and a *Z*_Summary_ > 10 as strongly preserved [39, 40]. The calculation of the *Z*_Summary_ score requires a reference cohort and set of networks and an independent test cohort. The same networks from the reference cohort are assigned to genes in the test cohort data. Then metrics, such as the similarity of the eigengenes, connectivity between genes and to the eigengene, and the ability to separate a specific network from other networks are aggregated into a *Z*_Summary_ score, indicating the similarity of a specific network in the test cohort data and the reference cohort data. As the distribution of *Z*_Summary_ scores is not known a priori, the significance of the *Z*_Summary_ score is determined via permutations of the labels of the networks in the test cohort data and re-calculating *Z*_Summary_ score.

### Single-nucleus RNA-seq (snRNA-seq) of human subcutaneous adipose tissue

We performed the snRNA-seq analysis of frozen adipose tissue biopsies obtained from 15 individuals (6 males and 9 females with a mean age = 32.70 ± 7.12 and mean BMI = 31.45 ± 5.42). These 15 individuals underwent subcutaneous adipose biopsies as part of the Finnish Twin study (7 individuals) [43] and CRYO study (8 individuals) [33, 44] at the Helsinki University Central Hospital, Finland. The Finnish Twin and CRYO studies were approved by the local ethics committee and all participants gave written informed consent. To identify cell types and their gene expression profile, we first isolated nuclei from frozen subcutaneous adipose tissue to input them into the 10X Chromium platform [45]. To isolate nuclei from frozen tissue, the tissue was minced over dry ice and transferred into ice-cold lysis buffer consisting of 0.1% IGEPAL, 10 mM Tris-HCl, 10 mM NaCl, and 3 mM MgCl_2_. After a 10-min incubation period, the lysate was gently homogenized using a dounce homogenizer and filtered through a 70-μm MACS smart strainer (Miltenyi Biotec #130-098-462) to remove debris. Nuclei were centrifuged at 500*g* for 5 min at 4 °C and washed in 1 ml of resuspension buffer (RSB) consisting of 1× PBS, 1.0% BSA, and 0.2 U/μl RNase inhibitor. We further filtered nuclei using a 40-μm Flowmi cell strainer (Sigma Aldrich # BAH136800040) and centrifuged at 500*g* for 5 min at 4 °C. Pelleted nuclei were re-suspended in wash buffer and immediately processed with the 10X Chromium platform following the Single Cell 3' v2 protocol. After library generation with the 10X Genomics platform, libraries were sequenced on an Illumina NovaSeq S2 at a sequencing depth of 50,000 reads per cell. Reads were aligned to the GRCh37 human genome reference with GENCODE v19 gene annotations [46] using STARsolo v2.7.5 a[34] with the GeneFull argument to account for unspliced mRNA.

### SnRNA-seq data processing and identification of cell type marker genes

We then clustered the droplets using Seurat v3.2.3 [47]. In order to remove droplets contaminated with background RNA, we ran DIEM [48]. After applying filtering, we only considered droplets with at least 200 UMI and 200 genes detected [49] to ensure that each droplet had enough information for clustering and droplets with at most 20,000 UMI and the percentage of reads that map to the mitochondrial genome less than 20 to remove doublets and contaminated droplets. The count data were log-normalized using the NormalizeData function in Seurat, using the default scaling factor of 10,000. The counts for the fifteen adipose tissue samples were merged at this step. The top 2000 variable genes were then calculated using the FindVariableFeatures function.

Normalized read counts for each gene were scaled to mean 0 and variance 1. We calculated the first 30 PCs to use them as input for clustering. We then ran the Seurat functions FindNeighbors and FindClusters with 30 PCs. In the FindClusters function, we used the default parameters with standard Louvain clustering and a default clustering resolution of 0.8.

Cell type annotation was done for each droplet using SingleR v1.2.4 [50]. We used normalized expression data from BLUEPRINT [51], ENCODE [52], and the Database for Immune Cell Expression [53] that are available in the SingleR package as reference datasets. We also included snRNA-seq data of adipose tissue from sixteen individuals as a reference where cell type of each cluster was manually annotated based on cluster marker genes. Cell type labels across the reference datasets were harmonized using the SingleR function matchReferences. We removed droplets with unassigned cell type and cell types with less than 10 droplets in each cluster.

To identify marker genes for each cell type and cluster, we ran a Wilcoxon rank-sum test using the function FindAllMarkers with default parameters and only.pos = TRUE. We corrected for multiple testing using Bonferroni corrected *p* < 0.05.

### T2D GWAS in the UKB

To identify individuals with T2D in UKB [27], we selected the individuals who were diagnosed with diabetes (UKB data field 2443) or took medication for diabetes (data field 6153) as T2D cases, while removing the individuals with age of onset of diabetes (data field 25288) < 40 years to avoid inclusion of type 1 diabetics in the GWAS analysis. We excluded the individuals with missing information for diagnosis of diabetes (data field 2443) from the GWAS analysis, and then used the individuals who were not diagnosed using these relevant data fields (data fields 2443, 6153, and 25288) as the controls. To account for population stratification, we selected the unrelated, Caucasians (total *n* after the exclusions = 389,738) and used BOLT-LMM [54] to perform the GWAS associations between the genotypes and T2D status. We included age, age^2^, sex, array type, center ID, and 20 genotype PCs as covariates in the GWAS analysis.

### Stratified LD score regression

We performed stratified LD score regression using the LD Score software v1.0.0 [55, 56]. This analysis was conducted using the GWAS summary statistics from the UKB and GIANT meta-analyses for WHRadjBMI (males, females, and both sexes combined) (*n* = 315,284; *n* = 379,501; *n* = 694,549, respectively) and BMI (*n* = 806,834 both sexes combined) [15] as well as GWAS summary statistics from the UK Biobank for T2D (males, females, and both sexes combined) (*n* = 178,809; *n* = 210,929; *n* = 389,738, respectively). We partitioned the heritability into a category with the *cis* regions (± 500 kb from the ends of the gene) around the 347 WHRadjBMI co-expression network genes and the 53 standard, overlapping categories used in the LD Score software v1.0.0 [55, 56]. Briefly, the 53 functional categories are derived from 26 main annotations that include coding regions, untranslated regions (UTRs), promoters, intronic regions, histone marks, DNase I hypersensitivity sites (DHSs), predicted enhancers, conserved regions, and other annotations. The partitioned LD score regression method utilizes GWAS summary statistics of all variants to estimate how much variants in different annotation categories explain of the heritability of *cis* expression while accounting for the linkage disequilibrium (LD) among variants.

### Construction of polygenic risk score

We constructed the polygenic risk scores (PRSs) for WHRadjBMI using the same method for construction of PRSs as outlined for BMI in Khera et al. [28]. Briefly, we used the summary statistics from the GIANT GWAS for WHRadjBMI (*n* = 224,459) [8] and a reference panel of the 503 European individuals from the 1000 Genomes phase 3 version 5 [36]. We constructed nine candidate scores using the software, LDPred v1.0.6 [57], which adjusts the effect sizes for each variant in the GWAS based on LD structure. Due to the large number of participants, unified recruitment design and phenotypic characterization, the UKB is an ideal cohort for construction and testing of PRSs. Therefore, we tested and validated these candidate scores by dividing the UKB (unrelated, Caucasian individuals, *n* = 392,551) [27, 29] into 2 groups: a testing set consisting of 1/3 of the individuals (*n* = 130,851), and a validation set containing the remaining individuals unused in the testing set (*n* = 261,700). Since the fraction of causal variants is not known a priori, we tested a different value of a tuning parameter (*ρ* = 1, 0.3, 0.1, 0.03, 0.01, 0.003, 0.001, 0.0003, 0.0001), as suggested by LDPred v1.0.6 [57], in each of our nine candidate scores. We selected the best score by correlating the PRS with WHRadjBMI using Pearson correlation, which corresponded to *ρ* = 0.01. We also compared this to five PRS scores constructed using the standard method of PRS construction of LD clumping (LD *r*^2^ < 0.2) and *p* value thresholding (*p* < 0.5, 0.1, 0.05, 1 × 10^−5^, 5 × 10^−8^), as suggested by LDPred v1.0.6 [57], to confirm that using the tuning parameter constructed a superior PRS. To avoid the influence of technical factors, we corrected WHRadjBMI in the UKB for age, age^2^, sex, array type, center ID, and 20 genotype PCs. To perform statistical tests, we divided the PRS into 20 quantiles and calculated odds ratio of number of individuals in the top 10th percentile of WHRadjBMI for males and females separately.

### Prediction of type 2 diabetes using the WHRadjBMI PRS

We constructed a linear model to perform logistic regression using the binary T2D status as the outcome in the UKB validation set (*n* = 261,700) that we originally employed to validate the PRSs for WHRadjBMI. We selected the individuals who were diagnosed with diabetes (UKB data field 2443) or took medication for diabetes (data field 6153) as T2D cases, while removing the individuals with age of onset of diabetes (data field 25288) < 40 years to avoid inclusion of type 1 diabetics, with remaining individuals identified as controls. To examine individuals in the extremes of the WHRadjBMI spectrum, we selected the UKB participants in the highest (top 10% of network PRS scores) and lowest decile (lowest 10% of network PRS scores) of WHRadjBMI, as determined by the network PRS and divided them by sex. To avoid influence from the original phenotype, WHRadjBMI, as well as any technical factors, our linear model also included WHRadjBMI in addition to the network PRS score, with WHRadjBMI corrected for age, age^2^, sex, array type, center ID, and 20 genotype PCs. We performed a Wald test for the significance of each predictor in the linear model.

### Transcriptome-wide association studies (TWAS)

To identify TFs causal for WHRadjBMI, we performed a targeted transcriptome-wide association study (TWAS) [58] using GTEx v8 cohort’s subcutaneous (*n* = 581) RNA-seq data [41] to compute the TWAS weights for variants within the *cis* region (± 500 kb from the ends of the gene) around the 14 TFs in the identified WHRadjBMI co-expression network. As there are no currently TWAS functional weights for genes using GTEx v8 cohort [41] and it has significantly more samples than the GTEx v7 cohort [42] for adipose tissues, we computed our own weights using the recommended parameters by TWAS [58]. Briefly, to only include variants that will be used in the final association between TWAS and the GWAS trait, variants in the *cis* region around our 14 TFs were pruned base on the LD reference panel from the TWAS website that was converted by matching variants from GRCh37 to GRCh38 in European individuals from the 1000 Genomes phase 3 version 5 [36]. TWAS [58] checks the heritability (*p* < 0.01) and then looks for the best model out of the five standard models to estimate weights for the variants to predict gene expression. To show that the genes computed by TWAS [58] are causal for a WHRadjBMI, we then associated the TWAS model with the weighted variants with WHRadjBMI using the GWAS summary statistics from the UK Biobank and GIANT meta-analysis [15]. The use of these extensive GWASs (total *n* ~ 700,000 Europeans) should maximize power for association.

### Fine-mapping TWAS results using FOCUS

Recent work [59, 60] has shown that TWAS signal at genomic risk regions will be correlated across genes as a result of linkage disequilibrium and prediction weights, which makes distinguishing non-relevant genes from their causal counterparts challenging. To adjust for the correlation in our TWAS test statistics and identify likely causal genes, we applied FOCUS [59], a recently developed method that models the complete correlation structure within a region to fine-map TWAS signal. FOCUS models the state of genes as “causal” and “non-causal” and performs Bayesian inference over this state variable given the data. Specifically, given *m* TWAS *z*-scores ***z*** at a genomic risk region, let **Σ = Σ**(**W**, **V**) be the correlation structure of predicted expression as a function of the *m* × *p* prediction weight matrix **W** and the *p* × *p* LD matrix **V** and let ***c*** be a binary vector indicating causal status. FOCUS models the likelihood of the calculated *z*-scores ***z*** as,

$$ \Pr \left(\boldsymbol{z}\ \right|\mathbf{W},\mathbf{V},\boldsymbol{c},{\sigma}_{\alpha}^2\Big)=\boldsymbol{N}\left(\mathbf{0},\boldsymbol{\Sigma} {\boldsymbol{D}}_{\boldsymbol{c}}\boldsymbol{\Sigma} +\boldsymbol{\Sigma} \right) $$

where ${\mathbf{D}}_{\mathbf{c}}=\operatorname{diag}\left({\sigma}_{\alpha}^2\cdotp \boldsymbol{c}\right)$ is a diagonal matrix indicating which genes are causal weighted by the variance of their effect sizes. To infer the causal configuration ***c***, FOCUS computes the posterior probability as

$$ \Pr \left(\boldsymbol{c}\ \right|z,W,V,{\sigma}_{\alpha}^2\Big)=\frac{\Pr \left(\boldsymbol{z}\ \right|\mathbf{W},\mathbf{V},\boldsymbol{c},{\sigma}_{\alpha}^2\Big)\Pr \left(\boldsymbol{c}|\theta \right)}{\sum_{\mathrm{c}}^{\prime}\Pr \left(\boldsymbol{z}\ \right|\mathbf{W},\mathbf{V},\boldsymbol{c}^{\prime },{\sigma}_{\alpha}^2\Big)\Pr \left(\boldsymbol{c}^{\prime }|\theta \right)} $$

To collapse the probability over configurations ***c*** to individual genes, FOCUS computes the marginal posterior inclusion probability (i.e., PIP) at the *i*th gene as $\Pr \left({\boldsymbol{c}}_{\boldsymbol{i}}=1\ \right|\boldsymbol{z},\mathbf{W},\mathbf{V},{\sigma}_{\alpha}^2\left)={\sum}_{c:{c}_i=1}\Pr \left(\boldsymbol{c}\ \right|\boldsymbol{z},\mathbf{W},\mathbf{V},{\sigma}_{\alpha}^2\right).$ Lastly, to reflect the inherent uncertainty of inference, FOCUS computes credible gene sets for a specified credible level. For example, a calibrated 90%-credible gene set contains the causal gene with probability 90%.

### Differential gene expression analysis in the KOBS cohort

Using read counts from featureCounts v2.0.0 [61], we performed differential expression (DE) analysis using the edgeR v3.24.3 package [62]. We first performed TMM normalization using the *calcNormFactors* and variance stabilization using voom [63], and then built a linear model using LIMMA v3.38.3 [64] with the blocking factor for the baseline and follow-up measurement time points in KOBS. As with the METSIM data, to avoid the influence of the mitochondrial read number on the data, we excluded the mitochondrial reads when obtaining technical factors. Technical factors were determined by STAR v2.5.2 [37] and Picard Tools v2.9.0 (option CollectRNAseqMetrics) and included in the linear model in LIMMA v3.38.3 [64], with DE genes passing FDR < 0.05 considered as significant.

### *Cis*-eQTL analysis

We performed *cis*-eQTL analyses in the KOBS cohort [30–33] at the baseline time point using the subcutaneous adipose RNA-seq data from individuals before bariatric surgery (*n* = 262). We filtered the subcutaneous adipose RNA-seq expression data (FPKMs) to genes expressed (FPKM> 0) in greater than 90% of individuals and employed PEER factor [65] analysis to remove hidden confounders. We conducted PEER factor [65] optimization on chromosome 20 to maximize power for discovery for eQTLs, while ensuring hidden confounders were removed, and thus ended up correcting the KOBS expression data for 21 PEER factors. The KOBS cohort was genotyped using the OmniExpress (Illumina) genotyping array. We imputed genotypes using the Michigan Imputation Server [66] and filtered genotypes for variants MAF < 5% and those failing Hardy-Weinberg Equilibrium test (*p* > 1 × 10^−6^) using PLI NK v1.9 [25]. We performed *cis*-eQTL analysis using Matrix-eQTL [67], classifying variants as *cis* if they were within 1 Mb of either end of the gene.

### *Trans*-eQTL analysis of the WHRadjBMI co-expression network

We performed *trans*-eQTL analysis for the WHRadjBMI co-expression network using the genotypes for the *TBX15 cis*-eQTL WHRadjBMI GWAS SNP, rs1779445, and the eigengene of the WHRadjBMI co-expression network. The eigengene of the WHRadjBMI co-expression network was extracted from WGCNA [39] and is defined as the first principal component of the corrected gene expression used for WGCNA [39] (see “Methods” for WGCNA). A linear model was used to test the association between the genotype and eigengene.

### TBX15 motif enrichment in promoters of WHRadjBMI co-expression network genes

We used Hypergeometric Optimization of Motif EnRichment (HOMER, v4.9) [68] to search for the presence of a TBX15 motif in the promoters of the 347 WHRadjBMI co-expression network genes. We defined promoters as 2 kb upstream and 1 kb downstream of the transcription start site (TSS). The TBX15 motif was downloaded from the JASPAR database [69] and input as a custom motif into HOMER [68]. The motif finding function in HOMER [68] was used for identification of motifs in the WHRadjBMI co-expression network gene promoters.

### Human primary preadipocyte culture

Human subcutaneous primary white preadipocytes were obtained from Zen-Bio (lot L120116E, female, age 52, BMI 26.5) or PromoCell (lot 403Z001.1, male, age 30, BMI 30). Cells were maintained in a monolayer culture at 37 °C and 5% CO_2_ using preadipocyte growth medium (PromoCell C-27410) with 1% Gibco Penicillin-Streptomycin (Thermo Fisher 15140122) and following PromoCell preadipocyte culturing protocols.

### Small interfering RNA (siRNA)-mediated knockdown of *TBX15*

We knocked down *TBX15* in human subcutaneous primary preadipocytes obtained from Zen-Bio (lot L120116E, female, age 52, BMI 26.5). Human primary preadipocytes were used because they have a higher siRNA transfection efficiency than primary adipocytes. For the siRNA transfection, we used the Dharmacon SMARTpool ON-TARGETplus Human *TBX15* siRNA (L-022116-02) and the Dharmacon siGENOME Non-Targeting siRNA Pool #1 (D-001206-13) as the negative control (NC). We optimized the siRNA concentration and transfection volumes and then performed two independent siRNA transfection experiments in the human primary white preadipocytes. We used Invitrogen Lipofectamine RNAiMAX (Thermo Fisher 13778150) to transfect 50 nM of the *TBX15* or NC siRNAs using reverse transfection. Specifically, we followed the manufacturer’s instructions for diluting the siRNA and Lipofectamine RNAiMAX in Gibco Opti-MEM I Reduced Serum Medium (Thermo Fisher 31985062) and forming the siRNA-Lipofectamine RNAiMAX complexes. We incubated cell suspensions in the complexes plus serum- and antibiotic-free media (PromoCell C-27417 basal media with supplement kit components minus the fetal calf serum) to a final siRNA concentration of 50 nM. We incubated the transfection reaction at room temperature for 10 min before plating 250 μl per replicate into 12-well plates, for a total of 5 replicates per siRNA (*TBX15* and NC). After 24 h of transfection, we added 1 ml of complete preadipocyte growth medium (PromoCell C-27410). Twenty-four hours later, the media was removed and the cells were washed with PBS once prior to being treated with Invitrogen TRIzol reagent (Thermo Fisher 15596026). We performed RNA extraction per the manufacturer’s protocol using the Direct-zol RNA Mini-Prep (Zymo Research R2061).

For the two independent knockdown experiments, we confirmed by RT-qPCR that *TBX15* expression was reduced by an average of > 60% for the first experiment and 70% for the second experiment. We synthesized cDNA from 500 ng of RNA using the Applied Biosystems High-Capacity cDNA Reverse Transcription Kit (Thermo Fisher Scientific 4368814). We measured relative gene expression by RT-qPCR using an Applied Biosystems QuantStudio 5 detector, using the Bioland 2x qPCR Master Mix (Bioland Scientific, LLC QPO1-01) and following the manufacturer’s instructions. To determine the relative percent of *TBX15* expression knockdown in the preadipocytes transfected with the *TBX15* siRNA compared to the NC siRNA, we normalized expression levels to *36B4*. Primers for *TBX15* were obtained from Arribas et al. [70] and validated in-house. Primer sequences are listed below.

**Table:** 

Gene	Primer	Primer sequence
*TBX15*	Forward	5′- AAAGCAGGCAGGAGGATGTT-3′
	Reverse	5′- GCACAGGGGAATCAGCATTG-3′
*36B4*	Forward	5′-CCACGCTGCTGAACATGCT-3′
	Reverse	5′-TCGAACACCTGCTGGATGAC-3′

### RNA sequencing and differential expression analysis of siRNA mediated knockdown of *TBX15*

We submitted the RNA samples from the experiment with an average of 70% knockdown for RNA-seq. Libraries were prepared using the Illumina TruSeq Stranded mRNA kit and sequenced on an Illumina HiSeq 4000 instrument across 2 lanes for an average sequencing depth of 67 M reads (± 2.5 M reads) per sample. Reads were aligned to hg19 with STAR v2.7.0e [37], using the 2-pass method and the following parameters: --outFilterMultimapNmax 1, --outFilterMismatchNmax 6, --alignIntronMin 20, --alignIntronMax 500000, --chimSegmentMin 15.

We used the R package sva v3.26.0 [71] to estimate surrogate variables for unknown sources of variation in the data. We confirmed that the first surrogate variable (sv1) estimated using the svaseq [72] method is correlated with technical factors known to contribute to variance in RNA-seq data, such as library size, uniquely mapped read percent, and 3′ bias, as well as the gene expression first principal component. The various technical factors were obtained from STAR v2.7.0e [37] after sequence alignment (uniquely mapped reads) or from the Picard Tools v2.9.0 (option CollectRnaSeqMetrics). We used the sv1 as a covariate in the differential expression (DE) analysis.

We performed the DE analysis using the R package limma v3.34.9 [64, 73] and the voom [63] method, including sv1 as a covariate, to identify genes in the WHRadjBMI co-expression network (*n* = 347) that are significantly DE in the *TBX15* knockdown compared to the NC, with FDR < 0.05 considered as significant.

## Results

### Discovery of WHRadjBMI-associated co-expression networks in human adipose tissue

**Fig. 1:**
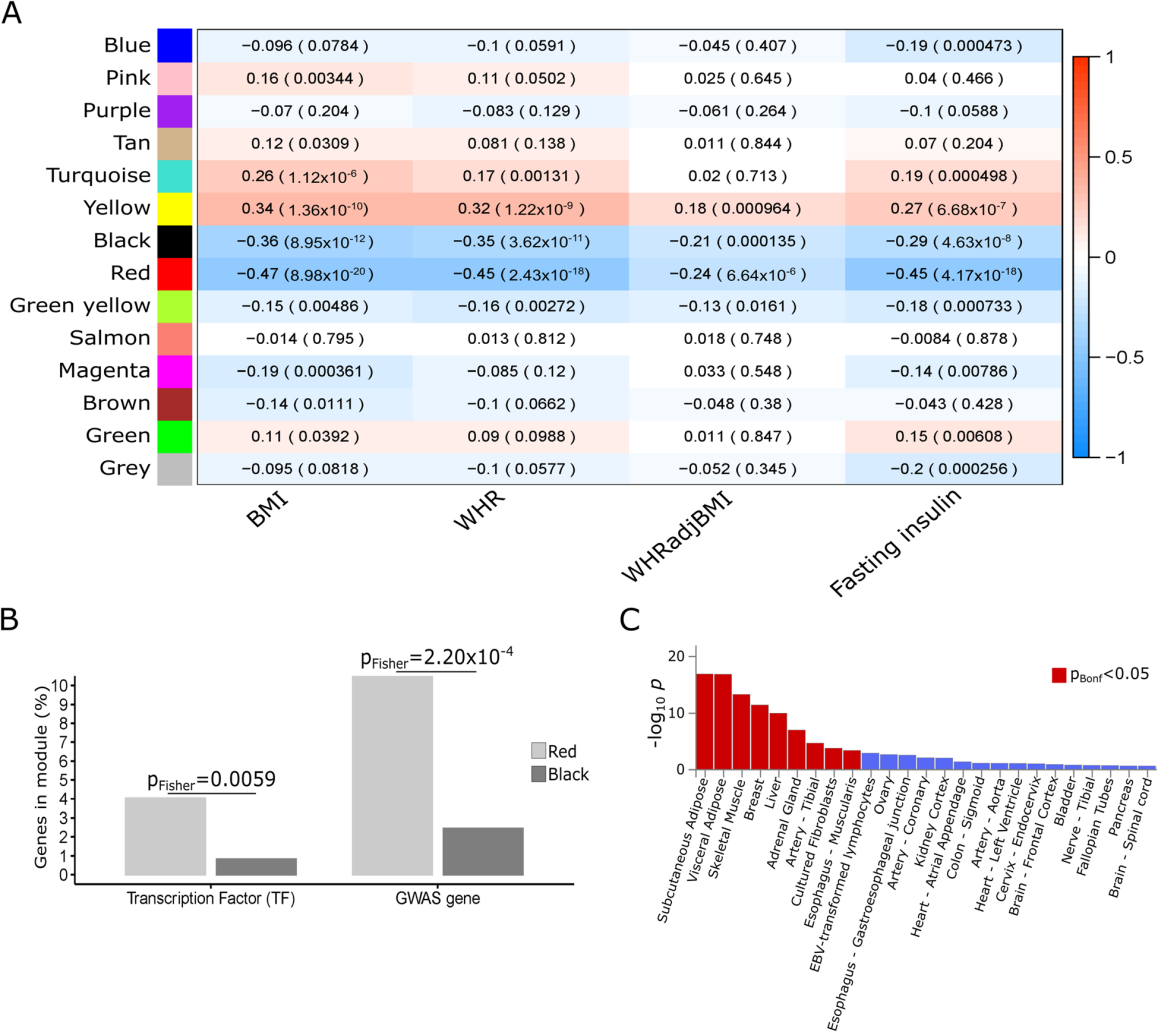
WGCNA [39] identifies 2 co-expression networks in the METSIM adipose RNA-seq cohort (*n* = 335), significantly correlated with WHRadjBMI and fasting serum insulin (**A**), discovery of the red WHRadjBMI co-expression network that is enriched for TFs and GWAS genes (**B**), and enriched for upregulated adipose tissue -specific DE genes when compared to other tissues in GTEx [41] (**C**). **A** The numbers in the cells represent Pearson correlation results of network eigengenes with BMI, WHR, and WHRadjBMI, and fasting serum insulin (adjusted for T2D status) with correlation coefficients and *p* values (shown in parenthesis). Associations that pass Bonferroni correction for the number of networks and traits tested (*p*_Bonf_ < 8.93 × 10^−4^) were considered significant. **B** Bar plot showing enrichment of TFs and GWAS genes in the red WHRadjBMI co-expression network (light gray) when compared to the black WHRadjBMI co-expression network (dark gray) using Fisher’s exact test. Significance of enrichment using Fisher’s exact test is indicated above each set of bars, *p*_Fisher_. **C** Bar plot showing significant enrichment (red) of upregulated adipose tissue-specific DE genes in WHRadjBMI co-expression network using FUMA [75, 76] when compared to the 54 other tissues in the GTEx v8 cohort [41]. GTEx v8 tissues are ranked by enrichment from most enriched to least enriched with the first 25 most enriched tissues shown. The tissue enrichments passing a Bonferroni correction are shown in red, while the non-significant enrichments are shown in blue

In our network analysis, we used waist-hip ratio adjusted for body mass index (WHRadjBMI) as a surrogate for abdominal adiposity and fat [9–11], supported by previous GWASs that have demonstrated WHRadjBMI as a more relevant adipose tissue-related obesity trait than BMI [16, 74]. To identify co-expression networks correlated with abdominal fat and adiposity, we performed weighted gene co-expression network analysis (WGCNA) (Additional file 1: Fig. S1) in the subcutaneous adipose RNA-seq data (*n* = 335) from the Finnish METabolic Syndrome In Men (METSIM) [26] cohort, which has additional measures of adiposity aside from BMI, including WHR. We identified 14 co-expression networks, two of which, red and black (colors assigned to networks by WGCNA arbitrarily), were significantly inversely correlated with WHRadjBMI, WHR, and BMI after adjusting for multiple testing (*p*_Bonf_ < 8.93 × 10^−4^) (Fig. 1A, Additional file 1: Fig. S2). To also examine if the WGCNA co-expression networks are associated with glucose metabolism, we correlated them with fasting serum insulin levels and observed significant inverse correlation of the red and black co-expression networks (*p*_Bonf_ < 8.93 × 10^−4^) (Fig. 1A, Additional file 1: Fig. S2). The red co-expression network, with 347 genes (Additional file 2: Table S1), contained 35 (10.09%) obesity GWAS genes for BMI, waist circumference (WC), WHR, WHRadjBMI, and WCadjBMI (Fisher’s exact test for the red co-expression network GWAS enrichment, odds ratio = 5.05, *p* = 2.20 × 10^−4^), whereas no such obesity GWAS gene enrichment was observed with the black co-expression network (Fig. 1B, Additional file 2: Table S2). The exact adipose expression correlation of each gene with WHRadjBMI and fasting serum insulin levels is shown in Table S1 (Additional file 2: Table S1).

Since WGCNA co-expression networks may be influenced by different cell types present in heterogeneous tissues such as adipose, we used adipose single-nuclei RNA-seq (snRNA-seq) from Finnish individuals (*n* = 15) [32, 33, 43, 44] to identify marker genes for the key adipose cell types, such as adipocytes, preadipocytes, and macrophages (Additional file 2: Table S3). The red co-expression network was enriched for adipocyte marker genes (37 adipocyte marker genes among the 347 network genes, *p*_hypergeometric_ = 8.86 × 10^−20^) (Additional file 2: Table S4), including the adipocyte secreted adipokine, adiponectin (*ADIPOQ*), indicating the importance of this co-expression network for adipocyte biology. However, the 347 red co-expression network genes also contain marker genes from other adipose cell types (Additional file 2: Table S4). Furthermore, clustering of adipose single-nucleus RNA-seq data shows that *TBX15* is expressed in most adipose tissue cell types (Additional file 1: Fig. S3). These results suggest that TBX15 likely regulates genes in multiple adipose cell types. The red co-expression network was also significantly enriched for key adipose-related metabolic KEGG pathways using WebGestalt [75, 76], such as PPAR signaling pathway, fatty acid metabolism and degradation, and valine, leucine, and isoleucine degradation (FDR < 0.05; Additional file 2: Table S5), and for GO cellular-component mitochondrion-related genes (FDR < 0.05; Additional file 2: Table S6). Furthermore, the red co-expression network was significantly enriched for genes upregulated in subcutaneous adipose tissue (*p* ~ 1.0 × 10^−18^) when compared to the 54 other tissues in Genotype-Tissue Expression (GTEx) v8 cohort [41] in a differential expression (DE) analysis by FUMA [75, 76] (Fig. 1C). Due to the significant enrichment of obesity GWAS genes, adipose-related functional pathways, adipocyte cell type marker genes, and adipose tissue-expressed genes, we focused on the red WHRadjBMI co-expression network for subsequent analyses.

### The WHRadjBMI gene co-expression network is genetically associated with WHRadjBMI and T2D

To find genetic evidence for the observed link between the co-expression network and WHRadjBMI, we examined whether the 347 co-expression network genes contribute significantly to WHRadjBMI trait heritability. We used the stratified LD score (LDSC) regression method (see “Methods”) to calculate the WHRadjBMI heritability explained using the WHRadjBMI GWAS summary statistics for all variants in the *cis* regions of the 347 genes (± 500 kb from the ends of the gene). These variants will be referred to henceforth as the WHRadjBMI *cis*-variant set. We found that these *cis* regions are significantly enriched for variants explaining the heritability of WHRadjBMI (enrichment = 1.61, *p* = 4.90 × 10^−5^) and T2D (enrichment = 1.49, *p* = 9.56 × 10^−3^) but not significantly enriched for variants explaining the heritability of BMI (*p* > 0.05) (Additional file 2: Table S7). These summary-level findings indicate that the 347 co-expression network genes and their *cis* variants are specifically important in controlling abdominal fat and adiposity and contributing to the clinical metabolic outcome, T2D.

To investigate how the WHRadjBMI co-expression network genes predict individual risk for elevated WHRadjBMI compared to the entire genome, we constructed two separate polygenic risk scores (PRSs) for WHRadjBMI: a genome-wide PRS and a network PRS with just the variants in the WHRadjBMI *cis*-variant set. For these PRS analyses, we used the UK Biobank (UKB) cohort [77] and divided the unrelated Caucasian participants into a test (*n* = 130,851) and validation (*n* = 261,700) set (see “Methods” for building the PRS).

**Fig. 2:**
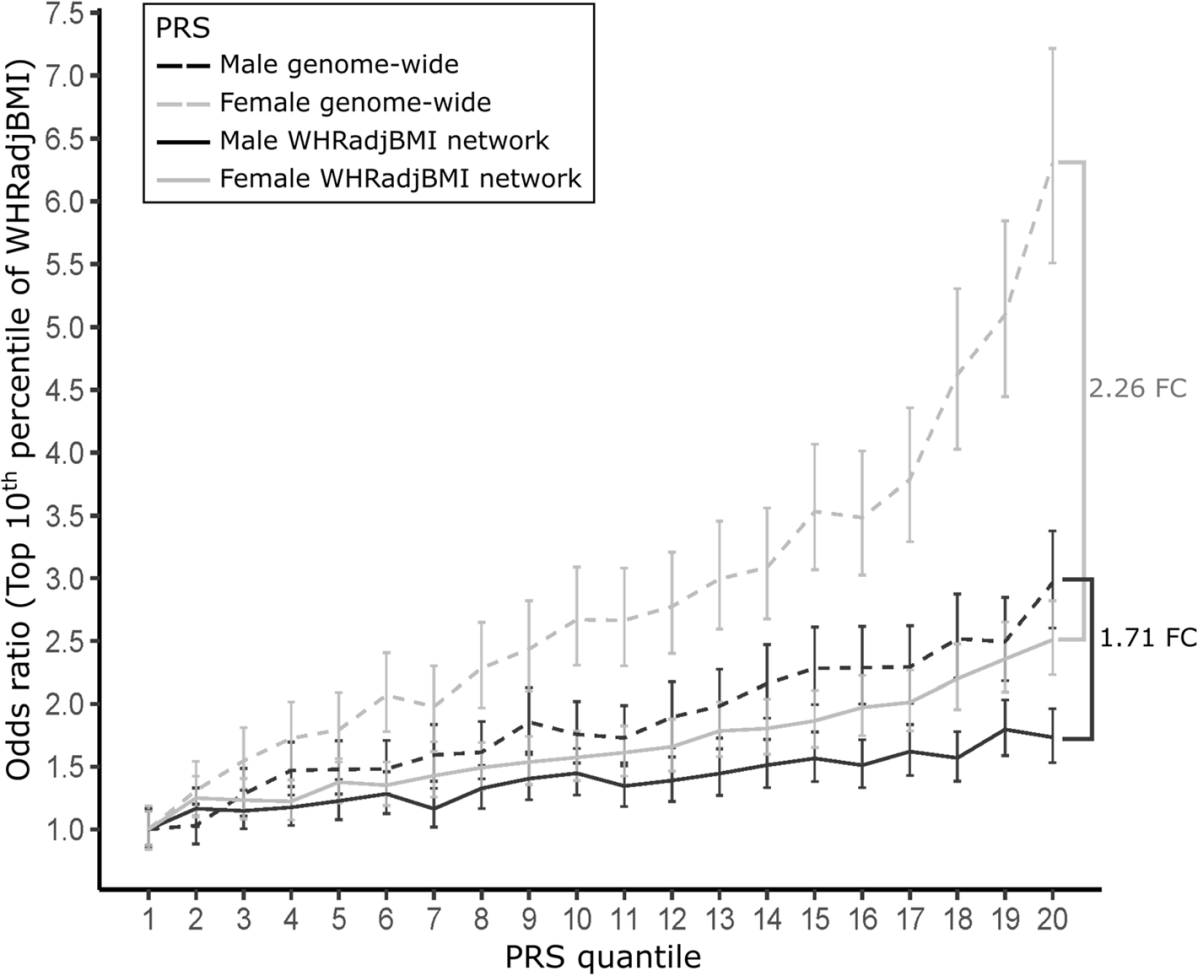
PRS scores confirm sexual dimorphism of WHRadjBMI and demonstrate the importance of WHRadjBMI co-expression network genes for WHRadjBMI in males. Plot of the PRS for WHRadjBMI in the testing set of the UK Biobank [27] (*n* = 261,700) separated for males (dark gray) and females (light gray) as well as for genome-wide PRS (dashed lines) and WHRadjBMI co-expression network PRS (solid lines; i.e., variants within the *cis* regions of the 347 co-expression network genes (± 500 kb from the ends of the gene)). Odds ratio is calculated based on the proportion of individuals in the top 10th percentile of WHRadjBMI for males and females in each of the 20 quantiles of the PRS separately. Vertical error bars indicate the 95% CI for the odds ratio. Brackets show a fold change (FC) in the odds ratio for the 20th quantile

To investigate the effectiveness of our genome-wide PRS in predicting WHRadjBMI with the validation set (*n* = 261,700) (PRS correlation coefficient with WHRadjBMI = 0.206), we divided the individuals into 20 quantiles based on their PRS scores and then by sex. Next, we calculated the odds ratio of being in the top 10th percentile of WHRadjBMI, for individuals in each of the 20 quantiles compared to the lowest quantile. As expected based on the previous GWAS studies examining the differences in heritability of WHRadjBMI between the sexes [16] and our results from LDSC (Additional file 2: Table S7), the genome-wide PRS predicts WHRadjBMI better in females than males (females: 6.31-fold increase in risk for elevated WHRadjBMI between the lowest quantile and the 20th quantile of the PRS versus males: 2.96-fold increase in risk for elevated WHRadjBMI) (Fig. 2).

Notably, despite the fact that the network PRS only comprises the variants in the *cis* regions of the 347 co-expression network genes, having thus many fewer variants included, the network PRS correlation coefficient with WHRadjBMI was 0.110 (compared with the genome-wide PRS correlation coefficient of 0.206, which is less than twice that of the network PRS). Although both the genome-wide PRS and network PRS are more predictive of WHRadjBMI in females (Cochran-Mantel-Haenszel test on the 20th quantile, genome-wide PRS versus network PRS and males versus females, ${\chi}_{\mathrm{CMH}}^2$=1146.94, *p*_CMH_ = 2.07 × 10^−251^), the power decrease from using the genome-wide PRS to using the network PRS is much greater for females (20th quantile odds ratio: 2.51-fold decrease) when compared to males (20th quantile odds ratio: 1.71-fold decrease) (Fig. 2). This suggests that, relative to the genome-wide PRS, the 347 co-expression network genes and their *cis* variants constitute a larger percentage of the predicted effect of variants for regulating WHRadjBMI in males when compared to the same PRS predictions in females.

To provide additional evidence that the network PRS is more informative and biologically important in males than females, we tested whether males with the highest genetically predicted WHRadjBMI (based on the network PRS) are more likely to have the clinically relevant metabolic outcome of T2D. Accordingly, we selected individuals with the network PRS in the highest and lowest deciles (top 10% and lowest 10% network PRS scores), as done previously for BMI in Khera et al. [28], and divided them by sex. We used a logistic regression (see “Methods”) and when accounting for WHRadjBMI in our model, observed that the network PRS significantly predicted T2D in males (*β* = 1.12, *p* = 9.59 × 10^−5^) but not in females (*p* > 0.05). These results indicate that the 347 co-expression network genes and their *cis* variants significantly contribute to the clinical metabolic outcome, T2D, in males while no such effect was observed in females. In sum, by leveraging subcutaneous adipose RNA-seq data from a cohort with the abdominal adiposity measure, WHR, we identified a WHRadjBMI co-expression network that genetically controls WHRadjBMI and T2D in a sex-dependent manner.

### The WHRadjBMI co-expression network connectivity is sex- and context-dependent

We hypothesized that the sex-dependent effects we observed with the network PRS for WHRadjBMI and T2D would be reflected in the co-regulation of these genes as well. We therefore tested whether the WHRadjBMI co-expression network connectivity is different between males and females in the independent GTEx v8 subcutaneous adipose RNA-seq data [41]. We performed a network preservation analysis separately in males (*n* = 387) and females (*n* = 194) (see “Methods”), and found that the network preservation *Z*_Summary_ score was 30 in males versus 22 in females. The *Z*_Summary_ score value is not sensitive to the sample size, and so the relative difference in the number of males and females was not a concern. This lower network preservation in females is in line with the lesser trait prediction observed for WHRadjBMI and T2D with the network PRS in females.

Further supporting the sex-dependent effects, we also observed an enrichment of androgen receptor element (ARE) motif (binomial test adjusted *p* value = 0.0001) in the promoters (+ 2 kb/− 1 kb from TSS) of the WHRadjBMI co-expression network genes when compared to the promoters of all genes expressed in the METSIM adipose RNA-seq data using HOMER [68]. Additionally, we investigated whether adipose expression of androgen receptor (*AR*), estrogen receptor 1 (*ESR1*), aromatase (CYP19A1), or sex hormone-binding globulin (SHBG) were correlated with the *TBX15* adipose expression in METSIM. We found the following correlations between the *TBX15* adipose expression and these genes: *AR* (*r* = 0.164, *p*_*Pearson*_ = 0.00262), *ESR1* (*r* = 0.355, *p*_*Pearson*_ = 2.12 × 10^−11^), *CYP19A1* (*r* = 0.355 and *p*_*Pearson*_ = 2.12 × 10^−11^), respectively, even though none of these genes were present in the same co-expression network as *TBX15*. These results suggest that sex hormones may play a role in the observed sex-dependent PRS and network preservation results.

We further tested whether the WHRadjBMI co-expression network connectivity is altered context-dependently based on the obesity state. Because the GTEx cohort phenotypes do not include WHRadjBMI, we divided the cohort first by sex and then into the more extreme categories of lean (BMI < 25; *n*_Male_ = 102, *n*_Female_ = 78) and obese (BMI > 30; *n*_Male_ = 119, *n*_Female_ = 41) to increase the chance that there are differences in abdominal adiposity between the sets of individuals. We found that the network preservation *Z*_Summary_ score drastically decreased between lean and obese males (*Z*_Summary – Lean male_ = 30 versus *Z*_Summary – Obese male_ = 19) but remained similar between lean and obese females (*Z*_Summary – Lean female_ = 20 versus *Z*_Summary – Obese female_ = 18. Taken together, the network preservation results suggest that the coordinated expression of the genes in the WHRadjBMI co-expression network is regulated more tightly in males than females, and in a context-specific manner that depends on the obesity state.

We also tested the WHRadjBMI co-expression network preservation in the Mexican population, using the Mexican Obesity Study (MOSS) cohort [34]. The MOSS participants are morbidly obese individuals undergoing bariatric surgery from whom subcutaneous adipose tissue biopsies are obtained at the time of surgery (average BMI = 45.4) and at a 1-year follow-up (average BMI = 33.8) (*n* = 43 at both time points). We observed that the *Z*_Summary_ score was 30 in the baseline versus 51 in the follow-up, indicating that the WHRadjBMI network is preserved across diverse populations and responds to changes in weight.

Next, we investigated the WHRadjBMI co-expression network preservation in visceral adipose tissue and muscle RNA-seq data from GTEx [41, 42] and observed a strong preservation in visceral adipose tissue in both males (*n* = 149) and females (*n* = 84) (Additional file 1: Fig. S4), while no such strong preservation was observed in muscle (*Z* < 10 in both male (*n* = 153) and female (*n* = 145) muscle tissue). These results suggest that the WHRadjBMI co-expression network is more important for adipose tissue than muscle function.

### Identifying candidate master regulators of the WHRadjBMI-associated co-expression network

Since transcription factors (TFs) have been suggested as one possible type of genes that could drive co-expression networks in *trans* [39], we first identified all TFs (*n* = 14) in the WHRadjBMI co-expression network using the PANTHER database [78] (Additional file 2: Table S8). Next, to test which of these 14 TFs are potentially causal for WHRadjBMI and find *trans* regulator genes and candidates for our experimental follow-up, we performed a transcriptome-wide association study (TWAS), which is a method to test for association between gene expression and a trait by weighting the effects of *cis* variants on gene expression and testing their weighted association with a GWAS trait (see “Methods”). We computed eQTL weights for the variants in the *cis* region (± 500 kb from the ends of the gene) around each TF using GTEx v8 cohort data. To accurately estimate the gene expression heritability in TWAS, we used the entire GTEx subcutaneous adipose RNA-seq dataset (*n* = 581). We found that five TFs in the WHRadjBMI co-expression network that passed the TWAS heritability thresholds (*p* < 0.01) that is required for testing the association of the *cis* SNP heritability with phenotypes: T-Box Transcription Factor 15 (*TBX15*), General Transcription Factor IIE Subunit 2 (*GTF2E2*), X-Prolyl Aminopeptidase 3 (*XPNPEP3*), Iroquois Homeobox 1 (*IRX1*), and Zinc Finger Protein 3 (*ZNF3*) (Additional file 2: Table S9).

We next tested whether these five *cis*-heritable TFs are associated with WHRadjBMI using the computed TWAS weights to impute the TF gene expression and the WHRadjBMI summary statistics from the large UK Biobank (UKB) and GIANT meta-analysis GWAS data (*n* ~ 700,000). *TBX15*, *XPNPEP3*, and *IRX1* passed the Bonferroni correction for being associated with WHRadjBMI in the TWAS (*p* < 0.017) (Additional file 2: Table S10), implying that the variants contributing to the *cis-*regulation of these TFs are also important for WHRadjBMI.

**Fig. 3:**
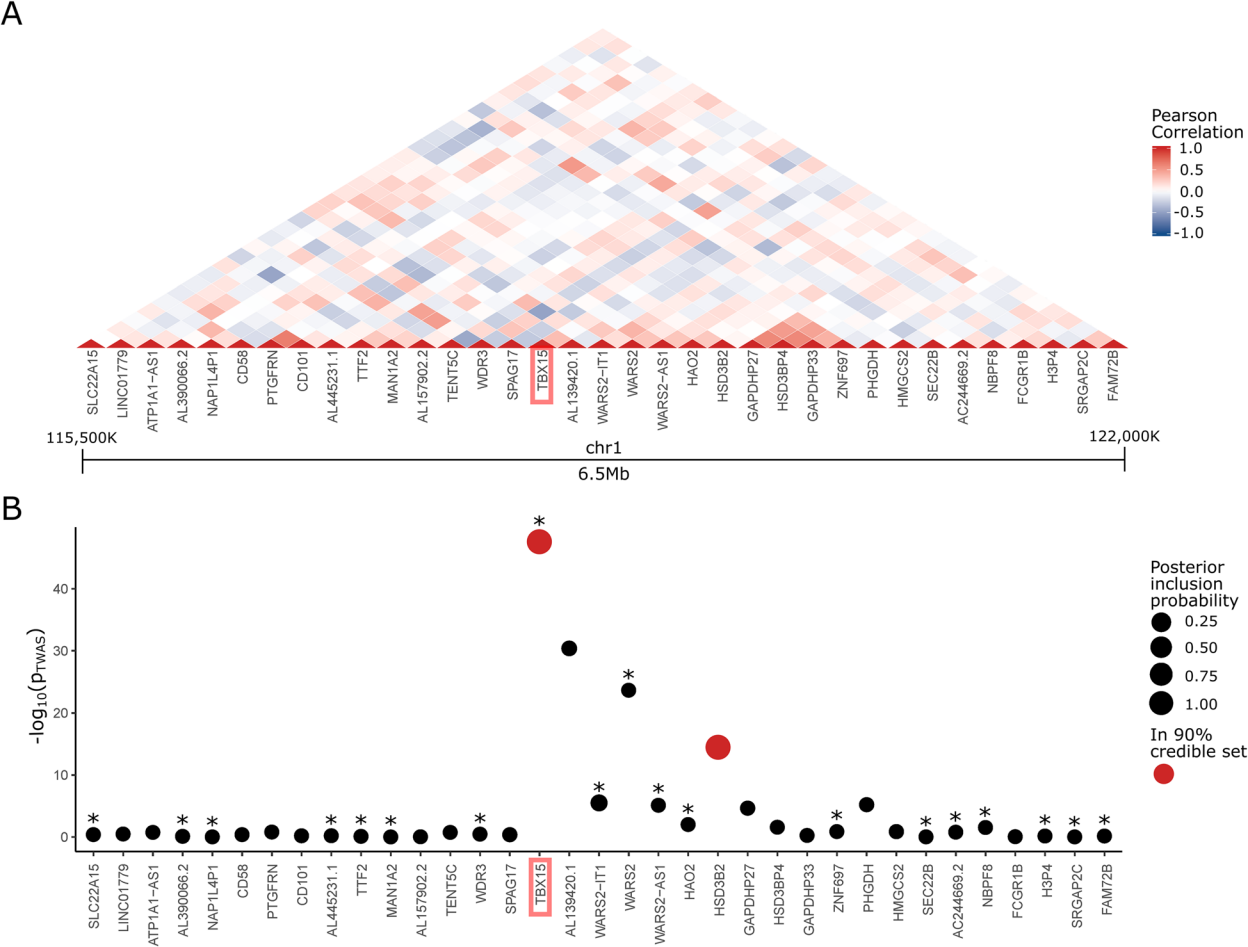
TWAS [58] and FOCUS [59] results in GTEx v8 subcutaneous adipose RNA-seq data implicates *TBX15* as the only TF in the WHRadjBMI co-expression network causal for WHRadjBMI. **A** Pairwise Pearson correlation coefficients between all genes in the *TBX15* locus (chr1:115476504-121965583) using the normalized gene expression from the GTEx v8 cohort subcutaneous adipose RNA-seq data [41] (*n* = 581). **B** Plot of −log_10_ *p* value for TWAS association with WHRadjBMI for each gene in the *TBX15* locus (chr1:115476504-121965583) with a significant heritability estimate (*p* < 0.01) in the GTEx v8 cohort genotype and subcutaneous adipose RNA-seq data (*n* = 581). Size of the point indicates the magnitude of the FOCUS marginal posterior inclusion probability (PIP). Genes included in the final 90% credible set are marked in red. Stars above points indicate a significant TWAS cross-validation *p* value (*p* < 0.01)

The interpretation of TWAS results as evidence of causality can be complicated by other regional genes that may share *cis* variants, LD structure, or co-expression with the putatively causal gene (Fig. 3A). To better determine if there is statistical support for the TWAS evidence of association between WHRadjBMI and *TBX15*, *XPNPEP3*, and *IRX1*, we used the fine-mapping of causal sets (FOCUS) tool, employing the same GTEx v8 cohort and WHRadjBMI GWAS data, and including all genes ± 3 Mb from the ends of our TFs of interest. FOCUS is a fine-mapping approach for TWAS that identifies a gene set containing the causal gene(s) in a locus at a predefined level of credibility, based on their posterior inclusion probability (PIP) of being the causal gene while accounting for shared *cis* variation among genes at a locus (see “Methods”). The FOCUS analyses showed that *TBX15* and nearby gene Hydroxy-Delta-5-Steroid Dehydrogenase, 3 Beta- And Steroid Delta-Isomerase 2 (*HSD3B2*) were included in the 90% credible set; however, only the TWAS expression heritability of *TBX15* predicted well in the cross-validation (*TBX15*: TWAS cross-validation *p* = 1.54 × 10^−7^; *HSD3B2*: TWAS cross-validation *p* > 0.05) and had a higher PIP (*TBX15*: FOCUS PIP > 0.99; *HSD3B2*: FOCUS PIP = 0.908), thus effectively fine-mapping the locus to *TBX15* (Fig. 3B). When testing *XPNPEP3* and *IRX1*, FOCUS provided little support for a causal role at current sample sizes (*XPNPEP3*: FOCUS PIP = 9.90 × 10^−5^; *IRX1*: FOCUS PIP = 0.0735). Taken together, the results from TWAS and FOCUS show statistical support for a causal role of only one of the 14 TFs in the WHRadjBMI co-expression network, *TBX15*, thus highlighting it as a candidate TF driving the WHRadjBMI co-expression network; however, we note that this result does not exclude the possibility that another type of gene other than a TF could also contribute to the co-expression in this network.

### *TBX15* and the WHRadjBMI co-expression network change in response to extreme weight loss

Support for the evidence that *TBX15* is a causal gene in regulating adiposity has been published in mouse knockout studies, where adipose-specific loss of *Tbx15* leads to increased weight gain when mice are put on a high-fat diet [79]. This suggests that, in conditions of increased energy intake, a pathological decrease in *TBX15* can drive adiposity. To test for evidence of a similar mechanism in humans, we used subcutaneous adipose RNA-seq data from the Finnish Kuopio OBesity Study (KOBS) bariatric surgery cohort [33], in which the individuals’ average BMI decreased from 43.0 to 34.3 (22.7% decrease) from the time of surgery to the 1-year follow-up (*n* = 168 at both time points). A change in WHR could not be assessed in the KOBS cohort as in general it is not possible to reliably measure waist circumference in morbidly obese individuals undergoing bariatric surgery. In these weight loss analyses, we found that *TBX15* showed a significant increase in gene expression in the 1-year follow-up (log_2_ fold change (FC) = 0.37, *p* = 1.48 × 10^−6^), indicating that *TBX15* responds to weight loss and in line with its inverse correlation with adiposity. In addition, 184 of the 347 WHRadjBMI co-expression network genes (53%) were differentially expressed between the baseline and 1-year follow-up (FDR < 0.05) (Additional file 2: Table S11). Based on the effect sizes in KOBS [33], we estimated that the small sample size of the MOSS cohort [34] (*n* = 43) does not allow for a powerful enough DE analysis to detect changes in expression of *TBX15* or the WHRadjBMI co-expression network genes.

### Identification of a WHRadjBMI co-expression network *trans*-eQTL

To investigate whether *TBX15* genetically drives the expression of the WHRadjBMI co-expression network genes in *trans*, we investigated the WHRadjBMI GWAS SNP rs1779445 (GIANT, *n* = 224,459) (β_C allele_ = 0.032, *p* = 1.60 × 10^−12^) [8], and first observed that this GWAS SNP regulates *TBX15* adipose expression in *cis* (β_C allele_ = 0.092, *p* = 0.0032 in GTEx [41]; and β_C allele_ = 0.56, *p* = 0.0047 in KOBS [33]). We recognize that the direct identification of *trans*-eQTLs requires large cohorts. To partially circumvent this, we tested whether rs1779445 regulates the eigengene of the WHRadjBMI co-expression network. We found that rs1779445 is a *trans*-eQTL of the network eigengene in the METSIM cohort [26] (*n* = 335) (β_C allele_ = − 0.019, *p* = 0.031), thus providing genetic evidence that *TBX15* contributes to the *trans* regulation of the WHRadjBMI co-expression network genes.

### Knockdown of *TBX15* in primary human preadipocytes confirms the role of TBX15 as a master regulator of the WHRadjBMI co-expression network

**Fig. 4:**
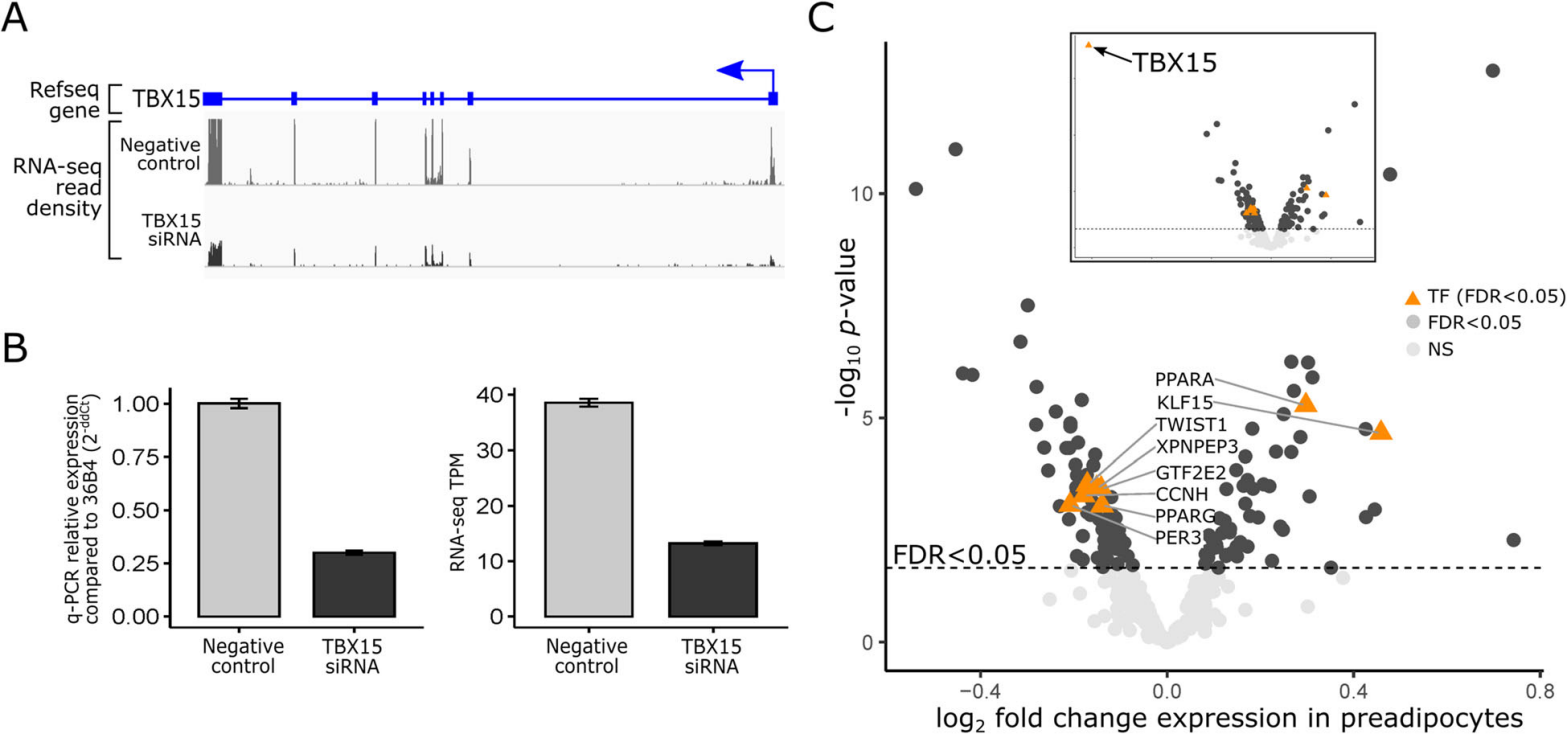
Knockdown of *TBX15* in human primary preadipocytes significantly affects 130 genes (FDR < 0.05) in the WHRadjBMI co-expression network. **A** Illustration of *TBX15* gene with introns and exons; and the relative RNA-seq read density in the human primary preadipocyte cells transfected with the negative control siRNA when compared to the cells transfected with the *TBX15* siRNA. Scales for the read density are equal. **B** Bar plot showing the qPCR relative expression (2^−ddCt^) when compared to the housekeeping gene 36B4 and RNA-seq TPMs for *TBX15* in the cells transfected with negative control siRNA when compared to the cells transfected with *TBX15* siRNA (*n* = 5). **C** Volcano plot of differentially expressed (DE) genes in *TBX15* knockdown experiment, excluding *TBX15*. Significant genes (FDR < 0.05) (dark gray), non-significant genes (light gray), and TFs (orange; FDR < 0.05) are plotted based on their log_10_ *p* value and log_2_ fold change in expression. Significantly differentially expressed TFs are labeled. Inlay shows the volcano plot of the *TBX15* DE results with *TBX15* included

To functionally confirm the role of *TBX15* as one of the WHRadjBMI co-expression network key regulators, we performed knockdown (KD) of *TBX15* via small interfering RNA (siRNA) in primary human preadipocytes (*n* = 5 isogenic replicates) (Fig. 4A). We used primary human preadipocytes instead of primary adipocytes because they have higher siRNA transfection efficiency than primary adipocytes and because preadipocytes are a critically important cell type for adipose tissue function. We successfully performed *TBX15* KD, decreasing its expression by ~ 70%, confirmed by RT-qPCR (Fig. 4B). Next, we performed RNA-seq to see if the genes in the WHRadjBMI co-expression network are affected by KD of *TBX15*. When comparing to preadipocytes transfected with the negative control siRNA (see “Methods”), we found that 130 of the 347 WHRadjBMI co-expression network genes (37.46%) are significantly DE (FDR < 0.05) between the *TBX15* KD and control, including the well-established key adipose tissue master regulators, *PPARG and KLF15* (Fig. 4C, Additional file 2: Table S12). We also found that 81 genes of the 130 DE genes (62%) in our *TBX15* KD experiment have a TBX15 motif in their promoter (+ 2 k/− 1 kb from TSS) (Additional file 2: Table S12), suggesting that TBX15 may have a direct effect on these genes.

When searching for other TFs affected by *TBX15* KD that may contribute to the wide-spread *trans* effects of *TBX15*, a total of 8 TFs of the 13 TFs (61.5%) in the WHRadjBMI co-expression network were observed to be significantly DE (FDR < 0.05) (*PPARG, PPARA, KLF15, TWIST1, XPNPEP3, GTF2E2, CCNH, PER3*) by the *TBX15* KD. This result suggests that *TBX15* affects many additional genes indirectly downstream by regulating other key adipose TFs (Fig. 4C).

In summary, these genetic and functional data discover a human adipose master *trans* regulator, *TBX15*, which in turn controls an obesity GWAS gene-enriched network that sex-dependently modifies the distribution of fat, likely due to regulation of the WHRadjBMI co-expression network genes by androgens.

## Discussion

WHRadjBMI is a well-established measure of abdominal adiposity, whereas BMI cannot reliably separate fat from lean mass [16], in line with previous GWAS studies of WHRadjBMI and BMI demonstrating that the WHRadjBMI GWAS loci are more adipose tissue related than the BMI loci in terms of their expression profiles and function [9–11]. Furthermore, while overall obesity measures like BMI do not exhibit sexual dimorphism [8], WHRadjBMI and fat distribution have clear sex-specific differences that are reflected in differences in heritability [8, 15], GWAS loci [13, 74], and ultimately in risk for disease outcomes such as T2D and cardiovascular disease [14, 28]. However, the underlying biological mechanisms that contribute to the sexual dimorphism of body fat distribution are still poorly understood. Furthermore, the genes behind complex diseases such as obesity are often regulated and dysregulated together, influencing the progression and severity of obesity [80].

In this work, we used subcutaneous adipose RNA-seq data collected in the METSIM male population cohort [26], for which we have measures of WHR, to identify a gene co-expression network that is important for regulating WHRadjBMI and exhibits the known sexual dimorphism of this trait at both a genetic and transcriptomic level. We used the UKB to show that the genetic variants in the *cis* regions of the 347 WHRadjBMI co-expression network genes are significantly enriched for variants that contribute to the heritability of WHRadjBMI and T2D, but not BMI. These variants also have a sex-dependent effect on the ability to predict elevated WHRadjBMI in males when compared to females relative to the entire genome, as shown by the genome-wide and network-specific WHRadjBMI PRSs we constructed. Furthermore, we show that the network PRS significantly predicts the disease outcome, T2D, in males but not in females, even when accounting for the effects from the original trait, WHRadjBMI. These PRS results demonstrate the sex-dependent effects of the 347 WHRadjBMI co-expression network genes and their *cis* variants on both WHRadjBMI and T2D. This sex-dependent effect is likely mediated via regulation by androgen, as suggested by our androgen receptor element (ARE) motif enrichment, in line with previous studies showing sex differences in adipose tissue function [81]. Furthermore, our gene expression correlations between *TBX15* and *AR*, *ESR1*, *CYP19A1*, and *SHBG* indicate that sex hormones may also contribute to the observed sex-dependent PRS and network preservation results. Finally, we provide genetic and functional evidence for a novel role of the TF, T-Box Transcription Factor 15 (*TBX15*), as one of the key master *trans* regulators of this WHRadjBMI co-expression network, advancing our understanding of how *trans* regulation of gene expression contributes to normal and obesity-deteriorated adipose tissue function, and the sexually dimorphic accumulation of harmful abdominal fat.

TFs form one category of genes hypothesized to regulate co-expression in networks [39]. To find potential causal drivers of the co-expression in the WHRadjBMI network and candidates for our functional follow-up, we first employed TWAS [58] and FOCUS [59] to investigate all 14 TFs present in the WHRadjBMI network, which resulted in the discovery of the TBX15 as a master *trans* regulator candidate for this WHRadjBMI network. Noteworthy, *TBX15* has been implicated in large European and smaller non-European GWAS studies for WHRadjBMI and other related obesity traits [8, 82, 83]. However, our study is the first to discover *TBX15* both as the underlying regional causal WHRadjBMI gene at the *WARS2*-*TBX15* locus utilizing TWAS and FOCUS and as the driver of the co-expression network using *trans*-eQTL and experimental siRNA validation analyses. Previous *Tbx15* studies have been conducted in mouse, showing that *Tbx15* affects the differentiation of preadipocytes to adipocytes, with reduced expression of key adipose TFs *Cebpa* and *Pparg* in mouse preadipocytes that stably overexpress (OE) *Tbx15* [84]. This mouse study also suggests that even after rescuing the induction of adipogenesis using a PPARG agonist, *Tbx15* OE cells exhibit decreased lipogenesis and increased lipolysis. These mouse results are in line with the inverse relationship of *TBX15* with WHRadjBMI that we observed, and also highlight the role of *TBX15* [84] in adipocyte differentiation. Interestingly, *Tbx15* has also been shown to regulate adipocyte browning in mice [79]. In line with this finding, previous functional studies have shown that *TBX15* affects mitochondria-related gene expression and mitochondrial mass in mice [84] and humans [85, 86], in line with the GO cellular-component enrichment of the WHRadjBMI co-expression network genes for mitochondrion-related genes. In addition to mouse knockout studies, where adipose-specific loss of *Tbx15* leads to increased weight gain when mice are put on a high-fat diet [79], these previous studies provide support for our discovery of *TBX15* as one of the key TF master regulators in human subcutaneous adipose tissue, with adiposity-driven changes in *TBX15* expression affecting its role in maintaining homeostasis of the WHRadjBMI co-expression network.

Our use of TWAS [58] and FOCUS [59] also assisted in disentangling the *TBX15-WARS2* GWAS locus [8, 82, 83] for WHRadjBMI. Since *TBX15* and *WARS2* share many of the same *cis*-eQTLs and some of the GWAS variants are intergenic, it has remained difficult to determine which gene in the locus is the underlying causal gene [8, 45]. However, TWAS [58] identified and FOCUS [59] fine-mapped *TBX15* as the significant causal gene for WHRadjBMI in the *TBX15-WARS2* locus, whereas *WARS2* was not identified as a causal WHRadjBMI gene in the locus.

We used the independent subcutaneous adipose RNA-seq data from the GTEx v8 cohort [41] and the Mexican MOSS cohort [34] to show that the WHRadjBMI co-expression network is highly preserved in diverse populations. The large GTEx cohort also allowed us to perform a sex-specific analysis, which demonstrated that males exhibit a higher network preservation than females. Furthermore, the network preservation is higher in the lean (BMI < 25) state when compared to the obese (BMI > 30) state in males, but is similar between lean and obese females. This apparent breakdown of network connectivity in the obese males supports the idea that aberrant regulation of the network as a whole develops as WHRadjBMI increases. Although the GTEx cohort [41] lacks measurements for WHRadjBMI due to the fact that it consists largely of post-mortem samples, we were able to show the sex- and obesity-dependent effects on this WHRadjBMI network using more extreme BMI cutoffs of lean (BMI < 25) and obese (BMI > 30). However, presently there are no sex-specific guidelines for the BMI cutoffs for the transition between lean, overweight, and obese states, let alone WHRadjBMI. To partially circumvent this issue and study the effects of weight differences on *TBX15* expression, we also leveraged longitudinal adipose RNA-seq data from the KOBS bariatric surgery cohort [33], which demonstrated that adipose expression of *TBX15* recovers after dramatic weight loss within an individual. These weight loss results from the KOBS cohort [33] suggest that decreased adipose expression of *TBX15* in obese individuals contributes to the observed dysregulation of the WHRadjBMI co-expression network.

Although visceral adipose tissue is known to be more strongly linked to metabolic disorders and WHRadjBMI [87, 88] than subcutaneous adipose tissue, subcutaneous adipose tissue exhibits larger changes in volume during weight loss or weight gain [89]. Furthermore, subcutaneous adipose biopsies are available through less-invasive procedures than visceral adipose tissue biopsies, which require a surgical procedure. Our results from the heritability and PRS analyses, and the context-specificity of the network preservation show that the subcutaneous adipose WHRadjBMI co-expression network is both an important driver and responder, respectively, to changes in WHRadjBMI.

To functionally verify that the WHRadjBMI co-expression network is driven by *TBX15*, we knocked down *TBX15* via siRNA in primary human preadipocytes, and performed RNA-seq to assess the effects of *TBX15* KD on the expression of all 347 co-expression network genes. Human primary preadipocytes were used as they have a higher siRNA transfection efficiency than primary adipocytes and furthermore, preadipocytes are a critically important cell type for adipose tissue function [90]. Their proper function and turnover are crucial to a balance between adipose hypertrophy and hyperplasia, and thus their dysfunction predisposes to pathogenic mechanisms contributing to cardiometabolic disorders, such as inflammation and insulin resistance. This experiment showed that knocking down *TBX15* significantly affects the downstream expression of 8 additional TFs, including the key adipose tissue TFs, *PPARG* and *KLF15*, along with 121 other co-expression network genes. We recognize the limitation that performing our experiments in human primary preadipocytes does not allow us to assess the effect of *TBX15* knockdown on genes directly involved in adipocyte differentiation and differentiated adipocytes, warranting thus additional investigations of *TBX15* knockdown during adipogenesis in the future studies. Nevertheless, to the best of our knowledge, our functional study is one of the first examples of experimental validation of a TF *trans* regulating a co-expression network in humans. Furthermore, these DE genes are enriched for the valine, leucine, and isoleucine degradation KEGG pathway using WebGestalt [75, 76]. This pathway functions in the breakdown of essential branched chain amino acids that humans only obtain in their diet. Previous studies have shown that obese individuals exhibit higher levels of these amino acids in their plasma even when matched for dietary intake or after overnight fasting, most likely due to their impaired degradation [91]. While further investigation is warranted to investigate *TBX15* at the protein level, we chose to examine knockdown of *TBX15* at the gene expression level because our discoveries of the WHRadjBMI co-expression network and the importance of *TBX15* for WHRadjBMI were done at the gene expression and genetic variant level. As it has been shown that there is significant buffering between *cis*-eQTLs and protein-QTLs (p-QTLs) [92], gene expression levels of *TBX15* may not correlate strongly with protein levels, thus possibly diluting many of the genetic and expression level signals. Taken together, these data, along with the recovery of *TBX15* expression after weight loss, indicate that *TBX15* plays an important role in maintaining the homeostasis of this subcutaneous adipose WHRadjBMI co-expression network in the non-obese state.

## Conclusions

In summary, we discovered a novel master adipose *trans* regulator, *TBX15*, and its causal effect on WHRadjBMI, with a stronger effect observed in males. We also provide insight into a WHRadjBMI co-expression network containing critical adipose TFs and GWAS genes that *TBX15* regulates, and demonstrate the large contribution of the *cis* variants of these co-expression network genes to both WHRadjBMI PRS and T2D PRS in a sex-dependent manner in the UK Biobank. Through our knockdown of *TBX15* in human primary preadipocytes, we provide concrete functional evidence showing that decreasing expression of *TBX15* directly affects expression of 130 genes in the WHRadjBMI co-expression network, including 8 key TFs, thus compounding the downstream effects on metabolically harmful abdominal obesity.

## Supplementary Information

**Additional file 1.** Supplementary figures (Fig. S1-S4)

**Additional file 2.** Supplementary tables (Table S1-S12)

## Data Availability

The data that support the findings in this manuscript are available from the UK Biobank. However, restrictions apply to the availability of these data, which were used in this study under UK Biobank Application Number 3934. UK Biobank data are available for bona fide researchers through the application process (https://www.ukbiobank.ac.uk/learn-more-about-uk-biobank/contact-us). The METSIM adipose gene expression data are available online in the Gene Expression Omnibus (GEO), under accession number GSE135134 (https://www.ncbi.nlm.nih.gov/geo/query/acc.cgi?acc = GSE135134) [26]. The GTEx subcutaneous, visceral, and muscle RNA-seq data are available from the NIH dbGAP, study number phs000424.v8.p2 (https://www.ncbi.nlm.nih.gov/projects/gap/cgi-bin/study.cgi?study_id = phs000424.v8.p2) [41]. Summary-level data for the KOBS cohort [33] are given in Table S11, and summary-level data for the Finnish Twin Study [43] and CRYO cohort [33, 44] are given in Table S3. Data access to the existing KOBS [33], MOSS [34], Finnish Twin Study [43] and CRYO [33, 44] cohorts are described in the original publications cited here for each cohort. All code used for analyses in this study were unaltered from their publicly available sources. Parameters chosen for each analysis are described in detail in Methods. Correspondence regarding availability of cellular materials and data should be addressed to the corresponding author, Päivi Pajukanta (ppajukanta@mednet.ucla.edu).

## Abbreviations

ADIPOQ: Adiponectin
ARE: Androgen receptor element
BMI: Body mass index
BP: Base pair
CEBPA: CCAAT enhancer binding protein alpha
CYP19A1: Aromatase
DE: Differential expression
DHS: DNase I hypersensitivity site
eQTL: Expression quantitative trait locus
ESR1: Estrogen receptor 1
FC: Fold change
FDR: False discovery rate
FOCUS: Fine-mapping of causal sets
FPKM: Fragments per kilobase million
GTEx: Genotype expression cohort
GTF2E2: General Transcription Factor IIE Subunit 2
GWAS: Genome-wide association study
HRC: Haplotype Reference Consortium
HSD3B2: Hydroxy-Delta-5-Steroid Dehydrogenase, 3 Beta- And Steroid Delta-Isomerase 2
INCMN: Instituto Nacional de Ciencias Medicas y Nutricion
IRX1: Iroquois Homeobox 1
KD: Knockdown
KLF15: Krüppel Like Factor 15
KOBS: Kuopio obesity study cohort
LD: Linkage disequilibrium
LDSC: LD score regression
MAF: Minor allele frequency
METSIM: Metabolic syndrome in men cohort
MOSS: Mexican obesity study cohort
NC: Negative control
OE: Overexpress
PPARA: Peroxisome proliferator activated receptor alpha
PPARG: Peroxisome proliferator activated receptor gamma
PCA: Principal component analysis
PC: Principal component
PIP: Posterior inclusion probability
PRS: Polygenic risk score
qPCR: Quantitative PCR
RSB: Resuspension buffer
RNA-seq: RNA sequencing
SHBG: Sex hormone binding globulin
siRNA: Small interfering RNA
SNP: Single-nucleotide polymorphisms
snRNA-seq: Single-nucleus RNA sequencing
T2D: Type 2 diabetes
TBX15: T-box Transcription Factor 15
TF: Transcription factor
TSS: Transcription start site
TWAS: Transcriptome-wide association study
UKB: UK Biobank
UMI: Unique molecular identifiers
UTR: Untranslated region
WC: Waist circumference
WGCNA: Weighted gene co-expression network analysis
WHR: Waist-hip ratio
WHRadjBMI: Waist-hip ratio adjusted for body mass index
ZNF3: Zinc finger protein 3
