# Correction to: Identification of TBX15 as an adipose master trans regulator of abdominal obesity genes

**DOI:** 10.1186/s13073-021-00954-3

**Published:** 2021-08-30

**Authors:** David Z. Pan, Zong Miao, Caroline Comenho, Sandhya Rajkumar, Amogha Koka, Seung Hyuk T. Lee, Marcus Alvarez, Dorota Kaminska, Arthur Ko, Janet S. Sinsheimer, Karen L. Mohlke, Nicholas Mancuso, Linda Liliana Muñoz-Hernandez, Miguel Herrera-Hernandez, Maria Teresa Tusié-Luna, Carlos Aguilar-Salinas, Kirsi H. Pietiläinen, Jussi Pihlajamäki, Markku Laakso, Kristina M. Garske, Päivi Pajukanta

**Affiliations:** 1 Department of Human Genetics, David Geffen School of Medicine at UCLA, Los Angeles, USA; 2 Bioinformatics Interdepartmental Program, UCLA, Los Angeles, USA; 3 Computational and Systems Biology Interdepartmental Program, UCLA, Los Angeles, USA; 4 Institute of Public Health and Clinical Nutrition, University of Eastern Finland, Kuopio, Finland; 5 Department of Medicine, David Geffen School of Medicine at UCLA, Los Angeles, USA; 6 Department of Computational Medicine, David Geffen School of Medicine at UCLA, Los Angeles, USA; 7 Department of Genetics, University of North Carolina at Chapel Hill, Chapel Hill, North Carolina USA; 8 Center for Genetic Epidemiology, Department of Preventative Medicine, Keck School of Medicine, University of Southern California, Los Angeles, USA; 9 Tecnologico de Monterrey, Escuela de Medicina y Ciencias de la Salud, Ave. Morones Prieto 3000, Monterrey, N.L. México 64710; 10 Unidad de Investigación de Enfermedades Metabólicas, Instituto Nacional de Ciencias Médicas y Nutrición Salvador Zubirán, Mexico City, Mexico; 11 Departamento de Endocrinología y Metabolismo del Instituto Nacional de Ciencias Médicas y Nutrición Salvador Zubirán, Mexico City, Mexico; 12 Departamento de Cirugía, Instituto Nacional de Ciencias Médicas y Nutrición, Mexico City, Mexico; 13 Unidad de Biología Molecular y Medicina Genómica, Instituto de Investigaciones Biomédicas UNAM/ Instituto Nacional de Ciencias Médicas y Nutrición Salvador Zubirán, Mexico City, Mexico; 14 Obesity Research Unit, Research Program for Clinical and Molecular Metabolism, Faculty of Medicine, University of Helsinki, Helsinki, Finland; 15 Obesity Center, Endocrinology, Abdominal Center, Helsinki University Central Hospital and University of Helsinki, Helsinki, Finland; 16 Department of Medicine, Endocrinology and Clinical Nutrition, Kuopio University Hospital, Kuopio, Finland; 17 Department of Medicine, University of Eastern Finland and Kuopio University Hospital, Kuopio, Finland; 18 Institute for Precision Health at UCLA, Los Angeles, USA

**Correction to: Genome Med 13, 123 (2021)**

**https://doi.org/10.1186/s13073-021-00939-2**

It was highlighted that in the original article [1] the UK Biobank Resource Application Number was incorrect (3934). The correct UK Biobank Resource Application Number is 33934. The authors also wished to add this sentence to their Acknowledgments section: “This research was conducted using the UK Biobank Resource under application 33934.” The original article has been updated.
