# Supplementary material for: Identification of TBX15 as an adipose master trans regulator of abdominal obesity genes
Source: Genome Med. 2021 Aug 2;13:123. doi: 10.1186/s13073-021-00939-2 (PMC8327600; doi:10.1186/s13073-021-00939-2)
Supplement: Supplementary file 1 — Additional file 1. Supplementary figures (Fig. S1-S4) [file 13073_2021_939_MOESM1_ESM.pdf]

Identification of *TBX15* as an adipose master *trans* regulator of abdominal obesity genes

David Z. Pan, Zong Miao, Caroline Comenho, Sandhya Rajkumar, Amogha Koka, Seung Hyuk T. Lee, Marcus Alvarez, Dorota Kaminska, Arthur Ko, Janet S. Sinsheimer, Karen L. Mohlke, Nicholas Mancuso, Linda Liliana Muñoz-Hernandez, Miguel Herrera-Hernandez, Maria Teresa Tusié-Luna, Carlos Aguilar-Salinas, Kirsi Pietiläinen, Jussi Pihlajamäki, Markku Laakso. Kristina M. Garske, Päivi Pajukanta

Supplementary Figures

Fig S1. Summary of WGCNA parameters with regards to soft-thresholding power.

Fig S2. Schematic overview of the study design.

Fig S3. snRNA-seq (n=15) identifies multiple cell-types expressing TBX15.

Fig S4. WGCNA network preservation using GTEx visceral adipose (n=277) RNA-seq data.

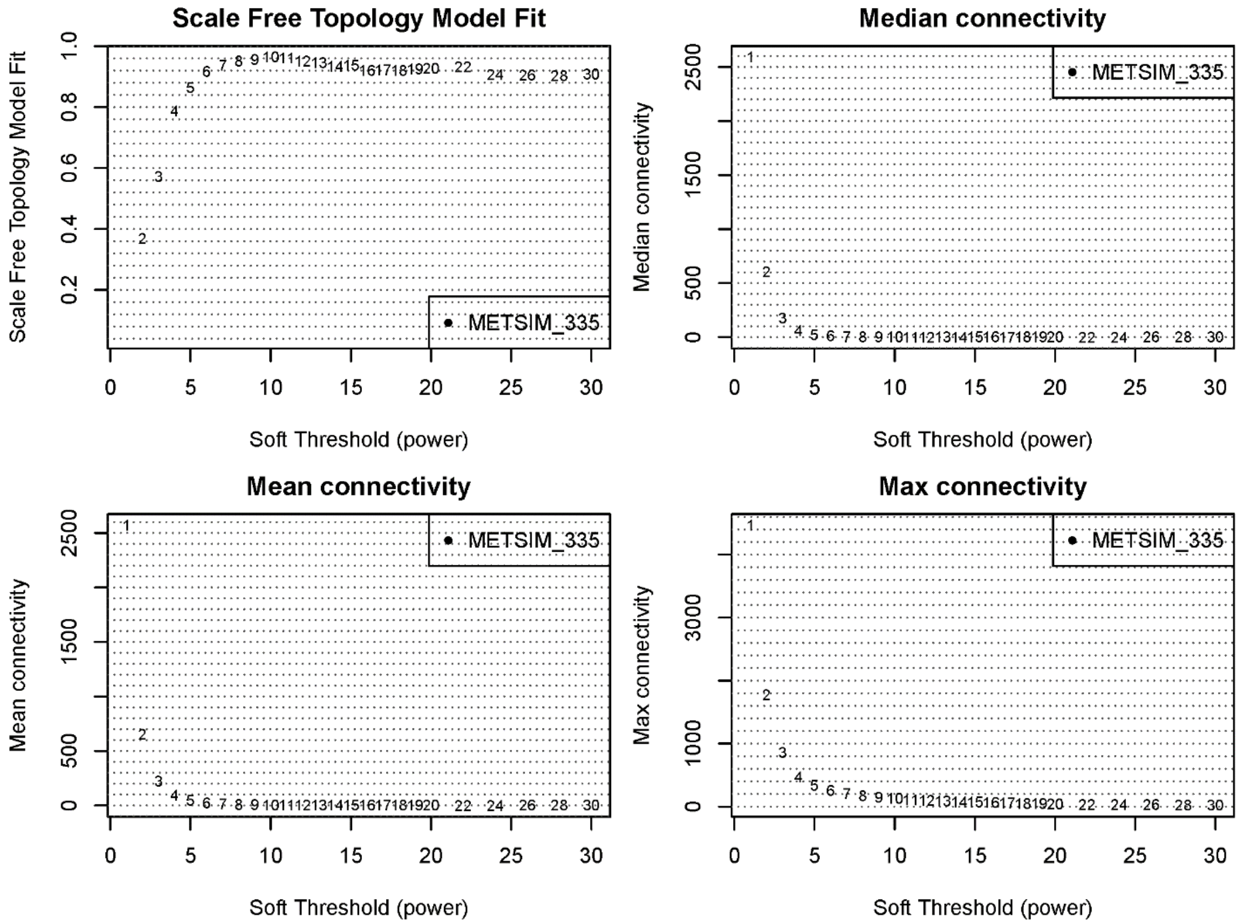

Fig. S1. Summary of WGCNA[39] parameters with regards to soft-thresholding power. Numbers and x-axis of plots indicate soft-thresholding power value. Y-axis indicates parameter used to determine parameters used to determine soft-thresholding value where scale-free topography approximation is achieved. Lowest power, 10, that satisfies scale-free topography was chosen to preserve highest amount of connectivity among genes.

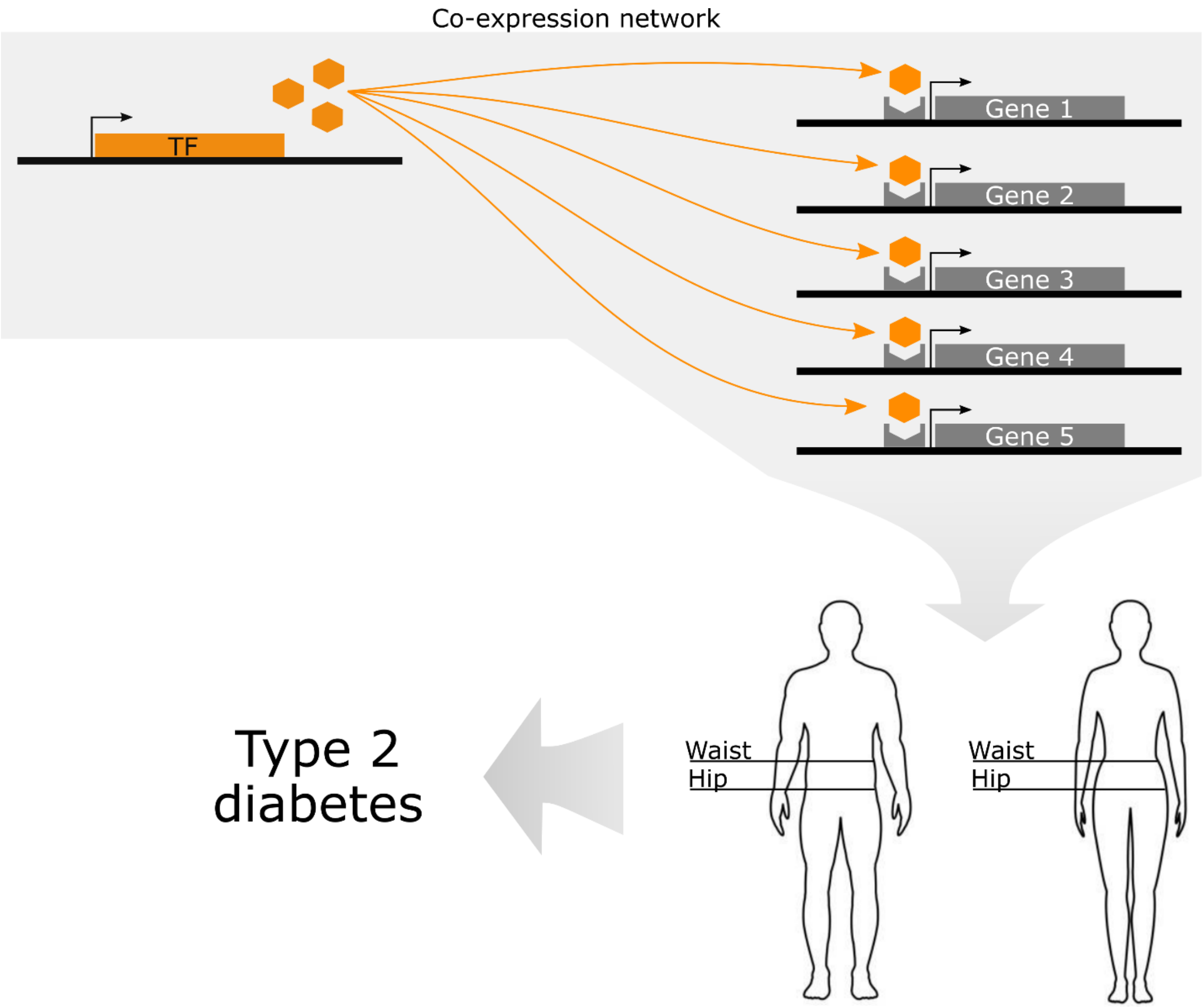

Fig. S2. Schematic overview of the study design. Illustrative schematic overview of the current study design, showing a TF, i.e. *TBX15*, controlling a co-expression network that ultimately affects WHRadjBMI and clinical metabolic outcome, T2D, in a sex-dependent manner.

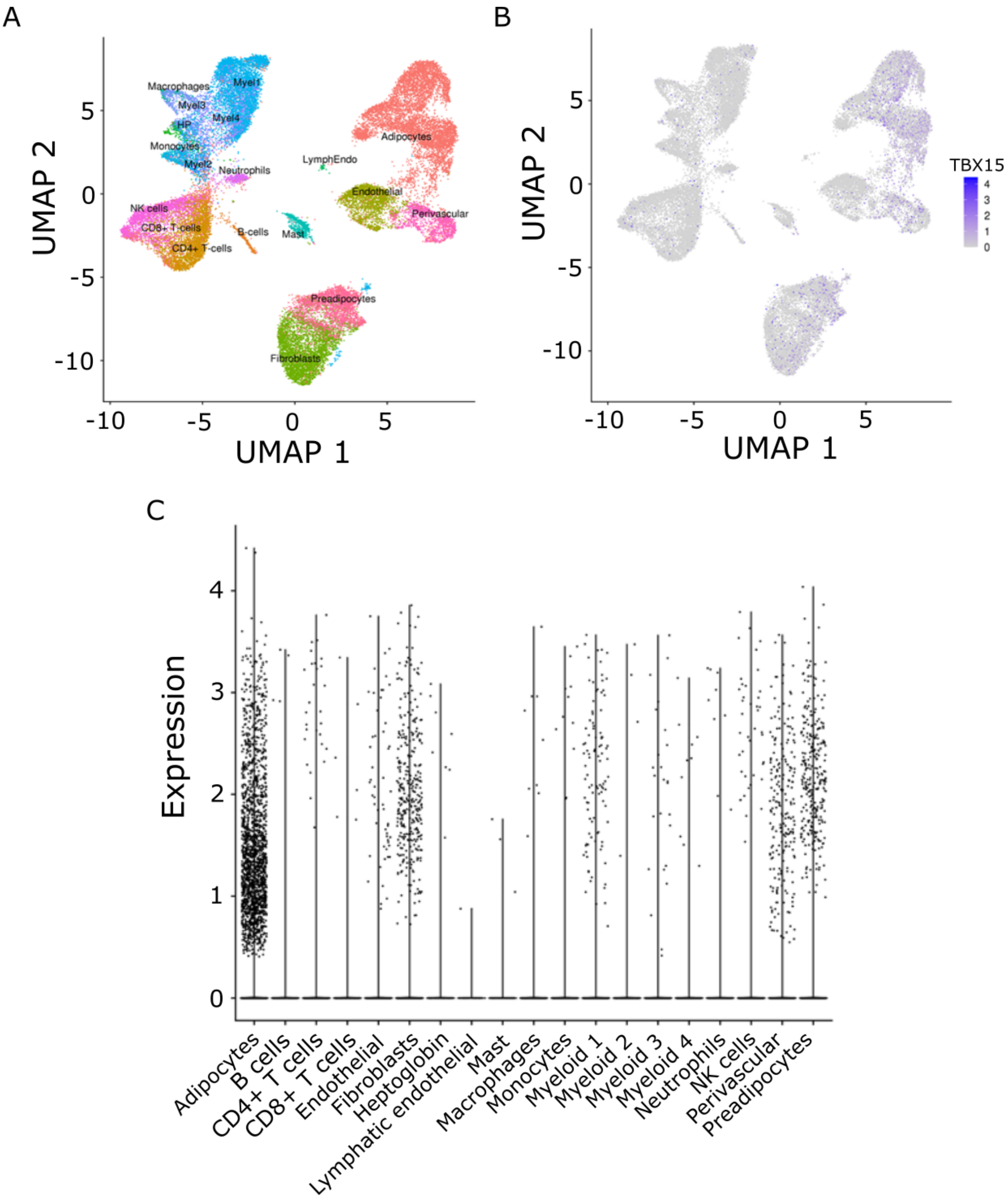

Fig. S3. snRNA-seq (n=15)[33,43,44] identifies multiple cell-types expressing *TBX15*. a, UMAP representation of cell-type clusters from snRNA-seq. **b,** UMAP representation of cells and cell types expressing *TBX15*. Expression values are *TBX15* counts normalized by total counts per cell, multiplied by a scaling factor, and then log transformed. **c,** Violin plot of number of cells per cell type from snRNA-seq expressing *TBX15*.

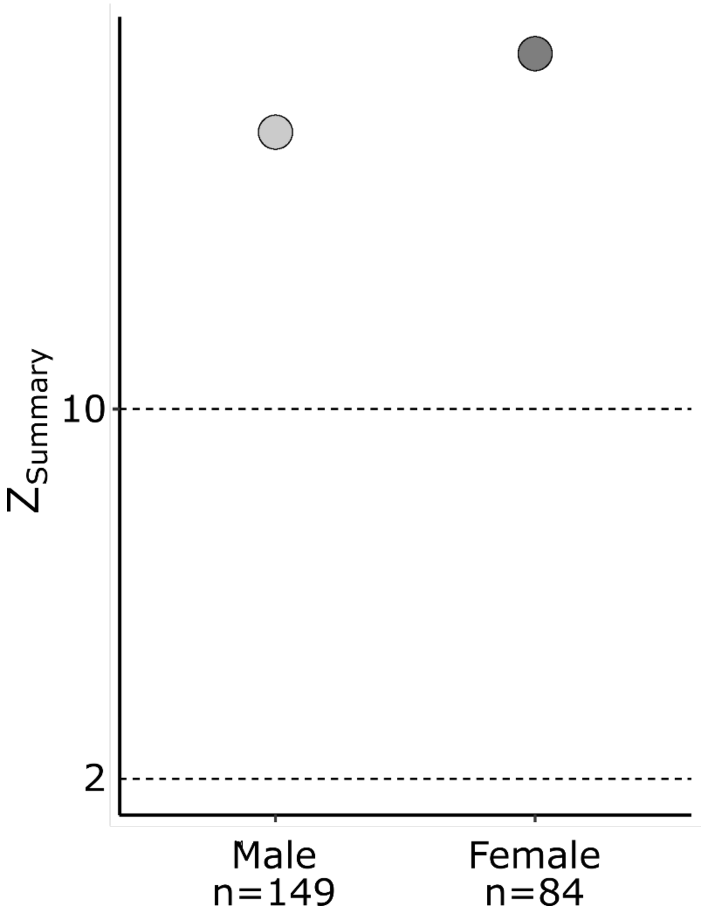

Fig S4. WGCNA[39] network preservation using GTEx[58] visceral adipose (n=277) RNA-seq data. WGCNA [39] network preservation results in males (n=149) and females (n=84) separately using GTEx visceral adipose (n=277) RNA-seq data. Thresholds for weak and strong network preservation $Z_{Summary}$ score indicated by dashed lines. A preservation $10>Z_{Summary}>2$ was considered as weakly to moderately preserved and a $Z_{Summary}>10$ as strongly preserved[38,40].
