# Supplementary material for: Identification of TBX15 as an adipose master trans regulator of abdominal obesity genes
Source: Genome Med. 2021 Aug 2;13:123. doi: 10.1186/s13073-021-00939-2 (PMC8327600; doi:10.1186/s13073-021-00939-2)
Supplement: Supplementary file 2 — Additional file 2. Supplementary tables (Table S1-S12) [file 13073_2021_939_MOESM2_ESM.pdf]

Identification of *TBX15* as an adipose master *trans* regulator of abdominal obesity genes

David Z. Pan, Zong Miao, Caroline Comenho, Sandhya Rajkumar, Amogha Koka, Seung Hyuk T. Lee, Marcus Alvarez, Dorota Kaminska, Arthur Ko, Janet S. Sinsheimer, Karen L. Mohlke, Nicholas Mancuso, Linda Liliana Muñoz-Hernandez, Miguel Herrera-Hernandez, Maria Teresa Tusié-Luna, Carlos Aguilar-Salinas, Kirsi Pietiläinen, Jussi Pihlajamäki, Markku Laakso. Kristina M. Garske, Päivi Pajukanta

Supplementary Tables

Table S1: Characteristics of the genes (n=347) in the WHRadjBMI co-expression network, as reported by WGCNA and ranked by network membership.

Table S2: Characteristics of the obesity GWAS genes in WHRadjBMI co-expression network, as reported by WGCNA and ranked by network membership.

Table S3: Cell-type marker genes from snRNA-seq grouped by cell type and ranked by Bonferroni adjusted p-value.

Table S4: Characteristics of the cell-type marker genes in WHRadjBMI co-expression network, as reported by WGCNA and ranked by network membership.

Table S5: KEGG pathway enrichment results (passing FDR<0.05) from WebGestalt for the WHRadjBMI co-expression network genes.

Table S6: Gene Ontology cellular component enrichment results (passing FDR<0.05) from WebGestalt for the WHRadjBMI co-expression network genes.

Table S7: Stratified LD Score Regression results for WHRadjBMI, T2D, and BMI using the *cis* variants (+/-500kb from the ends of the gene) of the WHRadjBMI co-expression network genes.

Table S8: Characteristics of the TFs in WHRadjBMI co-expression network, as reported by WGCNA and ranked by network membership.

Table S9: Significant TWAS heritability estimates (p<0.01) for the TFs in the WHRadjBMI co-expression network.

Table S10: TWAS p-values and Z-scores for associations of TFs (with significant TWAS heritability (p<0.01)) with WHRadjBMI.

Table S11: Significantly differentially expressed genes (FDR<0.05) in the WHRadjBMI co-expression network between the baseline and follow-up time points in the KOBS cohort ranked by p-value.

Table S12: Significantly differentially expressed genes (FDR<0.05) in the WHRadjBMI co-expression network in the *TBX15* knockdown experiment ranked by p-value.

Table S1: Characteristics of the genes (n=347) in the WHRadjBMI co-expression network, as reported by WGCNA[39] and ranked by network membership.

Gene Name	Chr*:start-end (hg19)	Network membership†	WHRadjBMI correlation‡	Fasting insulin correlation‡
HADH	chr4:108910870-108956331	0.929	-0.253	-0.439
ETFA	chr15:76507696-76603813	0.898	-0.244	-0.454
ALDH6A1	chr14:74523553-74551196	0.895	-0.192	-0.399
HIBADH	chr7:27565061-27702614	0.874	-0.208	-0.367
UQCRC2	chr16:21963981-21994981	0.872	-0.246	-0.357
ACVR1C	chr2:158383279-158485517	0.871	-0.185	-0.402
GPD1L	chr3:32147181-32210205	0.866	-0.237	-0.481
BNIP3	chr10:133781578-133795435	0.855	-0.228	-0.361
MARC2	chr1:220921567-220958150	0.851	-0.192	-0.357
MUT	chr6:49398073-49430904	0.850	-0.166	-0.345
PCCA	chr13:100741269-101182686	0.846	-0.201	-0.356
CCDC50	chr3:191046866-191116459	0.845	-0.194	-0.373
ACSS3	chr12:81331594-81650533	0.840	-0.171	-0.405
DLST	chr14:75348594-75370448	0.839	-0.189	-0.353
PHF13	chr1:6673745-6684093	0.837	-0.218	-0.363
NIPSNAP3B	chr9:107526438-107539738	0.834	-0.217	-0.470
ANO6	chr12:45609770-45834187	0.833	-0.215	-0.366
C1orf43	chr1:154179182-154193104	0.833	-0.210	-0.347
VDAC2	chr10:76969912-76991206	0.830	-0.231	-0.375
NDUFB5	chr3:179322478-179345435	0.829	-0.225	-0.358
AUH	chr9:93976097-94124195	0.825	-0.176	-0.307
ACAT1	chr11:107992243-108018503	0.824	-0.149	-0.351
NAALAD2	chr11:89864683-89926062	0.824	-0.240	-0.408
PEX19	chr1:160246602-160256138	0.824	-0.167	-0.319
PRDX6	chr1:173446405-173457946	0.823	-0.261	-0.395
GBAS	chr7:56019486-56067874	0.820	-0.170	-0.283
HRSP12	chr8:99114572-99129469	0.815	-0.226	-0.376
ADH1B	chr4:100226121-100242558	0.814	-0.160	-0.441
CTH	chr1:70876901-70905534	0.811	-0.174	-0.374
MOCS1	chr6:39867354-39902290	0.809	-0.184	-0.451
SUCLG2	chr3:67410884-67705038	0.809	-0.203	-0.348
CSNK2A2	chr16:58191811-58231824	0.804	-0.256	-0.347
MRPL45	chr17:36452989-36479101	0.804	-0.208	-0.304
PHYH	chr10:13319796-13344412	0.804	-0.188	-0.323
DNAJC19	chr3:180701497-180707562	0.803	-0.131	-0.357
VEGFA	chr6:43737921-43754224	0.801	-0.155	-0.386
MRPL32	chr7:42971799-42988557	0.800	-0.149	-0.348
HSDL2	chr9:115142217-115234690	0.799	-0.167	-0.309
CYB5A	chr18:71920530-71959251	0.798	-0.211	-0.381
TCEB3	chr1:24069645-24088549	0.797	-0.226	-0.332

EIF4EBP2	chr10:72164135-72188374	0.795	-0.160	-0.371
HLF	chr17:53342373-53402426	0.794	-0.195	-0.381
SLC19A2	chr1:169433147-169455241	0.791	-0.184	-0.338
BFAR	chr16:14726672-14763093	0.789	-0.218	-0.336
PDK2	chr17:48172101-48189516	0.787	-0.165	-0.359
RP11-61A14.3	chr16:66923072-66924996	0.787	-0.132	-0.330
MRPS36	chr5:68513587-68525956	0.786	-0.210	-0.331
TWIST1	chr7:19060614-19157295	0.784	-0.160	-0.392
GKAP1	chr9:86354336-86444431	0.783	-0.182	-0.333
PFKFB3	chr10:6186881-6277495	0.783	-0.184	-0.390
TMEM132C	chr12:128751948-129192460	0.783	-0.118	-0.377
ACADM	chr1:76190036-76253260	0.780	-0.195	-0.360
LETMD1	chr12:51441745-51454207	0.779	-0.147	-0.350
MED9	chr17:17380300-17396540	0.779	-0.233	-0.347
GINS3	chr16:58328984-58440048	0.778	-0.231	-0.426
KLF15	chr3:126061478-126076285	0.775	-0.215	-0.300
MRPL10	chr17:45900638-45908900	0.775	-0.190	-0.325
RBPMS-AS1	chr8:30239635-30242917	0.775	-0.210	-0.379
UNG	chr12:109535379-109548797	0.775	-0.243	-0.310
CRLS1	chr20:5986736-6020699	0.774	-0.104	-0.314
TMLHE	chrX:154719776-154899605	0.774	-0.156	-0.288
LRPPRC	chr2:44113647-44223144	0.771	-0.177	-0.280
SLC27A2	chr15:50474393-50528592	0.771	-0.248	-0.407
TMEM100	chr17:53796988-53809482	0.770	-0.229	-0.336
PAIP2B	chr2:71409869-71454213	0.769	-0.237	-0.379
ORMDL3	chr17:38077294-38083854	0.768	-0.202	-0.401
OSBPL1A	chr18:21742008-21977844	0.768	-0.209	-0.343
AASS	chr7:121715701-121784334	0.767	-0.138	-0.393
ATPAF1	chr1:47098409-47139539	0.767	-0.109	-0.283
ASH2L	chr8:37962760-38001594	0.764	-0.104	-0.297
PPARA	chr22:46546424-46639653	0.762	-0.160	-0.362
TXLNG	chrX:16804550-16862642	0.762	-0.160	-0.304
GHR	chr5:42423879-42721979	0.761	-0.100	-0.364
TTLL7	chr1:84330711-84464833	0.760	-0.194	-0.360
GMCL1	chr2:70056774-70108528	0.759	-0.208	-0.307
PER3	chr1:7844380-7905237	0.759	-0.186	-0.332
RMND1	chr6:151725989-151773259	0.759	-0.226	-0.289
PJA1	chrX:68380694-68385636	0.755	-0.137	-0.234
ANKRD53	chr2:71205510-71212626	0.754	-0.190	-0.411
NMNAT3	chr3:139279022-139396859	0.753	-0.113	-0.261
GPR146	chr7:1084212-1098897	0.751	-0.182	-0.343
AC003986.6	chr7:19152097-19153894	0.750	-0.193	-0.327
GLUL	chr1:182350839-182361341	0.750	-0.206	-0.371
FAM89A	chr1:231154704-231175992	0.749	-0.153	-0.391
APMAP	chr20:24943561-24973615	0.748	-0.245	-0.310

DHTKD1	chr10:12110971-12165224	0.747	-0.215	-0.250
IMMP2L	chr7:110303110-111202573	0.747	-0.216	-0.302
FDFT1	chr8:11653082-11696818	0.746	-0.160	-0.374
DBT	chr1:100652475-100715390	0.745	-0.127	-0.293
USP30	chr12:109460894-109525831	0.743	-0.159	-0.229
ABHD5	chr3:43731605-43775863	0.741	-0.161	-0.351
SLC35G2	chr3:136537489-136574734	0.741	-0.191	-0.353
TGDS	chr13:95226308-95248511	0.741	-0.174	-0.304
EPB41L4B	chr9:111934255-112083244	0.740	-0.176	-0.353
SDHD	chr11:111957497-111990353	0.740	-0.185	-0.307
GPHN	chr14:66974125-67648520	0.739	-0.204	-0.359
PRKAR2B	chr7:106685094-106802256	0.739	-0.142	-0.372
LACTB2	chr8:71547553-71581409	0.737	-0.229	-0.295
YPEL5	chr2:30369807-30383399	0.737	-0.224	-0.323
MCCC2	chr5:70883115-70954531	0.736	-0.170	-0.216
GRPEL1	chr4:7060633-7069924	0.735	-0.256	-0.356
DLD	chr7:107531415-107572175	0.734	-0.172	-0.281
FBXO27	chr19:39481354-39523425	0.734	-0.129	-0.335
TBX15	chr1:119425669-119532179	0.733	-0.155	-0.311
HSPD1	chr2:198351305-198381461	0.732	-0.203	-0.322
PCBD1	chr10:72642037-72648541	0.732	-0.234	-0.293
PMM1	chr22:41972898-41985894	0.730	-0.208	-0.348
MPDZ	chr9:13105703-13279589	0.729	-0.133	-0.296
SLC41A1	chr1:205758221-205782876	0.729	-0.206	-0.312
STOX1	chr10:70587298-70655188	0.729	-0.216	-0.365
DAPK2	chr15:64199235-64364232	0.728	-0.227	-0.375
MLYCD	chr16:83932731-83949787	0.728	-0.210	-0.293
TARSL2	chr15:102193801-102264807	0.728	-0.205	-0.335
BCKDHB	chr6:80816364-81055987	0.727	-0.145	-0.256
FAM120AOS	chr9:96208776-96215874	0.727	-0.120	-0.292
MLX	chr17:40719086-40725257	0.726	-0.266	-0.302
PMPCB	chr7:102937869-102969958	0.726	-0.140	-0.254
RP11-363E7.4	chr9:19453207-19455171	0.726	-0.109	-0.399
MTHFD1	chr14:64854749-64926722	0.725	-0.215	-0.297
TBC1D20	chr20:416124-443197	0.725	-0.201	-0.357
FAM13A	chr4:89647106-90032549	0.724	-0.140	-0.272
PHLPP1	chr18:60382672-60647666	0.724	-0.116	-0.386
ELP2	chr18:33709407-33757909	0.723	-0.210	-0.350
MCCC1	chr3:182733006-182833863	0.723	-0.135	-0.265
AC108142.1	chr4:182795591-183066402	0.722	-0.181	-0.361
HDDC2	chr6:125541108-125623282	0.721	-0.155	-0.284
MRPL44	chr2:224822121-224832431	0.721	-0.208	-0.280
SRSF4	chr1:29474255-29508499	0.719	-0.175	-0.260
SRP68	chr17:74035184-74068734	0.718	-0.201	-0.323
CCNH	chr5:86687311-86708836	0.716	-0.152	-0.304

PDP2	chr16:66912492-66929657	0.716	-0.098	-0.268
RCL1	chr9:4792869-4885917	0.716	-0.163	-0.299
SLC25A21-AS1	chr14:37641093-37643016	0.716	-0.145	-0.317
THYN1	chr11:134118173-134123264	0.716	-0.179	-0.301
RASL10B	chr17:34058668-34070540	0.715	-0.197	-0.419
MRPL39	chr21:26957968-26979829	0.714	-0.165	-0.282
SIX4	chr14:61176246-61191066	0.712	-0.204	-0.270
MARC1	chr1:220960101-220987735	0.711	-0.224	-0.297
ETFDH	chr4:159593277-159630775	0.710	-0.069	-0.286
GPN3	chr12:110890289-110907073	0.709	-0.166	-0.296
HOMEZ	chr14:23741666-23768656	0.708	-0.272	-0.316
MRPS22	chr3:138724648-139076065	0.707	-0.114	-0.331
BTG3	chr21:18965971-18985265	0.705	-0.136	-0.340
C11orf1	chr11:111749659-111756699	0.705	-0.188	-0.253
IARS2	chr1:220267444-220321380	0.705	-0.159	-0.241
HADHB	chr2:26466038-26513336	0.702	-0.124	-0.252
MRPS27	chr5:71515236-71616473	0.701	-0.110	-0.226
TRHDE-AS1	chr12:72647288-72668687	0.701	-0.135	-0.291
BMP3	chr4:81952119-81978685	0.700	-0.170	-0.425
SLC4A4	chr4:72053003-72437804	0.700	-0.187	-0.324
TTC36	chr11:118398187-118401912	0.700	-0.202	-0.403
ANAPC16	chr10:73975787-73995618	0.699	-0.221	-0.324
SLC19A3	chr2:228549926-228582728	0.698	-0.096	-0.413
RHOT1	chr17:30469473-30580393	0.697	-0.239	-0.287
RP11-61A14.2	chr16:66921918-66922834	0.697	-0.131	-0.283
SDHB	chr1:17345217-17380665	0.697	-0.163	-0.285
LRRC41	chr1:46726868-46769280	0.696	-0.151	-0.285
CECR2	chr22:17840837-18037850	0.695	-0.204	-0.442
LSM6	chr4:147096837-147121152	0.695	-0.201	-0.334
UTS2B	chr3:190984957-191048325	0.694	-0.246	-0.353
GYG2P1	chrY:14475147-14532255	0.691	-0.199	-0.347
NFU1	chr2:69622882-69664760	0.691	-0.173	-0.309
ZNF16	chr8:146155744-146176274	0.691	-0.177	-0.279
C12orf39	chr12:21679241-21690311	0.690	-0.252	-0.449
CFL2	chr14:35179593-35184029	0.690	-0.060	-0.283
PPP3R1	chr2:68405989-68483369	0.690	-0.207	-0.323
ABCB7	chrX:74273115-74376567	0.689	-0.154	-0.251
AKAP1	chr17:55162453-55198710	0.688	-0.138	-0.324
SNRNP27	chr2:70120692-70132707	0.688	-0.141	-0.248
TM7SF2	chr11:64879317-64883856	0.688	-0.149	-0.329
MAP3K5	chr6:136878185-137113656	0.686	-0.228	-0.359
ABHD15	chr17:27887565-27894155	0.684	-0.162	-0.283
AQP7	chr9:33384765-33402643	0.683	-0.153	-0.258
ISCA1	chr9:88879461-88897676	0.681	-0.136	-0.289
ADCK3	chr1:227085237-227175246	0.679	-0.165	-0.350

DNAJA3	chr16:4475806-4506776	0.679	-0.167	-0.267
L2HGDH	chr14:50704281-50779266	0.679	-0.182	-0.292
RP11-789C1.1	chr4:171195070-171204230	0.679	-0.169	-0.386
STRADB	chr2:202252581-202345569	0.679	-0.238	-0.290
ZFYVE21	chr14:104182067-104200005	0.679	-0.120	-0.267
GRSF1	chr4:71681499-71705662	0.678	-0.247	-0.239
RGS17	chr6:153325594-153452384	0.678	-0.167	-0.374
ACADSB	chr10:124768495-124817827	0.677	-0.085	-0.206
PTPN3	chr9:112137746-112260590	0.676	-0.163	-0.369
RDH10	chr8:74206847-74237516	0.676	-0.064	-0.260
ANG	chr14:21152336-21167130	0.674	-0.177	-0.386
C2orf47	chr2:200820040-200873263	0.673	-0.114	-0.247
WDR20	chr14:102605840-102691184	0.672	-0.094	-0.223
CIDEA	chr18:12254318-12277594	0.671	-0.161	-0.392
SETD9	chr5:56205087-56221359	0.670	-0.026	-0.315
HADHA	chr2:26413504-26467594	0.669	-0.177	-0.353
PPARG	chr3:12328867-12475855	0.666	-0.257	-0.308
RP11-387H17.4	chr17:38083995-38095854	0.666	-0.209	-0.405
AFG3L2	chr18:12328943-12377313	0.665	-0.144	-0.218
PDHX	chr11:34937376-35042138	0.665	-0.062	-0.260
GGCT	chr7:30536237-30591095	0.661	-0.189	-0.319
GSDMB	chr17:38060848-38076107	0.660	-0.258	-0.396
ISOC1	chr5:128430444-128449721	0.659	-0.161	-0.256
EIF1	chr17:39845137-39848920	0.658	-0.219	-0.351
SULF1	chr8:70378859-70573150	0.657	-0.185	-0.300
EYS	chr6:64429876-66417118	0.656	-0.140	-0.224
GTF2E2	chr8:30435835-30515768	0.656	-0.165	-0.292
SLC25A27	chr6:46620678-46645930	0.656	-0.123	-0.228
OXCT1	chr5:41730167-41870621	0.655	-0.122	-0.240
XPNPEP3	chr22:41253081-41363838	0.655	-0.118	-0.277
MKNK2	chr19:2037470-2051243	0.654	-0.220	-0.324
SIK2	chr11:111473115-111601577	0.653	-0.038	-0.252
CHCHD3	chr7:132469629-132766848	0.652	-0.194	-0.265
LONRF1	chr8:12579403-12613582	0.652	-0.208	-0.301
ZDHHC4	chr7:6617065-6629005	0.651	-0.139	-0.250
RPAIN	chr17:5322961-5336196	0.650	-0.197	-0.243
ARPC1A	chr7:98923521-98985787	0.648	-0.111	-0.292
CALCRL	chr2:188207856-188313187	0.648	-0.083	-0.286
HMGN3	chr6:79910962-79944406	0.648	-0.146	-0.263
TMEM220	chr17:10602332-10633633	0.647	-0.135	-0.291
BAG4	chr8:38034051-38070819	0.645	-0.116	-0.302
NDFIP2	chr13:80055287-80130210	0.645	-0.089	-0.320
TMEM52	chr1:1849029-1850712	0.644	-0.230	-0.356
GPR180	chr13:95254157-95286899	0.642	-0.186	-0.267
ACO1	chr9:32384618-32454767	0.638	-0.175	-0.250

PPP2R5A	chr1:212458879-212535200	0.638	-0.145	-0.303
RP11-61I13.3	chr6:39849580-39867847	0.638	-0.172	-0.357
MRPS18A	chr6:43639040-43655528	0.636	-0.116	-0.269
VWA8	chr13:42140973-42535256	0.635	-0.146	-0.180
URAHP	chr16:90106169-90114181	0.634	-0.188	-0.325
USP13	chr3:179370543-179507189	0.634	-0.202	-0.307
ACAD8	chr11:134123389-134135749	0.633	-0.130	-0.219
MRPS35	chr12:27863706-27909228	0.633	-0.151	-0.288
ZNF3	chr7:99661656-99680171	0.633	-0.127	-0.296
KCNIP2	chr10:103585731-103603677	0.631	-0.096	-0.271
STXBP1	chr9:130374544-130457460	0.631	-0.089	-0.338
CA3	chr8:86285665-86361269	0.630	-0.233	-0.236
EIF4EBP1	chr8:37887859-37917883	0.630	-0.191	-0.340
NRIP1	chr21:16333556-16437321	0.628	-0.111	-0.287
DNAH9	chr17:11501748-11873065	0.626	-0.093	-0.285
RP11-474O21.5	chr1:12678906-12679250	0.626	-0.171	-0.334
FRMD1	chr6:168456425-168482237	0.625	-0.198	-0.341
EIF1AY	chrY:22737611-22755040	0.622	-0.202	-0.312
KTN1-AS1	chr14:55965996-56046828	0.620	-0.149	-0.274
NDUFS1	chr2:206979541-207024327	0.620	-0.117	-0.209
ADRBK2	chr22:25960816-26125261	0.619	-0.168	-0.249
FHOD3	chr18:33877677-34360018	0.619	-0.231	-0.301
MRPS9	chr2:105654441-105716418	0.619	-0.253	-0.285
UQCC1	chr20:33890369-33999944	0.619	-0.139	-0.241
MPC1	chr6:166778407-166796486	0.618	-0.102	-0.323
CPT2	chr1:53662101-53679869	0.617	-0.118	-0.168
GPATCH11	chr2:37311594-37326387	0.617	-0.119	-0.197
VBP1	chrX:154425284-154468098	0.617	-0.184	-0.294
LRIG1	chr3:66429221-66551687	0.615	-0.117	-0.262
PAXIP1-AS1	chr7:154795158-154797413	0.613	-0.097	-0.221
PRDX3	chr10:120927215-120938345	0.612	-0.151	-0.206
TMEM25	chr11:118401756-118417995	0.612	-0.157	-0.417
ADH1A	chr4:100197524-100212185	0.609	-0.172	-0.344
AK4	chr1:65613232-65697828	0.609	-0.193	-0.283
PHGDH	chr1:120202421-120286838	0.609	-0.225	-0.250
TOMM70A	chr3:100082275-100120242	0.608	-0.124	-0.200
NDUFA5	chr7:123177051-123198309	0.606	-0.104	-0.293
RASSF6	chr4:74437267-74486348	0.603	-0.231	-0.377
IFT46	chr11:118415243-118443685	0.602	-0.128	-0.236
SLC16A7	chr12:59989848-60176395	0.602	-0.185	-0.267
LRRC47	chr1:3696784-3713068	0.601	-0.141	-0.319
RAI2	chrX:17818169-17879457	0.601	-0.060	-0.333
RP11-182I10.3	chr1:65437908-65468159	0.600	-0.050	-0.184
NEDD4L	chr18:55711599-56068772	0.597	-0.170	-0.318
SCO1	chr17:10583654-10601692	0.596	-0.116	-0.247

C17orf53	chr17:42219274-42239844	0.595	-0.184	-0.248
TP73-AS1	chr1:3652548-3663900	0.595	-0.061	-0.262
GLIS1	chr1:53971910-54199877	0.593	-0.208	-0.289
EMC3	chr3:10004221-10052800	0.592	-0.034	-0.304
MAN2A2	chr15:91445448-91465814	0.591	-0.182	-0.278
RP1-266L20.2	chr6:170125187-170125950	0.591	-0.145	-0.336
ANKRD46	chr8:101521980-101572012	0.590	-0.072	-0.247
MRS2	chr6:24403153-24425810	0.589	-0.114	-0.179
NDRG4	chr16:58496750-58547532	0.589	-0.174	-0.368
MRPL35	chr2:86426478-86440917	0.586	-0.124	-0.236
NKIRAS1	chr3:23933151-23988082	0.584	-0.119	-0.188
CENPV	chr17:16245848-16256970	0.582	-0.198	-0.300
TUSC1	chr9:25676396-25678856	0.582	-0.123	-0.316
C1orf50	chr1:43232940-43263968	0.581	-0.199	-0.289
PDHB	chr3:58413357-58419584	0.581	-0.129	-0.256
GFPT1	chr2:69546905-69614382	0.579	-0.096	-0.281
SYAP1	chrX:16737755-16783459	0.579	-0.093	-0.224
RP11-689P11.2	chr4:8483997-8514337	0.576	-0.120	-0.225
VPS72	chr1:151142463-151167797	0.576	-0.178	-0.294
ACO2	chr22:41865129-41924993	0.575	-0.113	-0.203
IRX1	chr5:3596168-3601517	0.574	-0.173	-0.263
ADAMTS9-AS2	chr3:64670585-64997143	0.572	-0.107	-0.284
GLYCTK	chr3:52321105-52329272	0.570	-0.202	-0.283
LONP2	chr16:48278207-48397033	0.567	-0.156	-0.193
SNX3	chr6:108532426-108582464	0.567	-0.091	-0.204
HDDC3	chr15:91474148-91475799	0.564	-0.194	-0.290
MET	chr7:116312444-116438440	0.562	-0.089	-0.310
RGS3	chr9:116207011-116360018	0.561	-0.236	-0.346
SLC43A1	chr11:57252007-57283259	0.561	-0.144	-0.170
CHKA	chr11:67820326-67888911	0.555	-0.214	-0.321
GHITM	chr10:85899196-85913001	0.555	-0.095	-0.214
GBE1	chr3:81538850-81811312	0.550	-0.052	-0.220
RTN3	chr11:63448918-63527363	0.544	-0.136	-0.225
TMEM230	chr20:5080486-5093749	0.540	-0.075	-0.218
ARSEP1	chrY:14460540-14468226	0.539	-0.100	-0.222
SIRT3	chr11:215458-236931	0.539	-0.084	-0.286
ADIPOQ	chr3:186560463-186576252	0.538	-0.071	-0.243
GABARAPL1	chr12:10365057-10375727	0.532	-0.039	-0.272
PRKAG2-AS1	chr7:151574127-151576299	0.532	-0.169	-0.261
ERCC8	chr5:60169658-60240900	0.531	-0.084	-0.228
TMEM42	chr3:44903361-44907162	0.523	-0.111	-0.198
ST6GALNAC6	chr9:130647600-130667687	0.517	-0.107	-0.372
SDHC	chr1:161284047-161332984	0.514	-0.059	-0.163
FBXO9	chr6:52916789-52965671	0.513	-0.119	-0.154
CDKN1C	chr11:2904443-2907111	0.503	-0.156	-0.268

FZD9	chr7:72848109-72850450	0.499	-0.112	-0.235
TSPAN3	chr15:77336359-77376326	0.498	-0.129	-0.136
FBXL5	chr4:15606162-15683302	0.495	-0.132	-0.163
SCOC	chr4:141178440-141306880	0.476	-0.021	-0.213
ATP5F1	chr1:111991486-112005395	0.474	-0.103	-0.159
HSPA9	chr5:137890571-137911133	0.426	-0.078	-0.150
IMMT	chr2:86371055-86422893	0.386	-0.057	-0.126
DSEL	chr18:65173819-65184217	-0.428	0.244	0.253
APBB1IP	chr10:26727132-26856732	-0.483	0.176	0.280
NEK6	chr9:127019885-127115586	-0.505	0.097	0.377
TSSC1	chr2:3192696-3381653	-0.506	0.108	0.259
GNG2	chr14:52292913-52446060	-0.531	0.201	0.334
CORO1C	chr12:109038885-109125372	-0.538	0.256	0.327
SPARC	chr5:151040657-151066726	-0.541	0.290	0.371
LBP	chr20:36974759-37005665	-0.552	0.132	0.384
CAPN1	chr11:64948037-64979477	-0.553	0.140	0.235
FAT2	chr5:150883654-150948505	-0.555	0.166	0.369
AP5S1	chr20:3801178-3805949	-0.576	0.274	0.291
KRT5	chr12:52908359-52914471	-0.576	0.233	0.389
TUBB2A	chr6:3153903-3157760	-0.587	0.208	0.303
NUDT1	chr7:2281857-2290781	-0.592	0.119	0.233
CA11	chr19:49141199-49149569	-0.600	0.169	0.249
TMEM189	chr20:48697663-48770335	-0.602	0.212	0.374
DPP3	chr11:66247484-66277130	-0.621	0.172	0.299
HPD	chr12:122277433-122301502	-0.626	0.220	0.480
PRAF2	chrX:48928813-48931730	-0.626	0.141	0.198
ACTN1	chr14:69340860-69446157	-0.632	0.211	0.379
TMSB10	chr2:85132749-85133795	-0.639	0.149	0.235
GNAI2	chr3:50263724-50296787	-0.640	0.155	0.223
CD248	chr11:66081958-66084515	-0.646	0.226	0.335
HOMER3	chr19:19040010-19052070	-0.647	0.193	0.265
TRPM2	chr21:45770046-45862964	-0.652	0.199	0.354
FLNA	chrX:153576892-153603006	-0.671	0.161	0.310
TMEM104	chr17:72772622-72835918	-0.673	0.165	0.360
ANKDD1A	chr15:65204101-65251042	-0.696	0.212	0.429
MSC	chr8:72753784-72756703	-0.725	0.287	0.361
C9orf16	chr9:130922539-130926207	-0.750	0.171	0.382

* Abbreviation for chromosome.

† Pearson correlation coefficient with network eigengene as reported by WGCNA[39].

‡ Pearson correlation coefficient with phenotype as reported by WGCNA[39].

Table S2: Characteristics of the obesity GWAS genes in WHRadjBMI co-expression network, as reported by WGCNA[39] and ranked by network membership.

Gene Name	GWAS trait*	Chr†:start-end (hg19)	Network membership‡	WHRadjBMI correlation§	Fasting insulin correlation§
PHF13	BMI	chr1:6673745-6684093	0.837	-0.218	-0.363
ADH1B	BMI	chr4:100226121-100242558	0.814	-0.160	-0.441
VEGFA	BMI WHR WHRadjBMI WCadjBMI	chr6:43737921-43754224	0.801	-0.155	-0.386
MRPL10	BMI	chr17:45900638-45908900	0.775	-0.190	-0.325
TTLL7	BMI	chr1:84330711-84464833	0.760	-0.194	-0.360
EPB41L4B	BMI WHR WHRadjBMI	chr9:111934255-112083244	0.740	-0.176	-0.353
DLD	WHRadjBMI	chr7:107531415-107572175	0.734	-0.172	-0.281
TBX15	BMI WHR WHRadjBMI WCadjBMI	chr1:119425669-119532179	0.733	-0.155	-0.311
BCKDHB	WHR WHRadjBMI WCadjBMI	chr6:80816364-81055987	0.727	-0.145	-0.256
FAM120AOS	BMI	chr9:96208776-96215874	0.727	-0.120	-0.292
FAM13A	WHR WHRadjBMI	chr4:89647106-90032549	0.724	-0.140	-0.272
MRPS22	BMI	chr3:138724648-139076065	0.707	-0.114	-0.331
LRRC41	BMI	chr1:46726868-46769280	0.696	-0.151	-0.285
CECR2	WHRadjBMI	chr22:17840837-18037850	0.695	-0.204	-0.442
ZFYVE21	BMI	chr14:104182067-104200005	0.679	-0.120	-0.267
RGS17	BMI WHR	chr6:153325594-153452384	0.678	-0.167	-0.374
SETD9	WHRadjBMI	chr5:56205087-56221359	0.670	-0.026	-0.315
PPARG	BMI WHR WHRadjBMI WC	chr3:12328867-12475855	0.666	-0.257	-0.308

EYS	BMI	chr6:64429876-66417118	0.656	-0.140	-0.224
ZDHHC4	BMI<br>WHRadjBMI	chr7:6617065-6629005	0.651	-0.139	-0.250
CALCRL	BMI<br>WHR<br>WHRadjBMI	chr2:188207856-188313187	0.648	-0.083	-0.286
TMEM52	BMI	chr1:1849029-1850712	0.644	-0.230	-0.356
MRPS18A	WHRadjBMI	chr6:43639040-43655528	0.636	-0.117	-0.269
NDUFS1	BMI	chr2:206979541-207024327	0.620	-0.117	-0.209
MRPS9	BMI	chr2:105654441-105716418	0.619	-0.253	-0.285
UQCC1	WHR<br>WHRadjBMI	chr20:33890369-33999944	0.619	-0.139	-0.241
LRIG1	BMI	chr3:66429221-66551687	0.615	-0.117	-0.262
ANKRD46	BMI	chr8:101521980-101572012	0.590	-0.072	-0.247
ADAMTS9-AS2	BMI<br>WHR<br>WHRadjBMI<br>WCadjBMI	chr3:64670585-64997143	0.572	-0.107	-0.284
MET	WHRadjBMI	chr7:116312444-116438440	0.562	-0.089	-0.310
GBE1	BMI<br>WC	chr3:81538850-81811312	0.550	-0.052	-0.220
ST6GALNAC6	BMI	chr9:130647600-130667687	0.517	-0.107	-0.372
GNAI2	BMI	chr3:50263724-50296787	-0.640	0.155	0.223
MSC	WHR<br>WHRadjBMI<br>WCadjBMI	chr8:72753784-72756703	-0.725	0.287	0.361
C9orf16	BMI	chr9:130922539-130926207	-0.750	0.171	0.382

* Abbreviations for GWAS traits: Body Mass Index (BMI), Waist-hip-ratio (WHR), Waist-Circumference (WC), Waist-hip-ratio adjusted for BMI (WHRadjBMI), Waist-Circumference adjusted for BMI (WCadjBMI).

† Abbreviation for chromosome.

‡ Pearson correlation coefficient with network eigengene as reported by WGCNA[39].

§ Pearson correlation coefficient with phenotype as reported by WGCNA[39].

Table S3: Cell-type marker genes from snRNA-seq [33,43,44] grouped by cell type and ranked by Bonferroni adjusted *p*-value.

Cell type	Gene name	Chr*:start-end (hg19)	$\log_2$ fold change†	Adjusted *p*-value‡
Adipocytes	CLSTN2	chr3:139654027-140296239	2.52	$<2.23 \times 10^{-308}$
	DIRC3	chr2:218148742-218621316	2.30	$<2.23 \times 10^{-308}$
	ITGA7	chr12:56078352-56109827	2.27	$<2.23 \times 10^{-308}$
	SORBS1	chr10:97071528-97321171	2.23	$<2.23 \times 10^{-308}$
	WDPCP	chr2:63348518-64054977	2.21	$<2.23 \times 10^{-308}$
	GPAM	chr10:113909624-113975135	2.16	$<2.23 \times 10^{-308}$
	HOOK2	chr19:12873817-12983554	2.12	$<2.23 \times 10^{-308}$
	TRHDE-AS1	chr12:72647288-72668687	2.08	$<2.23 \times 10^{-308}$
	AQP7	chr9:33384765-33402643	2.03	$<2.23 \times 10^{-308}$
	TNS1	chr2:218664512-218867718	1.99	$<2.23 \times 10^{-308}$
	DMD	chrX:31115794-33357558	1.97	$<2.23 \times 10^{-308}$
	AC004538.3	chr7:11221359-11559802	1.96	$<2.23 \times 10^{-308}$
	AC002066.1	chr7:115878314-116139519	1.96	$<2.23 \times 10^{-308}$
	PDE3B	chr11:14665269-14892350	1.95	$<2.23 \times 10^{-308}$
	GHR	chr5:42423879-42721979	1.95	$<2.23 \times 10^{-308}$
	ERBB4	chr2:212240446-213403565	1.91	$<2.23 \times 10^{-308}$
	GYG2	chrX:2746829-2800859	1.85	$<2.23 \times 10^{-308}$
	PTPRF	chr1:43990858-44089343	1.85	$<2.23 \times 10^{-308}$
	PNPLA2	chr11:818902-825573	1.85	$<2.23 \times 10^{-308}$
	TLN2	chr15:62682725-63136830	1.84	$<2.23 \times 10^{-308}$
	PCDH9	chr13:66876967-67804468	1.80	$<2.23 \times 10^{-308}$
	PLXNA4	chr7:131808091-132333447	1.76	$<2.23 \times 10^{-308}$
	PLIN4	chr19:4502204-4517716	1.74	$<2.23 \times 10^{-308}$
	LIMCH1	chr4:41361624-41702061	1.73	$<2.23 \times 10^{-308}$
	CTIF	chr18:46065417-46389588	1.69	$<2.23 \times 10^{-308}$
	PTGER3	chr1:71318036-71513491	1.69	$<2.23 \times 10^{-308}$
	SLC19A3	chr2:228549926-228582728	1.68	$<2.23 \times 10^{-308}$
	PDZRN3	chr3:73431584-73674091	1.65	$<2.23 \times 10^{-308}$
	MGST1	chr12:16500076-16762193	1.65	$<2.23 \times 10^{-308}$
	SIK2	chr11:111473115-111601577	1.64	$<2.23 \times 10^{-308}$
	TENM3	chr4:183065140-183724177	1.63	$<2.23 \times 10^{-308}$
	DGKI	chr7:137065783-137531838	1.62	$<2.23 \times 10^{-308}$
	EGFEM1P	chr3:167967310-168548387	1.62	$<2.23 \times 10^{-308}$
	PCLO	chr7:82383329-82792246	1.60	$<2.23 \times 10^{-308}$
	RP11-125B21.2	chr9:2422702-2641395	1.60	$<2.23 \times 10^{-308}$
	TEAD1	chr11:12695969-12966298	1.59	$<2.23 \times 10^{-308}$
	RP11-507B12.2	chr15:61592525-61927648	1.58	$<2.23 \times 10^{-308}$
	LIPE	chr19:42905659-42931578	1.58	$<2.23 \times 10^{-308}$
	ACSS2	chr20:33459949-33515769	1.57	$<2.23 \times 10^{-308}$
	TRHDE	chr12:72481046-73059422	1.56	$<2.23 \times 10^{-308}$
	CLMP	chr11:122943035-123065989	1.52	$<2.23 \times 10^{-308}$
	PLIN1	chr15:90207596-90222658	1.51	$<2.23 \times 10^{-308}$

ELMOD3	chr2:85581517-85618875	1.51	$<2.23x10^{-308}$
GPC6	chr13:93879095-95059655	1.50	$<2.23x10^{-308}$
PXDN	chr2:1635659-1748624	1.49	$<2.23x10^{-308}$
CIDEC	chr3:9908398-9921938	1.49	$<2.23x10^{-308}$
AGPAT2	chr9:139567595-139581875	1.48	$<2.23x10^{-308}$
MAST4	chr5:65892176-66465423	1.46	$<2.23x10^{-308}$
RP11-286B14.1	chr1:95975672-96247898	1.46	$<2.23x10^{-308}$
COBLL1	chr2:165510134-165700189	1.45	$<2.23x10^{-308}$
ADRA1A	chr8:26605667-26724790	1.43	$<2.23x10^{-308}$
ADCY5	chr3:123001143-123168605	1.42	$<2.23x10^{-308}$
ASPH	chr8:62413116-62627155	1.42	$<2.23x10^{-308}$
FADS3	chr11:61640991-61659523	1.41	$<2.23x10^{-308}$
PPP2R1B	chr11:111597632-111637151	1.41	$<2.23x10^{-308}$
CACNA2D1	chr7:81575760-82073114	1.41	$<2.23x10^{-308}$
PFKFB1	chrX:54959394-55024967	1.39	$<2.23x10^{-308}$
SEMA3A	chr7:83585093-84122040	1.39	$<2.23x10^{-308}$
SLC7A6	chr16:68298433-68335722	1.38	$<2.23x10^{-308}$
DAPK2	chr15:64199235-64364232	1.38	$<2.23x10^{-308}$
PLA2G16	chr11:63340667-63384355	1.35	$<2.23x10^{-308}$
HK2	chr2:75061108-75120486	1.34	$<2.23x10^{-308}$
GPD1	chr12:50497602-50505102	1.33	$<2.23x10^{-308}$
GLYAT	chr11:58407899-58499447	1.32	$<2.23x10^{-308}$
ADIPOQ	chr3:186560463-186576252	1.30	$<2.23x10^{-308}$
TUSC5	chr17:1182957-1204281	1.29	$<2.23x10^{-308}$
RP11-65J21.3	chr16:14396145-14420210	1.29	$<2.23x10^{-308}$
TENM4	chr11:78363876-79151992	1.27	$<2.23x10^{-308}$
FASN	chr17:80036214-80056208	1.27	$<2.23x10^{-308}$
RETSAT	chr2:85569211-85581743	1.27	$<2.23x10^{-308}$
CTD-2023N9.1	chr5:56690887-56829251	1.26	$<2.23x10^{-308}$
COL8A1	chr3:99357319-99518070	1.25	$<2.23x10^{-308}$
DGAT2	chr11:75470557-75512579	1.25	$<2.23x10^{-308}$
NEDD4L	chr18:55711599-56068772	1.25	$<2.23x10^{-308}$
KCNIP2	chr10:103585731-103603677	1.25	$<2.23x10^{-308}$
VKORC1L1	chr7:65338254-65424550	1.25	$<2.23x10^{-308}$
PECR	chr2:216861052-216947678	1.25	$<2.23x10^{-308}$
MAOA	chrX:43515467-43606068	1.24	$<2.23x10^{-308}$
KCNIP2-AS1	chr10:103578835-103588536	1.24	$<2.23x10^{-308}$
PMEPA1	chr20:56223448-56286592	1.21	$<2.23x10^{-308}$
RP11-798K3.2	chr15:67278699-67351591	1.21	$<2.23x10^{-308}$
YAP1	chr11:101981192-102104154	1.20	$<2.23x10^{-308}$
UGP2	chr2:64068074-64118696	1.20	$<2.23x10^{-308}$
C2CD2	chr21:43305221-43373999	1.19	$<2.23x10^{-308}$
EHBP1	chr2:62900986-63273622	1.19	$<2.23x10^{-308}$
PC	chr11:66615704-66725847	1.17	$<2.23x10^{-308}$
RP11-736K20.5	chr11:86603256-86636079	1.17	$<2.23x10^{-308}$
ANGPTL4	chr19:8428173-8439257	1.16	$<2.23x10^{-308}$

SOX6	chr11:15987995-16761138	1.15	$<2.23\times10^{-308}$
MARC1	chr1:220960101-220987735	1.15	$<2.23\times10^{-308}$
GPT2	chr16:46918290-46965209	1.15	$<2.23\times10^{-308}$
RHOBTB3	chr5:95049226-95160087	1.14	$<2.23\times10^{-308}$
PRKAR2B	chr7:106685094-106802256	1.14	$<2.23\times10^{-308}$
PLOD2	chr3:145787227-145881440	1.13	$<2.23\times10^{-308}$
PFKFB3	chr10:6186881-6277495	1.13	$<2.23\times10^{-308}$
PLIN5	chr19:4522543-4535236	1.12	$<2.23\times10^{-308}$
ATP9A	chr20:50213053-50385173	1.12	$<2.23\times10^{-308}$
PYGL	chr14:51324609-51411454	1.11	$<2.23\times10^{-308}$
KLB	chr4:39408473-39453156	1.11	$<2.23\times10^{-308}$
DAB2IP	chr9:124329336-124547809	1.10	$<2.23\times10^{-308}$
CCDC3	chr10:12938627-13141652	1.10	$<2.23\times10^{-308}$
DHDDS	chr1:26758773-26797785	1.10	$<2.23\times10^{-308}$
NPY1R	chr4:164245113-164265984	1.08	$<2.23\times10^{-308}$
PDE8B	chr5:76506274-76725632	1.08	$<2.23\times10^{-308}$
AR	chrX:66764465-66950461	1.07	$<2.23\times10^{-308}$
PPP1R9A	chr7:94536514-94925727	1.07	$<2.23\times10^{-308}$
RP11-212P7.3	chr7:128095894-128133913	1.07	$<2.23\times10^{-308}$
SKI	chr1:2160134-2241558	1.06	$<2.23\times10^{-308}$
AQPEP	chr5:115298151-115363316	1.05	$<2.23\times10^{-308}$
ACSS3	chr12:81331594-81650533	1.05	$<2.23\times10^{-308}$
MACROD2	chr20:13976015-16033842	1.05	$<2.23\times10^{-308}$
TMEM120B	chr12:122150658-122220907	1.05	$<2.23\times10^{-308}$
KANK4	chr1:62702651-62785085	1.04	$<2.23\times10^{-308}$
ACVR1C	chr2:158383279-158485517	1.04	$<2.23\times10^{-308}$
RP11-163N6.2	chr8:61297147-61429354	1.03	$<2.23\times10^{-308}$
ABCA5	chr17:67240452-67323385	1.03	$<2.23\times10^{-308}$
G0S2	chr1:209848765-209849733	1.03	$<2.23\times10^{-308}$
ALDH2	chr12:112204691-112247782	1.03	$<2.23\times10^{-308}$
APCDD1	chr18:10454625-10489945	1.03	$<2.23\times10^{-308}$
ZNF117	chr7:64432154-64467062	1.01	$<2.23\times10^{-308}$
RP11-511B23.2	chr12:93397191-93609455	1.01	$<2.23\times10^{-308}$
AKAP12	chr6:151561134-151679692	0.990	$<2.23\times10^{-308}$
ATP1A2	chr1:160085549-160113381	0.988	$<2.23\times10^{-308}$
CIDEA	chr18:12254318-12277594	0.986	$<2.23\times10^{-308}$
RP11-420A23.1	chr4:129213906-129440549	0.975	$<2.23\times10^{-308}$
C19orf12	chr19:30191721-30206364	0.972	$<2.23\times10^{-308}$
KCNB1	chr20:47980414-48099184	0.971	$<2.23\times10^{-308}$
ACADL	chr2:211052663-211090215	0.966	$<2.23\times10^{-308}$
SORT1	chr1:109852192-109940573	0.966	$<2.23\times10^{-308}$
C14orf180	chr14:105046021-105056852	0.964	$<2.23\times10^{-308}$
NNMT	chr11:114128509-114184007	0.963	$<2.23\times10^{-308}$
HSDL2	chr9:115142217-115234690	0.963	$<2.23\times10^{-308}$
SPARC	chr5:151040657-151066726	0.943	$<2.23\times10^{-308}$
SOS1	chr2:39208537-39351486	0.942	$<2.23\times10^{-308}$

ECM2	chr9:95256365-95298937	0.930	$<2.23\times10^{-308}$
SGK2	chr20:42187608-42216877	0.923	$<2.23\times10^{-308}$
CAV2	chr7:115927434-116148595	0.907	$<2.23\times10^{-308}$
AC034220.3	chr5:131646978-131705608	0.907	$<2.23\times10^{-308}$
CAP2	chr6:17393447-17558023	0.899	$<2.23\times10^{-308}$
TACC2	chr10:123748689-124014060	0.898	$<2.23\times10^{-308}$
SMAD6	chr15:66994566-67074338	0.895	$<2.23\times10^{-308}$
AIFM2	chr10:71857979-71892690	0.891	$<2.23\times10^{-308}$
ACO1	chr9:32384618-32454767	0.891	$<2.23\times10^{-308}$
DAAM1	chr14:59655364-59838123	0.880	$<2.23\times10^{-308}$
LRIG1	chr3:66429221-66551687	0.880	$<2.23\times10^{-308}$
CSAD	chr12:53551447-53575135	0.876	$<2.23\times10^{-308}$
SNTG2	chr2:946554-1371385	0.868	$<2.23\times10^{-308}$
LGR4	chr11:27387508-27494322	0.868	$<2.23\times10^{-308}$
ADAM12	chr10:127700950-128077024	0.866	$<2.23\times10^{-308}$
TSGA10	chr2:99613724-99771427	0.865	$<2.23\times10^{-308}$
PTPLB	chr3:123209667-123304032	0.864	$<2.23\times10^{-308}$
NRIP1	chr21:16333556-16437321	0.863	$<2.23\times10^{-308}$
SYN3	chr22:32908539-33454358	0.862	$<2.23\times10^{-308}$
GLCCI1	chr7:8008425-8133902	0.862	$<2.23\times10^{-308}$
IRS2	chr13:110406184-110438915	0.859	$<2.23\times10^{-308}$
TWIST1	chr7:19060614-19157295	0.852	$<2.23\times10^{-308}$
RTN3	chr11:63448918-63527363	0.850	$<2.23\times10^{-308}$
FOXO1	chr13:41129804-41240734	0.828	$<2.23\times10^{-308}$
MDFIC	chr7:114562209-114659256	0.827	$<2.23\times10^{-308}$
ITGB1	chr10:33189247-33294720	0.825	$<2.23\times10^{-308}$
RP11-141M1.3	chr13:33929343-34250905	0.820	$<2.23\times10^{-308}$
EMC3	chr3:10004221-10052800	0.815	$<2.23\times10^{-308}$
LINC00278	chrY:2870953-2970313	0.814	$<2.23\times10^{-308}$
RP11-611E13.2	chr12:70297813-70637140	0.810	$<2.23\times10^{-308}$
ESR2	chr14:64550950-64804830	0.806	$<2.23\times10^{-308}$
IRS1	chr2:227596033-227664475	0.804	$<2.23\times10^{-308}$
TULP4	chr6:158733692-158932860	0.802	$<2.23\times10^{-308}$
TLE1	chr9:84198598-84304220	0.795	$<2.23\times10^{-308}$
FREM1	chr9:14734664-14910993	0.791	$<2.23\times10^{-308}$
RP11-175K6.1	chr5:158527491-158544486	0.789	$<2.23\times10^{-308}$
MMD	chr17:53469974-53499353	0.788	$<2.23\times10^{-308}$
CAT	chr11:34460472-34493609	0.785	$<2.23\times10^{-308}$
AP000304.12	chr21:34956993-35284635	0.785	$<2.23\times10^{-308}$
AACS	chr12:125549925-125627873	0.778	$<2.23\times10^{-308}$
TBC1D16	chr17:77906142-78009647	0.774	$<2.23\times10^{-308}$
AC007319.1	chr2:187867947-188419390	0.771	$<2.23\times10^{-308}$
PSMA1	chr11:14515329-14541890	0.760	$<2.23\times10^{-308}$
NLGN1	chr3:173114074-174004434	0.758	$<2.23\times10^{-308}$
PRR5	chr22:45064593-45133561	0.755	$<2.23\times10^{-308}$
ZBTB7C	chr18:45553044-45937123	0.749	$<2.23\times10^{-308}$

LDLRAD3	chr11:35965531-36253686	0.746	$<2.23x10^{-308}$
ARHGAP20	chr11:110447766-110583912	0.745	$<2.23x10^{-308}$
WASF3	chr13:27131840-27263085	0.744	$<2.23x10^{-308}$
RP11-486O13.4	chr14:70892529-71014181	0.739	$<2.23x10^{-308}$
MGEA5	chr10:103544200-103578696	0.731	$<2.23x10^{-308}$
CRY2	chr11:45868669-45904798	0.730	$<2.23x10^{-308}$
LMCD1-AS1	chr3:7994492-8653610	0.729	$<2.23x10^{-308}$
AIG1	chr6:143381633-143661441	0.725	$<2.23x10^{-308}$
RP11-383H13.1	chr8:72740402-73030628	0.724	$<2.23x10^{-308}$
LRP5	chr11:68080077-68216743	0.723	$<2.23x10^{-308}$
ABCD2	chr12:39943835-40013553	0.721	$<2.23x10^{-308}$
SPAG16	chr2:214149113-215275225	0.720	$<2.23x10^{-308}$
ULK4	chr3:41288090-42003922	0.718	$<2.23x10^{-308}$
ARHGEF6	chrX:135747706-135864247	0.718	$<2.23x10^{-308}$
BHMT2	chr5:78365540-78385289	0.718	$<2.23x10^{-308}$
DPT	chr1:168664697-168698502	0.714	$<2.23x10^{-308}$
FZD4	chr11:86656721-86666433	0.713	$<2.23x10^{-308}$
MYOM1	chr18:3066805-3220106	0.708	$<2.23x10^{-308}$
ANO6	chr12:45609770-45834187	0.707	$<2.23x10^{-308}$
FGF2	chr4:123747863-123819391	0.700	$<2.23x10^{-308}$
EYS	chr6:64429876-66417118	0.699	$<2.23x10^{-308}$
USP33	chr1:78161672-78225537	0.690	$<2.23x10^{-308}$
SLC25A16	chr10:70237756-70287231	0.690	$<2.23x10^{-308}$
CDC14B	chr9:99252523-99382112	0.687	$<2.23x10^{-308}$
ACOX1	chr17:73937588-73975515	0.682	$<2.23x10^{-308}$
MID1	chrX:10413350-10851773	0.681	$<2.23x10^{-308}$
PPP2R5A	chr1:212458879-212535200	0.676	$<2.23x10^{-308}$
GS1-124K5.10	chr7:65976543-66010053	0.668	$<2.23x10^{-308}$
GABRE	chrX:151121596-151143152	0.668	$<2.23x10^{-308}$
FSTL3	chr19:676392-683385	0.665	$<2.23x10^{-308}$
SREBF1	chr17:17713713-17740325	0.665	$<2.23x10^{-308}$
PNPLA7	chr9:140354404-140444986	0.663	$<2.23x10^{-308}$
AQP7P1	chr9:67272038-67289492	0.660	$<2.23x10^{-308}$
TMEM164	chrX:109245859-109425962	0.657	$<2.23x10^{-308}$
ADHFE1	chr8:67342420-67383836	0.657	$<2.23x10^{-308}$
SLC16A7	chr12:59989848-60176395	0.657	$<2.23x10^{-308}$
RP11-597D13.9	chr4:159091904-159124029	0.639	$<2.23x10^{-308}$
SNX21	chr20:44462449-44471914	0.637	$<2.23x10^{-308}$
FAM214A	chr15:52873514-53002014	0.630	$<2.23x10^{-308}$
LURAP1L	chr9:12775020-12822130	0.630	$<2.23x10^{-308}$
LOXL2	chr8:23154702-23282841	0.627	$<2.23x10^{-308}$
LIMS1	chr2:109150857-109303702	0.625	$<2.23x10^{-308}$
DHRS3	chr1:12627939-12677737	0.619	$<2.23x10^{-308}$
PABPN1	chr14:23790498-23795394	0.617	$<2.23x10^{-308}$
TMEM144	chr4:159122756-159176563	0.616	$<2.23x10^{-308}$
MAP7D1	chr1:36621180-36646450	0.614	$<2.23x10^{-308}$

STAT5A	chr17:40439565-40463961	0.613	$<2.23\times10^{-308}$
AL163953.3	chr14:53635772-54153976	0.601	$<2.23\times10^{-308}$
SRPX2	chrX:99899215-99926296	0.598	$<2.23\times10^{-308}$
GPI	chr19:34850385-34893061	0.597	$<2.23\times10^{-308}$
RARRES2	chr7:150035408-150038763	0.597	$<2.23\times10^{-308}$
VEGFA	chr6:43737921-43754224	0.594	$<2.23\times10^{-308}$
IDH1	chr2:209100951-209130798	0.593	$<2.23\times10^{-308}$
LARP1	chr5:154092462-154197167	0.589	$<2.23\times10^{-308}$
ITGB1BP1	chr2:9543604-9563676	0.585	$<2.23\times10^{-308}$
CRLS1	chr20:5986736-6020699	0.583	$<2.23\times10^{-308}$
MYH10	chr17:8377523-8534079	0.580	$<2.23\times10^{-308}$
TNIP1	chr5:150409506-150473138	0.577	$<2.23\times10^{-308}$
RCL1	chr9:4792869-4885917	0.576	$<2.23\times10^{-308}$
CDHR3	chr7:105517242-105676877	0.575	$<2.23\times10^{-308}$
AC092594.1	chr2:19167729-19546509	0.568	$<2.23\times10^{-308}$
RP5-864K19.4	chr1:39325672-39385068	0.555	$<2.23\times10^{-308}$
GREB1L	chr18:18822203-19105378	0.550	$<2.23\times10^{-308}$
ELOVL5	chr6:53132196-53213947	0.540	$<2.23\times10^{-308}$
MAGI2-AS3	chr7:79082198-79100524	0.539	$<2.23\times10^{-308}$
TFDP2	chr3:141663277-141868386	0.536	$<2.23\times10^{-308}$
YWHAG	chr7:75956116-75988348	0.535	$<2.23\times10^{-308}$
CTD-2337A12.1	chr5:95297705-95966789	0.535	$<2.23\times10^{-308}$
DCUN1D4	chr4:52709166-52783003	0.534	$<2.23\times10^{-308}$
GPX4	chr19:1103936-1106787	0.530	$<2.23\times10^{-308}$
SLTM	chr15:59171244-59225852	0.529	$<2.23\times10^{-308}$
GSE1	chr16:85645015-85709810	0.528	$<2.23\times10^{-308}$
RNPC3	chr1:104068313-104097861	0.524	$<2.23\times10^{-308}$
GIPC1	chr19:14588572-14606944	0.521	$<2.23\times10^{-308}$
PLEKHG5	chr1:6526152-6580121	0.518	$<2.23\times10^{-308}$
BLCAP	chr20:36120874-36156333	0.516	$<2.23\times10^{-308}$
HIBCH	chr2:191054461-191208919	0.515	$<2.23\times10^{-308}$
ADIPOR2	chr12:1797740-1897844	0.515	$<2.23\times10^{-308}$
KIAA1377	chr11:101785746-101871789	0.514	$<2.23\times10^{-308}$
TNKS1BP1	chr11:57067112-57092426	0.510	$<2.23\times10^{-308}$
BNIP3L	chr8:26240414-26363152	0.497	$<2.23\times10^{-308}$
PRSS23	chr11:86502101-86663952	0.490	$<2.23\times10^{-308}$
RAD23B	chr9:110045418-110094475	0.490	$<2.23\times10^{-308}$
SLC7A6OS	chr16:68318406-68344849	0.486	$<2.23\times10^{-308}$
DLG1	chr3:196769431-197026171	0.486	$<2.23\times10^{-308}$
AAMDC	chr11:77532155-77629478	0.485	$<2.23\times10^{-308}$
PLCE1	chr10:95753746-96092580	0.485	$<2.23\times10^{-308}$
VEGFB	chr11:64002010-64006259	0.478	$<2.23\times10^{-308}$
ITPK1	chr14:93403259-93582665	0.473	$<2.23\times10^{-308}$
AKT2	chr19:40736224-40791443	0.471	$<2.23\times10^{-308}$
PLEKHM3	chr2:208693027-208890284	0.470	$<2.23\times10^{-308}$
BCL2L13	chr22:18111621-18213388	0.469	$<2.23\times10^{-308}$

DENND5B	chr12:31535157-31744031	0.464	$<2.23x10^{-308}$
LPGAT1	chr1:211916799-212004114	0.464	$<2.23x10^{-308}$
TNFRSF25	chr1:6521211-6526255	0.460	$<2.23x10^{-308}$
JADE1	chr4:129730779-129796379	0.457	$<2.23x10^{-308}$
USP40	chr2:234384166-234475428	0.453	$<2.23x10^{-308}$
PLEKHH2	chr2:43864412-43995126	0.452	$<2.23x10^{-308}$
LEP	chr7:127881337-127897681	0.451	$<2.23x10^{-308}$
TAPT1-AS1	chr4:16228286-16321763	0.447	$<2.23x10^{-308}$
GPC3	chrX:132669773-133119922	0.446	$<2.23x10^{-308}$
NPLOC4	chr17:79523913-79615495	0.444	$<2.23x10^{-308}$
SLC27A1	chr19:17579578-17616977	0.443	$<2.23x10^{-308}$
KANSL1L	chr2:210886147-211036107	0.438	$<2.23x10^{-308}$
MCCC1	chr3:182733006-182833863	0.437	$<2.23x10^{-308}$
NHLRC3	chr13:39612443-39624246	0.436	$<2.23x10^{-308}$
EFEMP1	chr2:56093102-56151274	0.433	$<2.23x10^{-308}$
SIL1	chr5:138282409-138629246	0.433	$<2.23x10^{-308}$
SERPINF1	chr17:1665253-1680868	0.429	$<2.23x10^{-308}$
TSEN2	chr3:12525931-12581122	0.419	$<2.23x10^{-308}$
GS1-124K5.12	chr7:66010634-66057373	0.418	$<2.23x10^{-308}$
FAXDC2	chr5:154198051-154238812	0.417	$<2.23x10^{-308}$
FAM69A	chr1:93307724-93427057	0.414	$<2.23x10^{-308}$
SH2B1	chr16:28857921-28885526	0.403	$<2.23x10^{-308}$
EPHX1	chr1:225997794-226033260	0.398	$<2.23x10^{-308}$
NMT2	chr10:15144583-15210692	0.394	$<2.23x10^{-308}$
EIF2B3	chr1:45316450-45452282	0.392	$<2.23x10^{-308}$
MICAL3	chr22:18270415-18507325	0.388	$<2.23x10^{-308}$
GS1-124K5.11	chr7:65991075-66007536	0.387	$<2.23x10^{-308}$
RP11-509J21.1	chr9:3526723-3671646	0.385	$<2.23x10^{-308}$
UBR3	chr2:170683968-170940641	0.385	$<2.23x10^{-308}$
ECHDC1	chr6:127609855-127664754	0.384	$<2.23x10^{-308}$
OPTN	chr10:13141449-13180291	0.383	$<2.23x10^{-308}$
CAPS2	chr12:75669759-75784708	0.375	$<2.23x10^{-308}$
BCKDHB	chr6:80816364-81055987	0.375	$<2.23x10^{-308}$
ACYP2	chr2:54197975-54532437	0.374	$<2.23x10^{-308}$
FLJ27365	chr22:46449749-46509808	0.371	$<2.23x10^{-308}$
C1orf132	chr1:207974863-208042495	0.370	$<2.23x10^{-308}$
SH3BP5	chr3:15296360-15382875	0.367	$<2.23x10^{-308}$
ETFA	chr15:76507696-76603813	0.363	$<2.23x10^{-308}$
ZNF248	chr10:38091751-38147034	0.362	$<2.23x10^{-308}$
ZRANB3	chr2:135894486-136288806	0.360	$<2.23x10^{-308}$
CD46	chr1:207925402-207968858	0.356	$<2.23x10^{-308}$
MNAT1	chr14:61201460-61436671	0.352	$<2.23x10^{-308}$
AOX1	chr2:201450591-201541787	0.348	$<2.23x10^{-308}$
C6orf106	chr6:34555065-34664636	0.347	$<2.23x10^{-308}$
MOB3B	chr9:27325207-27529779	0.343	$<2.23x10^{-308}$
ETFDH	chr4:159593277-159630775	0.343	$<2.23x10^{-308}$

	RUSC2	chr9:35490124-35561895	0.342	$<2.23\times10^{-308}$
	MTERF	chr7:91321323-91510034	0.337	$<2.23\times10^{-308}$
	PIEZO1	chr16:88781751-88851619	0.337	$<2.23\times10^{-308}$
	ENOSF1	chr18:670324-712676	0.332	$<2.23\times10^{-308}$
	ESR1	chr6:151977826-152450754	0.326	$<2.23\times10^{-308}$
	RP11-33B1.1	chr4:120375946-120473180	0.319	$<2.23\times10^{-308}$
	STK38L	chr12:27396901-27478892	0.319	$<2.23\times10^{-308}$
	MSH3	chr5:79950467-80172279	0.318	$<2.23\times10^{-308}$
	ZFYVE9	chr1:52608046-52812358	0.318	$<2.23\times10^{-308}$
	CTTN	chr11:70244510-70282690	0.317	$<2.23\times10^{-308}$
	AC002117.1	chr17:43227767-43238877	0.315	$<2.23\times10^{-308}$
	RP11-37B2.1	chr8:90621638-90769955	0.310	$<2.23\times10^{-308}$
	FAM107B	chr10:14560556-14816896	0.310	$<2.23\times10^{-308}$
	KLHDC10	chr7:129710350-129775560	0.305	$<2.23\times10^{-308}$
	PISD	chr22:32014477-32058418	0.305	$<2.23\times10^{-308}$
	WDR20	chr14:102605840-102691184	0.304	$<2.23\times10^{-308}$
	MTA3	chr2:42721709-42984087	0.302	$<2.23\times10^{-308}$
	ARHGAP32	chr11:128834955-129149219	0.294	$<2.23\times10^{-308}$
	NNT	chr5:43602794-43707507	0.282	$<2.23\times10^{-308}$
	RAB30-AS1	chr11:82783108-82817761	0.275	$<2.23\times10^{-308}$
	WDR60	chr7:158649269-158749438	0.274	$<2.23\times10^{-308}$
	KLHDC1	chr14:50159823-50219870	0.273	$<2.23\times10^{-308}$
	DECR1	chr8:91013633-91064320	0.269	$<2.23\times10^{-308}$
	CAPN7	chr3:15247659-15294425	0.266	$<2.23\times10^{-308}$
	SLC22A23	chr6:3269196-3457256	0.255	$<2.23\times10^{-308}$
	TUBB6	chr18:12307668-12344319	0.319	2.70×10^{-302}
	VWA8	chr13:42140973-42535256	0.255	6.30×10^{-297}
	GUSBP1	chr5:21341942-21589481	0.252	4.85×10^{-296}
	PTCHD3P1	chr10:29698331-29776674	0.261	3.14×10^{-293}
	TBC1D2B	chr15:78276378-78370066	0.268	8.41×10^{-282}
	RAB2A	chr8:61429416-61536186	0.331	4.58×10^{-280}
	HMGB1	chr13:31032884-31191734	0.259	1.41×10^{-277}
	TTC3	chr21:38445526-38575413	0.329	9.71×10^{-277}
	CMSS1	chr3:99536678-99897447	0.289	1.35×10^{-257}
	PCCA	chr13:100741269-101182686	0.264	3.93×10^{-245}
	MBNL2	chr13:97873688-98046374	0.271	3.02×10^{-229}
	SAA1	chr11:18287721-18291524	0.276	4.60×10^{-126}
B cells	FCRL1	chr1:157764193-157789895	3.93	$<2.23\times10^{-308}$
	BANK1	chr4:102332443-102995969	3.89	$<2.23\times10^{-308}$
	RALGPS2	chr1:178694300-178889238	3.63	$<2.23\times10^{-308}$
	MS4A1	chr11:60223225-60238233	3.53	$<2.23\times10^{-308}$
	GNG7	chr19:2511217-2702707	3.13	$<2.23\times10^{-308}$
	BLK	chr8:11351510-11422113	3.11	$<2.23\times10^{-308}$
	FAM129C	chr19:17634110-17664648	3.06	$<2.23\times10^{-308}$
	PAX5	chr9:36833272-37034103	2.87	$<2.23\times10^{-308}$
	LINC00926	chr15:57592563-57599959	2.46	$<2.23\times10^{-308}$

	CD22	chr19:35810164-35838258	2.31	$<2.23 \times 10^{-308}$
	ADAM19	chr5:156822542-157002783	2.33	6.16×10^{-218}
	TPD52	chr8:80870571-81143467	2.32	2.14×10^{-190}
	SP140	chr2:231067826-231223762	2.00	2.32×10^{-155}
	CTD-2207O23.3	chr19:7445850-7535131	2.09	2.67×10^{-136}
	IKZF3	chr17:37921198-38020441	1.85	1.59×10^{-131}
	STRBP	chr9:125871779-126030855	2.51	3.73×10^{-114}
	SP110	chr2:231032009-231090444	1.40	1.28×10^{-32}
	SWAP70	chr11:9685624-9774538	1.46	7.88×10^{-20}
	ZCCHC7	chr9:37120536-37358146	1.39	2.26×10^{-16}
	ARID5B	chr10:63661059-63856703	1.31	1.38×10^{-11}
	PRDM2	chr1:14026693-14151574	1.05	0.0217
CD4+ T-cells	LEF1	chr4:108968701-109090112	2.66	$<2.23 \times 10^{-308}$
	OXNAD1	chr3:16306706-16391806	1.75	9.01×10^{-183}
	MLLT3	chr9:20341663-20622542	1.49	4.06×10^{-107}
	GLG1	chr16:74485856-74641012	0.74	2.66×10^{-4}
	ARID4B	chr1:235294949-235491534	0.63	1.25×10^{-3}
	RP4-717I23.3	chr1:93727743-93811582	0.28	3.47×10^{-3}
	ZMYM2	chr13:20532810-20665968	0.28	7.09×10^{-3}
CD8+ T-cells	CD2	chr1:117297007-117311850	1.88	7.20×10^{-202}
	SCML4	chr6:108025308-108145521	1.58	2.49×10^{-116}
	AC104820.2	chr2:181966659-182264286	1.84	7.93×10^{-108}
	SLC38A1	chr12:46576846-46663800	1.45	1.78×10^{-107}
	CYFIP2	chr5:156693089-156822606	1.65	4.45×10^{-107}
	ACAP1	chr17:7239848-7254797	1.50	1.17×10^{-96}
	CLEC2D	chr12:9817565-9848413	1.18	5.24×10^{-26}
	CNOT6L	chr4:78634541-78740769	0.986	1.09×10^{-17}
	MGAT4A	chr2:99235569-99347589	1.01	1.36×10^{-17}
	KIF2A	chr5:61601989-61833076	0.921	3.19×10^{-13}
	PTPN4	chr2:120517207-120741394	0.895	2.63×10^{-10}
	SYNRG	chr17:35874900-35969544	0.838	4.87×10^{-9}
	PCSK7	chr11:117075053-117103241	0.815	8.44×10^{-7}
	KAT2B	chr3:20081515-20195896	0.819	6.98×10^{-5}
	CCSER2	chr10:86088342-86278273	0.772	3.81×10^{-4}
	OGT	chrX:70752933-70795747	0.644	1.01×10^{-3}
Endothelial	LNX1	chr4:54325468-54567572	2.57	$<2.23 \times 10^{-308}$
	MCF2L	chr13:113548692-113754053	2.39	$<2.23 \times 10^{-308}$
	CXorf36	chrX:45007619-45060146	2.36	$<2.23 \times 10^{-308}$
	CYYR1	chr21:27838528-27945603	2.31	$<2.23 \times 10^{-308}$
	PIK3R3	chr1:46505812-46642160	2.27	$<2.23 \times 10^{-308}$
	PLCB4	chr20:9049410-9461889	2.26	$<2.23 \times 10^{-308}$
	DACH1	chr13:72012098-72441330	2.20	$<2.23 \times 10^{-308}$
	RAPGEF3	chr12:48128455-48164823	2.17	$<2.23 \times 10^{-308}$
	BMP6	chr6:7727030-7881655	2.14	$<2.23 \times 10^{-308}$
	MYO5C	chr15:52484519-52587995	2.13	$<2.23 \times 10^{-308}$
	NR5A2	chr1:199996730-200146552	2.07	$<2.23 \times 10^{-308}$

	ROBO4	chr11:124753587-124768396	2.00	$<2.23x10^{-308}$
	SGK223	chr8:8175258-8244008	1.99	$<2.23x10^{-308}$
	FLT1	chr13:28874489-29069265	1.96	$<2.23x10^{-308}$
	GALNT18	chr11:11292423-11643552	1.95	$<2.23x10^{-308}$
	RBP7	chr1:10057264-10076078	1.95	$<2.23x10^{-308}$
	ITGA6	chr2:173292082-173371181	1.90	$<2.23x10^{-308}$
	CD300LG	chr17:41924516-41940997	1.87	$<2.23x10^{-308}$
	SORBS2	chr4:186506598-186877806	1.85	$<2.23x10^{-308}$
	KANK3	chr19:8387468-8408146	1.83	$<2.23x10^{-308}$
	LAMA3	chr18:21269407-21535030	1.82	$<2.23x10^{-308}$
	PPP1R16B	chr20:37434348-37551667	1.73	$<2.23x10^{-308}$
	CLDN5	chr22:19510547-19515068	1.69	$<2.23x10^{-308}$
	LIMS2	chr2:128395956-128439360	1.64	$<2.23x10^{-308}$
	GALNT15	chr3:16216156-16273499	1.60	$<2.23x10^{-308}$
	ENPP2	chr8:120569326-120685693	2.08	$1.44x10^{-253}$
	GFOD1	chr6:13358062-13487894	1.64	$3.16x10^{-251}$
	CTNNBIP1	chr1:9908334-9970394	1.73	$2.70x10^{-233}$
	ICA1	chr7:8152814-8302317	1.54	$4.74x10^{-223}$
	DNM3	chr1:171810621-172387606	1.57	$2.42x10^{-220}$
	FGD5	chr3:14860469-14975895	1.40	$2.02x10^{-204}$
	PTPN14	chr1:214522039-214725792	1.38	$1.17x10^{-118}$
	MCC	chr5:112357796-112824527	1.31	$4.62x10^{-116}$
	MPDZ	chr9:13105703-13279589	1.19	$7.49x10^{-97}$
	RIMKLB	chr12:8834196-8935691	1.25	$2.00x10^{-83}$
	TBCD	chr17:80709940-80900724	1.13	$5.98x10^{-76}$
	CARD8	chr19:48684027-48759203	0.90	$7.80x10^{-72}$
	WNK1	chr12:861759-1020618	0.82	$4.55x10^{-38}$
	NKTR	chr3:42642106-42690227	0.71	$1.35x10^{-26}$
	KAT6A	chr8:41786997-41909508	0.45	$3.80x10^{-10}$
Fibroblasts	FBN1	chr15:48700503-48938046	3.23	$<2.23x10^{-308}$
	GFPT2	chr5:179727690-179780387	2.48	$<2.23x10^{-308}$
	PTGIS	chr20:48120411-48184683	2.48	$<2.23x10^{-308}$
	SEMA3C	chr7:80371854-80551675	2.44	$<2.23x10^{-308}$
	KCNB2	chr8:73449626-73850584	2.41	$<2.23x10^{-308}$
	MFAP5	chr12:8789942-8815484	2.41	$<2.23x10^{-308}$
	GPR133	chr12:131438452-131626014	2.31	$<2.23x10^{-308}$
	ITGA11	chr15:68594050-68724501	2.26	$<2.23x10^{-308}$
	SCARA5	chr8:27727399-27850244	2.22	$<2.23x10^{-308}$
	DPYSL3	chr5:146770374-146889619	2.00	$<2.23x10^{-308}$
	STK32B	chr4:5053169-5502725	1.92	$<2.23x10^{-308}$
	ADAMTSL1	chr9:18473892-18910948	1.91	$<2.23x10^{-308}$
	TRIO	chr5:14143811-14532235	1.87	$<2.23x10^{-308}$
	MEG8	chr14:101361107-101402336	1.87	$<2.23x10^{-308}$
	HUNK	chr21:33245628-33416946	1.80	$<2.23x10^{-308}$
	PI16	chr6:36922209-36932613	1.80	$<2.23x10^{-308}$
	AXL	chr19:41725108-41767671	1.78	$<2.23x10^{-308}$

HTRA3	chr4:8271492-8308838	1.75	$<2.23x10^{-308}$
FSTL1	chr3:120111140-120170100	1.70	$<2.23x10^{-308}$
CNTN4	chr3:2140497-3099645	1.68	$<2.23x10^{-308}$
HMCN2	chr9:133046882-133309510	1.67	$<2.23x10^{-308}$
MRC2	chr17:60704762-60770958	1.61	$<2.23x10^{-308}$
SULF1	chr8:70378859-70573150	1.61	$<2.23x10^{-308}$
CD55	chr1:207494853-207534311	1.59	$<2.23x10^{-308}$
ADAMTS5	chr21:28290231-28338832	1.56	$<2.23x10^{-308}$
ARHGEF10	chr8:1772142-1906807	1.52	$<2.23x10^{-308}$
GALNT13	chr2:154728426-155310361	1.52	$<2.23x10^{-308}$
ACKR3	chr2:237476430-237491001	1.50	$<2.23x10^{-308}$
TBC1D12	chr10:96162261-96295687	1.47	$<2.23x10^{-308}$
ANTXR1	chr2:69240310-69476459	1.46	$<2.23x10^{-308}$
COL5A1	chr9:137533620-137736686	1.41	$<2.23x10^{-308}$
UAP1	chr1:162531323-162569627	1.41	$<2.23x10^{-308}$
FN1	chr2:216225163-216300895	1.41	$<2.23x10^{-308}$
STON2	chr14:81727000-81902809	1.39	$<2.23x10^{-308}$
PXN	chr12:120648250-120703574	1.33	$<2.23x10^{-308}$
FLRT2	chr14:85996488-86095034	1.30	$<2.23x10^{-308}$
CD34	chr1:208057594-208084747	1.14	$<2.23x10^{-308}$
LTBP4	chr19:41098789-41135725	1.15	$1.92x10^{-287}$
TENM1	chrX:123509753-124097666	1.20	$5.42x10^{-284}$
RECK	chr9:36036430-36124448	1.13	$1.43x10^{-258}$
TRIP10	chr19:6737936-6751537	1.17	$1.33x10^{-243}$
SPTAN1	chr9:131314866-131395941	1.02	$8.03x10^{-216}$
PHLPP1	chr18:60382672-60647666	1.19	$9.80x10^{-192}$
ROR1	chr1:64239693-64647181	1.07	$6.68x10^{-165}$
SMIM14	chr4:39547950-39640710	1.04	$3.98x10^{-161}$
COL12A1	chr6:75794042-75915767	1.01	$7.72x10^{-140}$
FAM114A1	chr4:38869298-38947360	0.971	$1.09x10^{-137}$
HIVEP1	chr6:12008995-12165232	0.821	$1.92x10^{-130}$
SMAD3	chr15:67356101-67487533	0.839	$1.09x10^{-127}$
PLAC9	chr10:81891438-81905115	0.816	$1.58x10^{-109}$
CDC42BPB	chr14:103398716-103523799	0.868	$1.39x10^{-102}$
HDAC4	chr2:239969864-240323348	0.640	$2.27x10^{-76}$
UPF3A	chr13:115047059-115071283	0.695	$4.03x10^{-70}$
ARHGAP12	chr10:32094365-32217742	0.663	$1.29x10^{-68}$
PTPRA	chr20:2844830-3019722	0.512	$2.91x10^{-52}$
ABL1	chr9:133589333-133763062	0.581	$2.24x10^{-51}$
PPP2R2A	chr8:26149007-26230196	0.591	$1.42x10^{-50}$
STIM1	chr11:3875757-4114439	0.560	$5.22x10^{-50}$
IL6ST	chr5:55230923-55290821	0.579	$1.13x10^{-48}$
WWOX	chr16:78133310-79246564	0.370	$4.33x10^{-37}$
C5orf42	chr5:37106330-37249530	0.536	$2.09x10^{-34}$
BBS9	chr7:33168856-33645680	0.526	$3.32x10^{-34}$
ZNF83	chr19:53097313-53193749	0.557	$2.36x10^{-31}$

	CCNL2	chr1:1321091-1334708	0.543	$2.38x10^{-31}$
	NCOA7	chr6:126102307-126252266	0.420	$9.85x10^{-24}$
	WWC2	chr4:184020446-184241930	0.463	$6.06x10^{-21}$
	SMC5	chr9:72873937-72969804	0.328	$4.62x10^{-20}$
	ANKRD17	chr4:73939093-74124515	0.318	$7.94x10^{-20}$
	SEC31A	chr4:83739814-83822319	0.377	$1.95x10^{-18}$
	LRCH3	chr3:197518097-197615307	0.314	$1.02x10^{-14}$
	FAM35A	chr10:88853918-88951225	0.376	$7.08x10^{-14}$
	SFPQ	chr1:35641979-35658749	0.307	$1.82x10^{-9}$
	ADD1	chr4:2845584-2931803	0.297	$4.05x10^{-9}$
	PRUNE2	chr9:79226292-79521003	0.281	$6.43x10^{-9}$
	HNRNPDL	chr4:83343717-83351294	0.296	$5.41x10^{-8}$
	HECTD4	chr12:112597992-112819896	0.269	$7.08x10^{-7}$
	PPP3CB	chr10:75196186-75255782	0.281	$1.89x10^{-6}$
Heptoglobin	FLT3	chr13:28577411-28674729	3.48	$<2.23x10^{-308}$
	IL1R2	chr2:102608306-102645006	2.31	$2.06x10^{-237}$
	NAPSB	chr19:50837053-50848024	1.97	$8.72x10^{-210}$
	HLA-DQA1	chr6:32595956-32614839	1.67	$4.67x10^{-112}$
	CBFA2T3	chr16:88941266-89043612	1.94	$8.34x10^{-96}$
	AGPAT9	chr4:84457067-84527028	1.96	$1.66x10^{-90}$
	CLEC10A	chr17:6977856-6983626	2.04	$8.37x10^{-84}$
	RUNX2	chr6:45295894-45632086	1.65	$7.52x10^{-51}$
	KCNK6	chr19:38810484-38819660	1.60	$5.63x10^{-46}$
	CYTIP	chr2:158271131-158345473	1.32	$7.11x10^{-46}$
	WDR49	chr3:167196472-167371771	1.54	$1.13x10^{-40}$
	UBASH3B	chr11:122526383-122685181	1.16	$2.31x10^{-27}$
	PAK1	chr11:77032752-77185680	1.38	$2.84x10^{-22}$
	CCDC6	chr10:61548521-61666414	0.946	$5.67x10^{-16}$
	MOB1B	chr4:71768043-71888166	1.31	$3.30x10^{-14}$
	C22orf34	chr22:49808176-50051190	1.12	$1.16x10^{-11}$
	FAM135A	chr6:71122644-71270877	1.15	$1.36x10^{-11}$
	FAM117B	chr2:203499901-203634480	1.07	$1.65x10^{-11}$
	KIAA0247	chr14:70078313-70181859	1.08	$2.19x10^{-9}$
	SUZ12P	chr17:29036317-29117926	1.05	$5.81x10^{-9}$
	CEP128	chr14:80943330-81425861	0.975	$2.20x10^{-8}$
	MAP3K14	chr17:43340488-43394414	1.10	$3.33x10^{-8}$
	RNF4	chr4:2463947-2627047	0.90	$1.52x10^{-7}$
	STIM2	chr4:26859300-27027003	0.89	$7.32x10^{-7}$
	AHCYL2	chr7:128864864-129070052	0.94	$1.12x10^{-5}$
	FARS2	chr6:5261277-5771813	1.10	$2.56x10^{-4}$
Macrophages	PLA2G7	chr6:46671938-46703430	2.64	$<2.23x10^{-308}$
	LINC01010	chr6:134758854-134825719	2.26	$<2.23x10^{-308}$
	SDS	chr12:113830250-113864106	2.14	$<2.23x10^{-308}$
	ITGA3	chr17:48133332-48167845	2.05	$<2.23x10^{-308}$
	SPOCD1	chr1:32256023-32281652	2.01	$<2.23x10^{-308}$
	TM4SF19	chr3:196046213-196065374	1.95	$<2.23x10^{-308}$

TM4SF19-AS1	chr3:196045201-196052441	1.86	$<2.23\text{x}10^{-308}$
RP11-807H22.7	chr11:71874366-71920285	1.25	$<2.23\text{x}10^{-308}$
KCNE1	chr21:35818988-35884573	2.06	$1.31\text{x}10^{-302}$
SLC26A11	chr17:78193498-78227299	2.30	$2.63\text{x}10^{-252}$
CYP27A1	chr2:219646472-219680016	2.67	$1.33\text{x}10^{-251}$
ACP5	chr19:11685475-11689823	1.82	$2.93\text{x}10^{-223}$
ST18	chr8:53023399-53373519	1.83	$1.04\text{x}10^{-196}$
CDCP1	chr3:45123770-45187914	1.24	$4.38\text{x}10^{-196}$
APOC1	chr19:45417504-45422606	1.84	$1.76\text{x}10^{-194}$
DHRS9	chr2:169921299-169952677	1.73	$7.16\text{x}10^{-191}$
ABCC3	chr17:48712138-48769613	1.93	$8.90\text{x}10^{-190}$
TBC1D2	chr9:100961311-101017915	1.74	$3.41\text{x}10^{-185}$
CHIT1	chr1:203181955-203242769	1.89	$5.39\text{x}10^{-179}$
ZMIZ1-AS1	chr10:80703085-80827652	1.96	$1.38\text{x}10^{-174}$
FABP5	chr8:82192598-82197012	1.61	$2.61\text{x}10^{-170}$
GPC4	chrX:132434131-132549518	2.44	$1.55\text{x}10^{-167}$
DOCK3	chr3:50712672-51421629	2.66	$8.43\text{x}10^{-163}$
IFITM10	chr11:1753640-1771821	1.24	$2.28\text{x}10^{-158}$
TFRC	chr3:195754054-195809060	2.44	$7.56\text{x}10^{-152}$
FBP1	chr9:97365415-97402531	1.27	$5.92\text{x}10^{-150}$
CAPG	chr2:85621871-85645555	1.45	$3.62\text{x}10^{-149}$
TTYH3	chr7:2671585-2704436	1.91	$6.15\text{x}10^{-148}$
AQP9	chr15:58430368-58478110	0.94	$5.34\text{x}10^{-146}$
GPNMB	chr7:23275586-23314727	2.26	$2.28\text{x}10^{-143}$
MATK	chr19:3777971-3802127	1.75	$5.30\text{x}10^{-135}$
MMP9	chr20:44637547-44645200	1.99	$8.07\text{x}10^{-127}$
CD83	chr6:14117872-14137149	1.66	$1.03\text{x}10^{-123}$
LIPA	chr10:90973326-91174314	2.08	$1.44\text{x}10^{-121}$
KCNK13	chr14:90528109-90652201	1.48	$4.89\text{x}10^{-121}$
NR1H3	chr11:47269851-47290396	2.24	$1.43\text{x}10^{-120}$
KCNJ5	chr11:128761251-128790930	1.06	$5.28\text{x}10^{-118}$
CSTB	chr21:45192393-45196326	1.59	$3.12\text{x}10^{-117}$
P2RX4	chr12:121647660-121671909	1.84	$5.15\text{x}10^{-114}$
MFSD12	chr19:3538259-3574288	1.75	$2.05\text{x}10^{-113}$
SLC9A7	chrX:46464753-46618490	1.13	$2.13\text{x}10^{-113}$
ATF3	chr1:212738676-212794119	1.42	$1.61\text{x}10^{-111}$
FAM20C	chr7:192969-300711	2.01	$2.31\text{x}10^{-110}$
ST14	chr11:130029457-130080271	1.01	$1.63\text{x}10^{-108}$
MICAL1	chr6:109765265-109787171	1.36	$4.98\text{x}10^{-102}$
TMEM51	chr1:15479028-15546976	1.30	$2.93\text{x}10^{-100}$
ACOT11	chr1:55007930-55104865	1.03	$1.97\text{x}10^{-97}$
AC005786.7	chr19:3544197-3557567	1.04	$1.05\text{x}10^{-95}$
RASGRP3	chr2:33661391-33789817	1.48	$1.44\text{x}10^{-94}$
KCP	chr7:128502505-128550773	1.68	$7.11\text{x}10^{-93}$
SLC15A3	chr11:60704556-60720002	0.920	$3.39\text{x}10^{-92}$
MMP19	chr12:56229217-56236750	2.15	$4.75\text{x}10^{-92}$

SLC29A3	chr10:73079015-73123142	0.920	$1.11x10^{-88}$
SNHG12	chr1:28905050-28909495	1.20	$1.68x10^{-87}$
ITGAE	chr17:3617922-3704537	1.29	$3.90x10^{-86}$
FERMT3	chr11:63974150-63991354	1.33	$1.35x10^{-85}$
LAPTM5	chr1:31205316-31230667	1.21	$1.88x10^{-84}$
GK	chrX:30671476-30748725	1.07	$3.47x10^{-84}$
RPS6KA1	chr1:26856252-26901521	1.09	$4.00x10^{-83}$
LGALS9	chr17:25956824-25976586	1.20	$6.81x10^{-83}$
CTSL	chr9:90340434-90346308	1.59	$1.28x10^{-81}$
SLC6A6	chr3:14444076-14530857	1.30	$4.03x10^{-81}$
PLAUR	chr19:44150247-44174699	1.36	$1.50x10^{-77}$
GM2A	chr5:150591711-150650001	0.734	$1.85x10^{-77}$
GSTO1	chr10:105995114-106027217	0.936	$3.77x10^{-73}$
SLC22A15	chr1:116519119-116612675	1.15	$1.40x10^{-72}$
GALM	chr2:38893052-38968379	1.43	$5.82x10^{-72}$
SOAT1	chr1:179262925-179327815	1.46	$9.76x10^{-69}$
LY86	chr6:6588341-6655216	0.821	$2.73x10^{-68}$
HEXB	chr5:73935848-74018472	1.30	$1.99x10^{-67}$
THEMIS2	chr1:28199055-28213196	1.07	$9.69x10^{-67}$
P2RX7	chr12:121570622-121623876	1.24	$1.00x10^{-64}$
CD84	chr1:160510885-160549306	1.127	$6.34x10^{-64}$
ZC3H12C	chr11:109964087-110042566	1.48	$8.39x10^{-64}$
PAPLN	chr14:73704205-73741348	0.871	$1.06x10^{-63}$
ATP6V1B2	chr8:20054878-20084330	1.17	$3.60x10^{-63}$
STX4	chr16:31044210-31054296	1.57	$1.01x10^{-62}$
SLC4A8	chr12:51785101-51902980	1.18	$1.37x10^{-61}$
ZNF385A	chr12:54762917-54785082	0.906	$2.37x10^{-59}$
SNTB1	chr8:121547985-121825513	1.78	$1.33x10^{-57}$
GUSB	chr7:65425671-65447301	1.13	$2.47x10^{-57}$
PBX4	chr19:19672522-19729725	1.00	$1.00x10^{-56}$
KIAA0930	chr22:45586219-45636650	1.40	$3.58x10^{-54}$
BAIAP2	chr17:79008948-79091232	0.849	$6.96x10^{-54}$
NFKBID	chr19:36378555-36393205	0.878	$1.52x10^{-53}$
CD109	chr6:74405508-74538040	1.29	$4.19x10^{-53}$
AKR1A1	chr1:46016215-46035721	0.922	$7.01x10^{-53}$
PLIN2	chr9:19108373-19149288	1.46	$1.58x10^{-51}$
PLEKHM2	chr1:16010827-16061264	1.40	$2.75x10^{-51}$
RAB20	chr13:111175417-111214080	1.44	$7.03x10^{-51}$
FDX1	chr11:110300607-110335605	1.11	$7.32x10^{-51}$
SLC17A5	chr6:74303102-74363878	1.15	$8.27x10^{-51}$
CTSH	chr15:79213400-79241916	0.903	$1.01x10^{-50}$
LPAR2	chr19:19734477-19739739	0.739	$1.36x10^{-50}$
SETDB2	chr13:50018429-50069138	1.02	$2.45x10^{-50}$
TYROBP	chr19:36395303-36399197	1.00	$3.54x10^{-50}$
MADD	chr11:47290712-47351582	1.66	$1.15x10^{-49}$
LTA4H	chr12:96394606-96437298	0.983	$1.87x10^{-49}$

TRPV2	chr17:16318856-16340317	0.824	4.43×10^{-49}
CD9	chr12:6308881-6347425	0.954	9.04×10^{-49}
GGA1	chr22:38004481-38029571	1.27	9.92×10^{-48}
RGCC	chr13:42031695-42045018	1.68	4.25×10^{-47}
ZYX	chr7:143078173-143088204	0.928	1.68×10^{-46}
SIRPA	chr20:1875154-1920543	0.880	1.82×10^{-46}
PKM	chr15:72491370-72524164	0.989	1.65×10^{-44}
NAGK	chr2:71291474-71306935	0.994	1.68×10^{-44}
DOK3	chr5:176928908-176938275	0.689	6.64×10^{-44}
IL18	chr11:112013974-112034840	0.898	7.55×10^{-43}
PNPLA6	chr19:7598890-7626650	0.967	5.45×10^{-42}
ADAM9	chr8:38854388-38962663	1.13	1.30×10^{-41}
SLC36A1	chr5:150816607-150871942	0.896	1.54×10^{-41}
GCNT2	chr6:10492456-10629601	0.836	3.47×10^{-41}
HM13	chr20:30102231-30157370	1.26	1.35×10^{-40}
KCTD7	chr7:66093868-66276446	1.30	1.47×10^{-40}
RRN3P1	chr16:21807951-21831731	0.857	6.49×10^{-40}
NPC2	chr14:74942895-74960880	0.571	1.16×10^{-39}
ZNF267	chr16:31885079-31928668	0.657	1.2810^{-39}
TM6SF1	chr15:83776159-83813606	0.728	3.30×10^{-39}
SH3BGRL3	chr1:26605667-26608007	0.923	6.53×10^{-39}
ASAH1	chr8:17913934-17942494	1.13	1.62×10^{-38}
ARMC9	chr2:232063260-232239548	1.12	3.04×10^{-38}
APOE	chr19:45409011-45412650	1.88	3.30×10^{-38}
TNFRSF14	chr1:2487078-2496821	1.08	4.22×10^{-38}
LGALS3	chr14:55590828-55612126	1.35	1.39×10^{-37}
LPXN	chr11:58294344-58345693	0.815	2.27×10^{-36}
TMEM106A	chr17:41363854-41372061	0.753	5.41×10^{-36}
UNC93B1	chr11:67758575-67772452	0.813	9.82×10^{-36}
PLEK	chr2:68592305-68624585	1.03	2.94×10^{-35}
DENND4B	chr1:153901977-153919172	0.954	4.27×10^{-35}
TPM4	chr19:16177831-16213813	1.27	1.67×10^{-33}
FGD6	chr12:95470525-95611258	0.935	1.91×10^{-33}
CORO1C	chr12:109038885-109125372	1.10	2.47×10^{-33}
TTC39B	chr9:15163620-15307358	0.972	1.32×10^{-32}
DOT1L	chr19:2164148-2232577	1.23	2.29×10^{-32}
GPRIN3	chr4:90157537-90229161	0.896	3.43×10^{-32}
PABPC4	chr1:40026488-40042462	0.864	3.95×10^{-32}
ITGB2	chr21:46305868-46351904	0.753	4.85×10^{-32}
ABHD12	chr20:25275379-25371619	1.27	6.24×10^{-32}
MFHAS1	chr8:8640864-8751155	1.05	1.45×10^{-31}
AMD1	chr6:111195973-111216916	0.736	3.79×10^{-31}
AGAP3	chr7:150782918-150841523	0.897	8.22×10^{-31}
RIPK2	chr8:90769975-90803291	0.672	1.41×10^{-30}
C12orf5	chr12:4430371-4462338	0.670	1.82×10^{-30}
SLC11A2	chr12:51373184-51422349	0.843	2.32×10^{-30}

SLC8B1	chr12:113736564-113797298	0.767	$4.14x10^{-30}$
UBTD1	chr10:99258625-99330966	1.21	$1.88x10^{-29}$
GRN	chr17:42422614-42430470	1.00	$4.70x10^{-29}$
LY96	chr8:74903587-74941322	0.484	$7.05x10^{-29}$
DPEP2	chr16:68021297-68034489	0.880	$7.83x10^{-29}$
SCARB2	chr4:77079890-77135046	1.11	$1.35x10^{-28}$
NPC1	chr18:21086148-21166862	0.945	$1.72x10^{-28}$
CTTNBP2NL	chr1:112938803-113006078	0.844	$1.93x10^{-28}$
ST3GAL6	chr3:98451080-98540045	0.976	$2.46x10^{-28}$
ZFYVE26	chr14:68194091-68283307	0.731	$4.14x10^{-28}$
PTPN1	chr20:49126891-49201299	0.932	$5.69x10^{-28}$
DHX34	chr19:47852538-47885961	0.798	$1.05x10^{-27}$
ATP2A2	chr12:110718561-110788898	0.912	$2.93x10^{-27}$
SLC31A1	chr9:115983808-116028674	0.732	$1.78x10^{-26}$
SLC2A9	chr4:9772777-10056560	0.934	$2.83x10^{-26}$
GNA13	chr17:63006833-63052957	0.857	$4.92x10^{-26}$
B4GALT5	chr20:48249482-48330415	1.27	$6.10x10^{-26}$
LACTB	chr15:63413999-63434260	0.666	$6.74x10^{-26}$
GPR137B	chr1:236305832-236385165	0.573	$1.54x10^{-25}$
APLP2	chr11:129939732-130014699	0.610	$1.16x10^{-24}$
RP11-63P12.6	chr9:74920346-74958126	0.590	$1.58x10^{-24}$
SORBS3	chr8:22402499-22433301	0.601	$2.21x10^{-24}$
UCP2	chr11:73685712-73694352	0.761	$1.53x10^{-23}$
SDHAP1	chr3:195686619-195717189	0.664	$1.77x10^{-23}$
ME2	chr18:48405419-48474691	0.677	$2.43x10^{-23}$
PAQR8	chr6:52226219-52272575	0.675	$3.22x10^{-23}$
ANPEP	chr15:90328120-90358633	0.713	$3.99x10^{-23}$
BANP	chr16:87982850-88110924	0.791	$9.26x10^{-23}$
DCAF7	chr17:61627822-61671639	0.666	$1.94x10^{-22}$
CLCN7	chr16:1494935-1525581	0.908	$2.26x10^{-22}$
ZFYVE16	chr5:79703832-79775169	0.892	$3.00x10^{-22}$
MAN2B1	chr19:12757325-12777556	0.747	$3.99x10^{-22}$
GPD2	chr2:157291802-157470247	0.754	$6.69x10^{-22}$
SLC25A24	chr1:108676658-108743471	0.805	$7.40x10^{-22}$
HPS3	chr3:148847371-148891519	0.733	$9.59x10^{-22}$
TESK2	chr1:45809555-45956872	0.700	$1.03x10^{-21}$
LAMP1	chr13:113951556-113977987	0.700	$1.79x10^{-21}$
LPCAT2	chr16:55542910-55620582	0.518	$2.26x10^{-21}$
ENO1	chr1:8921061-8939308	0.642	$3.98x10^{-21}$
ADPGK	chr15:73043710-73078187	0.709	$4.21x10^{-21}$
LHFPL2	chr5:77781038-78065844	0.675	$5.58x10^{-21}$
IVNS1ABP	chr1:185265520-185286461	0.475	$1.43x10^{-20}$
GLUL	chr1:182350839-182361341	0.869	$1.60x10^{-20}$
UGCG	chr9:114659046-114697649	0.566	$1.97x10^{-20}$
GGA3	chr17:73232694-73258444	0.490	$2.95x10^{-20}$
SERTAD2	chr2:64858755-64978139	0.724	$3.20x10^{-20}$

HPS1	chr10:100175955-100206684	0.470	7.18×10^{-20}
SUSD1	chr9:114803065-114937688	0.591	1.03×10^{-19}
E2F3	chr6:20402398-20493941	0.547	1.05×10^{-19}
HK1	chr10:71029740-71161638	0.624	1.14×10^{-19}
ATP6V1A	chr3:113465866-113530903	0.899	1.99×10^{-19}
ANAPC7	chr12:110810705-110841535	0.461	2.01×10^{-19}
PSMA7	chr20:60711791-60718496	0.456	3.44×10^{-19}
CNIH3	chr1:224622362-224928251	1.26	3.50×10^{-19}
ARPC1B	chr7:98971872-98992424	0.588	1.12×10^{-18}
TBC1D9B	chr5:179289066-179334859	0.567	1.36×10^{-18}
NBEAL2	chr3:47021173-47051193	0.594	1.62×10^{-18}
ABHD2	chr15:89630690-89745591	0.681	1.63×10^{-18}
ST3GAL5	chr2:86066267-86116137	0.568	1.72×10^{-18}
RAB8B	chr15:63481668-63559981	0.700	2.27×10^{-18}
ATP6V1H	chr8:54628117-54756118	0.750	2.85×10^{-18}
ARL8B	chr3:5163905-5222596	0.659	5.15×10^{-18}
VAC14	chr16:70721342-70835064	1.01	6.18×10^{-18}
GNS	chr12:65107225-65153227	0.491	7.76×10^{-18}
AGO1	chr1:36335409-36395211	0.724	8.15×10^{-18}
MAST2	chr1:46252659-46501796	0.747	8.64×10^{-18}
ATP6V0D1	chr16:67471917-67515140	0.623	1.01×10^{-17}
CTD-2006C1.2	chr19:12098432-12157090	0.568	1.12×10^{-17}
SMS	chrX:21958691-22025798	0.664	1.38×10^{-17}
CD276	chr15:73976307-74006859	0.713	1.51×10^{-17}
MROH1	chr8:145202919-145316843	0.750	1.55×10^{-17}
ATP6V0B	chr1:44440159-44443967	0.602	2.10×10^{-17}
DEPDC5	chr22:32149944-32303012	0.577	2.48×10^{-17}
EFHD2	chr1:15736391-15756839	0.391	2.54×10^{-17}
VASP	chr19:46009837-46030241	0.366	2.97×10^{-17}
SNX1	chr15:64386322-64438289	0.849	3.76×10^{-17}
FBXO38	chr5:147763498-147822399	0.601	4.26×10^{-17}
PDXDC1	chr16:15068448-15233196	0.715	5.43×10^{-17}
C16orf70	chr16:67143861-67182442	0.509	5.94×10^{-17}
ANKRD6	chr6:90142889-90343553	0.611	6.37×10^{-17}
S100A11	chr1:152004982-152020383	0.484	8.05×10^{-17}
RHBDF2	chr17:74466973-74497872	0.700	9.14×10^{-17}
SRSF7	chr2:38970741-38978636	0.646	1.59×10^{-16}
SPATS2	chr12:49760367-49921205	0.552	2.18×10^{-16}
PITPNA	chr17:1421012-1466110	0.751	2.24×10^{-16}
REL	chr2:61108656-61158745	1.01	2.71×10^{-16}
ACTN1	chr14:69340860-69446157	0.763	4.76×10^{-16}
TRIM14	chr9:100831557-100881494	0.319	5.70×10^{-16}
TPM3	chr1:154127784-154167124	0.450	6.08×10^{-16}
CAP1	chr1:40505905-40538321	0.404	6.22×10^{-16}
TPD52L2	chr20:62496596-62522898	0.531	1.32×10^{-15}
TMEM181	chr6:158957468-159056460	0.480	1.47×10^{-15}

PCYT1A	chr3:195941093-196014828	0.994	$1.61x10^{-15}$
CTA-217C2.1	chr22:45528901-45559662	0.657	$1.70x10^{-15}$
ZCCHC2	chr18:60190240-60254942	0.659	$1.94x10^{-15}$
SPIRE1	chr18:12446511-12658133	0.731	$2.72x10^{-15}$
MAPK6	chr15:52244303-52358462	0.643	$3.30x10^{-15}$
WNT2B	chr1:113009163-113072787	0.685	$3.47x10^{-15}$
SPG21	chr15:65255362-65282648	0.514	$3.69x10^{-15}$
OSBPL11	chr3:125247702-125313934	0.640	$4.07x10^{-15}$
GNB4	chr3:179116990-179169378	0.433	$4.25x10^{-15}$
DNMT1	chr19:10244021-10341962	0.490	$5.67x10^{-15}$
VPS26A	chr10:70883268-70932617	0.364	$5.89x10^{-15}$
ZBTB43	chr9:129567285-129600489	0.543	$6.45x10^{-15}$
MIR24-2	chr19:13945330-13947173	0.453	$8.44x10^{-15}$
HGS	chr17:79650356-79670168	0.481	$8.99x10^{-15}$
RP11-106M3.2	chr15:72577068-72668322	0.919	$9.15x10^{-15}$
HNRNPLL	chr2:38789120-38830728	0.836	$1.02x10^{-14}$
ARHGDIA	chr17:79825597-79829282	0.368	$1.09x10^{-14}$
PLXNB2	chr22:50713408-50746056	0.549	$1.16x10^{-14}$
SNX10	chr7:26331541-26413949	0.466	$1.53x10^{-14}$
MIR181A1HG	chr1:198776622-198906558	0.623	$1.95x10^{-14}$
RGS12	chr4:3294755-3441640	0.698	$3.32x10^{-14}$
HCLS1	chr3:121350246-121379774	0.365	$4.52x10^{-14}$
IBTK	chr6:82879700-82957471	0.509	$5.99x10^{-14}$
INPPL1	chr11:71934745-71950149	0.522	$6.32x10^{-14}$
ARSB	chr5:78073032-78281910	0.447	$6.42x10^{-14}$
RALA	chr7:39663082-39747723	0.656	$7.28x10^{-14}$
SIPA1L2	chr1:232533711-232697304	0.547	$7.64x10^{-14}$
NEK6	chr9:127019885-127115586	0.728	$8.35x10^{-14}$
DTNBP1	chr6:15523032-15663289	0.368	$9.41x10^{-14}$
HIF1A	chr14:62162231-62214976	0.601	$1.01x10^{-13}$
VPS41	chr7:38762563-38971994	0.545	$1.16x10^{-13}$
SNX8	chr7:2291405-2393953	0.669	$1.25x10^{-13}$
SLC23A2	chr20:4833002-4990939	0.494	$2.07x10^{-13}$
TEP1	chr14:20833826-20881588	0.363	$2.40x10^{-13}$
PAX8-AS1	chr2:113969099-114034158	0.460	$4.42x10^{-13}$
CMTM7	chr3:32433163-32524559	0.469	$4.60x10^{-13}$
TDG	chr12:104359582-104382652	0.507	$5.37x10^{-13}$
ATP6V1C1	chr8:104033291-104085279	0.555	$5.87x10^{-13}$
DGKZ	chr11:46354455-46402104	0.782	$6.41x10^{-13}$
CD53	chr1:111415775-111442550	0.284	$8.92x10^{-13}$
STX2	chr12:131274145-131323811	0.737	$1.03x10^{-12}$
SLC41A2	chr12:105196331-105352522	0.744	$1.33x10^{-12}$
CAMSAP1	chr9:138700333-138799074	0.588	$1.58x10^{-12}$
ESRRA	chr11:64073044-64084215	0.408	$1.70x10^{-12}$
CD58	chr1:117057157-117113661	0.591	$2.46x10^{-12}$
SYNJ1	chr21:34001069-34100359	0.541	$2.58x10^{-12}$

EIF3A	chr10:120794356-120840316	0.441	$2.70x10^{-12}$
MCL1	chr1:150547032-150552066	0.594	$2.95x10^{-12}$
BRI3	chr7:97881691-97937162	0.498	$3.04x10^{-12}$
SNX24	chr5:122179134-122365049	0.407	$4.43x10^{-12}$
NBPF1	chr1:16888814-16940057	0.604	$5.32x10^{-12}$
PAPSS1	chr4:108511433-108641608	0.592	$5.39x10^{-12}$
KIAA0100	chr17:26941458-26972472	0.486	$5.60x10^{-12}$
ZFAND5	chr9:74966341-74980163	0.407	$7.74x10^{-12}$
ANXA11	chr10:81910645-81965328	0.809	$1.42x10^{-11}$
MDM2	chr12:69201956-69239214	0.725	$1.79x10^{-11}$
ERN1	chr17:62116502-62208179	0.317	$1.95x10^{-11}$
ARHGEF11	chr1:156904632-157015162	0.621	$1.97x10^{-11}$
ACBD3	chr1:226332380-226374431	0.657	$2.42x10^{-11}$
ATP1B3	chr3:141594966-141645356	0.400	$2.80x10^{-11}$
NSMAF	chr8:59496063-59572403	0.520	$5.12x10^{-11}$
SEC24D	chr4:119643978-119759838	0.264	$5.18x10^{-11}$
ACOT9	chrX:23720370-23784592	0.469	$5.47x10^{-11}$
OAZ1	chr19:2269485-2273487	0.570	$7.00x10^{-11}$
ARFGAP3	chr22:43192508-43254112	0.469	$8.33x10^{-11}$
TANGO2	chr22:20004537-20053449	0.466	$9.62x10^{-11}$
GPX1	chr3:49394609-49396033	0.338	$1.13x10^{-10}$
LASP1	chr17:37026112-37078023	0.530	$1.41x10^{-10}$
LYPLA1	chr8:54958938-55014577	0.339	$1.64x10^{-10}$
TXNDC11	chr16:11772936-11836734	0.330	$1.96x10^{-10}$
ATG4C	chr1:63249806-63331184	0.450	$1.98x10^{-10}$
RHEB	chr7:151163098-151217206	0.471	$2.27x10^{-10}$
TMOD3	chr15:52121825-52239492	0.536	$2.56x10^{-10}$
EHD4	chr15:42190950-42264776	0.479	$2.74x10^{-10}$
FIG4	chr6:110012499-110146631	0.473	$2.81x10^{-10}$
TRAF5	chr1:211499957-211548288	0.446	$3.01x10^{-10}$
KPNA4	chr3:160212783-160283376	0.362	$3.05x10^{-10}$
GATAD2A	chr19:19496635-19619740	0.598	$3.44x10^{-10}$
MGRN1	chr16:4666494-4740975	0.471	$4.23x10^{-10}$
FGGY	chr1:59762310-60233347	0.457	$4.40x10^{-10}$
PRKX	chrX:3522411-3631649	0.251	$4.45x10^{-10}$
VPS29	chr12:110928902-110939922	0.448	$4.99x10^{-10}$
TMEM63A	chr1:226033237-226070069	0.538	$7.89x10^{-10}$
TSC22D1	chr13:45007655-45151283	0.365	$8.24x10^{-10}$
OSTF1	chr9:77703459-77762181	0.279	$1.03x10^{-9}$
PI4KB	chr1:151264273-151300191	0.521	$1.04x10^{-9}$
ABL2	chr1:179068462-179198819	0.416	$1.52x10^{-9}$
SZT2	chr1:43855553-43918321	0.431	$1.55x10^{-9}$
TUBGCP2	chr10:135093135-135125841	0.294	$1.71x10^{-9}$
SDCBP	chr8:59465483-59495419	0.502	$1.91x10^{-9}$
MME	chr3:154741913-154901497	1.05	$1.98x10^{-9}$
AP1B1	chr22:29723669-29819168	0.390	$2.10x10^{-9}$

BTBD7	chr14:93703896-93799438	0.381	$3.47x10^{-9}$
RNF145	chr5:158584417-158637061	0.348	$3.82x10^{-9}$
STX18	chr4:4417469-4544073	0.375	$4.07x10^{-9}$
INF2	chr14:105155943-105185942	0.561	$4.51x10^{-9}$
WDFY1	chr2:224720433-224810104	0.553	$6.02x10^{-9}$
GPR107	chr9:132815705-132902448	0.434	$6.87x10^{-9}$
CTD-2547E10.2	chr16:21458004-21531765	0.420	$6.97x10^{-9}$
SNORA76.2	chr5:65257011-65257147	0.394	$7.64x10^{-9}$
ANXA5	chr4:122589110-122618268	0.340	$8.24x10^{-9}$
RHOQ	chr2:46768945-46810260	0.545	$8.32x10^{-9}$
CEBPB	chr20:48807376-48809212	0.363	$9.26x10^{-9}$
CSNK1D	chr17:80196899-80231607	0.438	$9.44x10^{-9}$
EP400	chr12:132434508-132565005	0.285	$9.44x10^{-9}$
RNH1	chr11:494512-507300	0.472	$1.03x10^{-8}$
VAPA	chr18:9913999-9960018	0.424	$1.08x10^{-8}$
BTBD1	chr15:83685174-83736106	0.363	$1.16x10^{-8}$
HADHA	chr2:26413504-26467594	0.312	$1.25x10^{-8}$
ATG4B	chr2:242576628-242613272	0.401	$1.37x10^{-8}$
C6orf62	chr6:24705089-24721064	0.307	$1.43x10^{-8}$
ZDHHC7	chr16:85007787-85045141	0.413	$1.50x10^{-8}$
NFKB1	chr4:103422486-103538459	0.415	$1.53x10^{-8}$
PPIL2	chr22:22006559-22054304	0.371	$1.59x10^{-8}$
ST3GAL2	chr16:70413338-70473140	0.442	$1.95x10^{-8}$
CLTA	chr9:36190853-36304778	0.496	$2.13x10^{-8}$
ATXN2L	chr16:28834356-28848558	0.412	$2.31x10^{-8}$
RAB6A	chr11:73386683-73472182	0.302	$2.83x10^{-8}$
LRSAM1	chr9:130213765-130265780	0.577	$3.67x10^{-8}$
USP12	chr13:27640293-27746033	0.409	$5.78x10^{-8}$
SQSTM1	chr5:179233388-179265078	0.797	$9.22x10^{-8}$
RDX	chr11:110045605-110167447	0.420	$1.10x10^{-7}$
SH3GL1	chr19:4360367-4400544	0.294	$1.42x10^{-7}$
CCDC93	chr2:118673054-118771709	0.398	$1.49x10^{-7}$
CUEDC1	chr17:55938604-56032684	0.424	$1.51x10^{-7}$
EIF2AK4	chr15:40226347-40327797	0.382	$1.56x10^{-7}$
NAA50	chr3:113435307-113465147	0.402	$1.85x10^{-7}$
XRN2	chr20:21283942-21370463	0.322	$1.92x10^{-7}$
SUPT16H	chr14:21819631-21852425	0.303	$2.24x10^{-7}$
SNX29P2	chr16:29362067-29381593	0.379	$2.78x10^{-7}$
SPPL2A	chr15:50999506-51058005	0.427	$3.03x10^{-7}$
SETD3	chr14:99864083-99947216	0.311	$3.22x10^{-7}$
GGA2	chr16:23474863-23533316	0.314	$3.36x10^{-7}$
CYB5R4	chr6:84569362-84677143	0.262	$3.51x10^{-7}$
TAF1D	chr11:93463114-93517557	0.411	$4.67x10^{-7}$
CAPZA2	chr7:116451124-116562103	0.346	$4.75x10^{-7}$
RNF19A	chr8:101269288-101348446	0.554	$4.95x10^{-7}$
L3MBTL4	chr18:5954705-6415236	0.657	$5.10x10^{-7}$

SLC43A2	chr17:1472561-1532180	0.609	$5.13x10^{-7}$
PTMA	chr2:232571605-232578251	0.320	$5.23x10^{-7}$
RAD51B	chr14:68286496-69196935	0.511	$6.18x10^{-7}$
COLGALT1	chr19:17666403-17693971	0.339	$6.52x10^{-7}$
KPNB1	chr17:45726842-45762871	0.375	$7.54x10^{-7}$
TOM1	chr22:35695268-35743985	0.351	$7.60x10^{-7}$
ANKMY1	chr2:241418839-241508626	0.366	$9.05x10^{-7}$
EIF5	chr14:103799881-103811362	0.301	$1.06x10^{-6}$
IQSEC1	chr3:12938719-13114617	0.498	$1.07x10^{-6}$
CPEB4	chr5:173315283-173388979	0.290	$1.14x10^{-6}$
SRP54	chr14:35451163-35498773	0.271	$1.15x10^{-6}$
MTDH	chr8:98656407-98740998	0.272	$1.20x10^{-6}$
PSMD1	chr2:231921578-232037541	0.431	$1.25x10^{-6}$
AIM1	chr6:106959730-107018326	0.399	$1.27x10^{-6}$
GPBP1L1	chr1:46092976-46153785	0.409	$1.29x10^{-6}$
DNMT3A	chr2:25455845-25565459	0.294	$1.31x10^{-6}$
NIN	chr14:51186481-51297839	0.283	$1.48x10^{-6}$
TAOK1	chr17:27717482-27878922	0.393	$1.55x10^{-6}$
GNA12	chr7:2767746-2883958	0.352	$1.57x10^{-6}$
DRAM2	chr1:111659955-111682838	0.384	$1.59x10^{-6}$
MED15	chr22:20850200-20941919	0.251	$1.98x10^{-6}$
CHFR	chr12:133398773-133532890	0.295	$2.39x10^{-6}$
AP3D1	chr19:2100988-2164464	0.365	$3.07x10^{-6}$
EMP3	chr19:48824766-48833810	0.312	$3.63x10^{-6}$
WHSC1	chr4:1873151-1983934	0.289	$3.71x10^{-6}$
TMEM259	chr19:1009647-1021117	0.353	$4.23x10^{-6}$
ATP6V1D	chr14:67761088-67826982	0.356	$4.36x10^{-6}$
CUL2	chr10:35297479-35379570	0.288	$5.25x10^{-6}$
ETNK1	chr12:22778009-22843599	0.518	$6.22x10^{-6}$
COPA	chr1:160259063-160313190	0.314	$6.77x10^{-6}$
U2AF1	chr21:44513066-44527697	0.304	$7.14x10^{-6}$
HADHB	chr2:26466038-26513336	0.286	$7.82x10^{-6}$
KIAA0430	chr16:15688243-15737023	0.407	$8.84x10^{-6}$
TYK2	chr19:10461209-10491352	0.329	$9.45x10^{-6}$
AGO3	chr1:36396319-36538101	0.296	$1.34x10^{-5}$
YEATS2	chr3:183415606-183530413	0.344	$1.37x10^{-5}$
NFYC	chr1:41157320-41237275	0.323	$1.49x10^{-5}$
STK40	chr1:36805225-36851497	0.283	$1.82x10^{-5}$
SERF2	chr15:44069285-44094787	0.334	$2.11x10^{-5}$
PGS1	chr17:76374721-76421195	0.377	$2.42x10^{-5}$
MBOAT1	chr6:20100935-20212670	0.371	$2.70x10^{-5}$
MARC7	chr2:160569000-160627538	0.397	$3.13x10^{-5}$
TXNRD1	chr12:104609557-104744061	1.08	$3.18x10^{-5}$
ATP6V0E1	chr5:172410760-172462448	0.373	$4.09x10^{-5}$
SCYL2	chr12:100660918-100735502	0.332	$4.18x10^{-5}$
ASCC3	chr6:100956070-101329248	0.308	$4.54x10^{-5}$

TMEM62	chr15:43415477-43477344	0.330	$4.88x10^{-5}$
TANGO6	chr16:68877507-69119083	0.313	$5.28x10^{-5}$
P4HB	chr17:79801035-79818570	0.331	$5.68x10^{-5}$
CDK5RAP3	chr17:46045176-46059140	0.301	$5.71x10^{-5}$
SLC35F5	chr2:114462588-114514400	0.423	9.3110^{-5}
FOXK2	chr17:80477589-80602538	0.323	$9.72x10^{-5}$
SUGP2	chr19:19101697-19144832	0.376	$9.86x10^{-5}$
FBXO7	chr22:32870663-32894818	0.265	$1.02x10^{-4}$
ZSWIM8	chr10:75545340-75561551	0.286	$1.11x10^{-4}$
CCDC12	chr3:46963216-47023500	0.318	$1.23x10^{-4}$
XPC	chr3:14186647-14220283	0.363	$1.38x10^{-4}$
HTT	chr4:3076408-3245676	0.285	$1.57x10^{-4}$
SCFD1	chr14:31091318-31205018	0.315	$1.57x10^{-4}$
UBE2Q2	chr15:76135622-76193419	0.376	$1.75x10^{-4}$
INO80	chr15:41271078-41408552	0.258	$2.06x10^{-4}$
AGPS	chr2:178257372-178408564	0.273	$2.17x10^{-4}$
SNX14	chr6:86215214-86303874	0.514	$2.25x10^{-4}$
MTMR14	chr3:9691117-9744077	0.384	$2.37x10^{-4}$
KPNA3	chr13:50273447-50367057	0.286	$2.44x10^{-4}$
ANKH	chr5:14704910-14871887	0.283	$2.70x10^{-4}$
MDN1	chr6:90352218-90529442	0.328	$4.20x10^{-4}$
SEL1L	chr14:81937893-82000205	0.388	$4.33x10^{-4}$
ATF4	chr22:39915700-39918691	0.276	$4.63x10^{-4}$
WDR7	chr18:54318574-54698828	0.339	$5.18x10^{-4}$
VPS39	chr15:42450899-42500514	0.271	$6.61x10^{-4}$
ACIN1	chr14:23527773-23564823	0.264	$6.78x10^{-4}$
WIPI2	chr7:5229819-5273457	0.265	$7.39x10^{-4}$
SCFD2	chr4:53739149-54232242	0.305	$7.45x10^{-4}$
ERGIC1	chr5:172261278-172379688	0.342	$7.72x10^{-4}$
ARHGEF10L	chr1:17866330-18024369	0.266	$8.15x10^{-4}$
HEATR5A	chr14:31760994-31889788	0.338	$9.67x10^{-4}$
SSBP4	chr19:18529674-18545372	0.276	$1.16x10^{-3}$
HDLBP	chr2:242166679-242256476	0.346	$1.53x10^{-3}$
XPR1	chr1:180601140-180859387	0.280	$1.56x10^{-3}$
TMEM117	chr12:44229770-44783545	0.541	$1.60x10^{-3}$
TAF2	chr8:120743015-120845103	0.348	$1.60x10^{-3}$
UBA6	chr4:68478370-68566897	0.293	$1.96x10^{-3}$
WDR43	chr2:29117509-29171088	0.298	$2.00x10^{-3}$
FEZ2	chr2:36778570-36873230	0.313	$2.24x10^{-3}$
CYSTM1	chr5:139554227-139661637	0.441	$2.54x10^{-3}$
MAN2C1	chr15:75648133-75660971	0.277	$2.55x10^{-3}$
DTNB	chr2:25600067-25896503	0.390	$3.53x10^{-3}$
RNF216	chr7:5659678-5821370	0.259	$4.16x10^{-3}$
FAM120A	chr9:96214004-96328397	0.271	$4.54x10^{-3}$
ACO2	chr22:41865129-41924993	0.272	$5.05x10^{-3}$
CEP192	chr18:12991361-13125051	0.352	$5.28x10^{-3}$

	UBE2D3	chr4:103715540-103790053	0.300	5.98×10^{-3}
	CPEB3	chr10:93806449-94050844	0.276	7.38×10^{-3}
	IRF2	chr4:185308867-185395734	0.255	7.90×10^{-3}
	RIOK3	chr18:21032787-21066567	0.411	9.11×10^{-3}
	GOLGA3	chr12:133345495-133405444	0.291	0.0108
	MAP3K8	chr10:30722866-30750762	0.482	0.0112
	RPL37	chr5:40825364-40835437	0.289	0.0133
	ZBTB1	chr14:64970430-65000408	0.266	0.0133
	IPO9	chr1:201798269-201853422	0.279	0.0173
	MAPK8IP3	chr16:1756184-1820318	0.251	0.0192
	RAC1	chr7:6414154-6443608	0.340	0.0198
	CDYL	chr6:4706393-4955785	0.304	0.0360
	MARC2	chr19:8478154-8503901	0.277	0.0381
	TANC2	chr17:61086917-61505060	0.384	0.0384
	INTS10	chr8:19674651-19709594	0.293	0.0446
Mast cells	AC004791.2	chr19:15962803-15975714	4.43	$<2.23 \times 10^{-308}$
	CPA3	chr3:148583043-148614983	4.00	$<2.23 \times 10^{-308}$
	HPGD	chr4:175411328-175444305	3.87	$<2.23 \times 10^{-308}$
	KIT	chr4:55524085-55606881	3.80	$<2.23 \times 10^{-308}$
	RAB27B	chr18:52385091-52562747	3.67	$<2.23 \times 10^{-308}$
	IL18R1	chr2:102927989-103015218	3.55	$<2.23 \times 10^{-308}$
	TPSB2	chr16:1277272-1280214	3.53	$<2.23 \times 10^{-308}$
	SLC8A3	chr14:70510934-70655787	3.48	$<2.23 \times 10^{-308}$
	HDC	chr15:50534144-50558223	3.43	$<2.23 \times 10^{-308}$
	STX3	chr11:59480929-59573354	3.42	$<2.23 \times 10^{-308}$
	CDK15	chr2:202655184-202760273	3.40	$<2.23 \times 10^{-308}$
	SLC18A2	chr10:119000604-119038941	3.32	$<2.23 \times 10^{-308}$
	BACE2	chr21:42539728-42654445	3.29	$<2.23 \times 10^{-308}$
	CTD-3179P9.1	chr5:117260703-117601730	3.25	$<2.23 \times 10^{-308}$
	SYTL3	chr6:159071046-159185908	3.12	$<2.23 \times 10^{-308}$
	ST8SIA1	chr12:22216707-22589975	3.06	$<2.23 \times 10^{-308}$
	VWA5A	chr11:123986069-124018428	2.98	$<2.23 \times 10^{-308}$
	HPGDS	chr4:95219686-95264027	2.97	$<2.23 \times 10^{-308}$
	P2RX1	chr17:3799886-3819794	2.95	$<2.23 \times 10^{-308}$
	RGS13	chr1:192605275-192629390	2.83	$<2.23 \times 10^{-308}$
	KIAA1549	chr7:138516126-138666064	2.83	$<2.23 \times 10^{-308}$
	TPSAB1	chr16:1290697-1292555	2.72	$<2.23 \times 10^{-308}$
	PPM1H	chr12:63037762-63328817	2.58	$<2.23 \times 10^{-308}$
	GATA2	chr3:128198270-128212028	2.57	$<2.23 \times 10^{-308}$
	AP1S3	chr2:224616403-224702744	2.51	$<2.23 \times 10^{-308}$
	ENPP3	chr6:131949582-132068553	2.50	$<2.23 \times 10^{-308}$
	MLPH	chr2:238394071-238463961	2.38	$<2.23 \times 10^{-308}$
	HS6ST1	chr2:128994290-129076151	2.36	$<2.23 \times 10^{-308}$
	PIK3R6	chr17:8706041-8770994	2.17	$<2.23 \times 10^{-308}$
	SYTL2	chr11:85405267-85522184	2.30	1.38×10^{-297}
	FAM129B	chr9:130267618-130341268	2.19	3.34×10^{-290}

	EXTL3	chr8:28457986-28613116	2.12	$2.75x10^{-224}$
	ATP6V0A2	chr12:124196865-124246302	2.06	$1.28x10^{-182}$
	CTNNBL1	chr20:36322408-36500531	2.05	$1.06x10^{-145}$
	STXBP5	chr6:147525561-147706866	2.12	$1.01x10^{-132}$
	MYO16	chr13:109248500-109860355	1.90	$1.18x10^{-87}$
	MARC3	chr5:126203406-126366500	1.74	$9.39x10^{-65}$
	DNAJC1	chr10:22045466-22292698	1.14	$3.28x10^{-15}$
	RFX3	chr9:3218297-3526004	1.01	$5.38x10^{-9}$
	FAM222B	chr17:27082996-27182250	0.967	$5.49x10^{-7}$
	NBAS	chr2:15307032-15701454	0.848	$1.48x10^{-4}$
	CLASP2	chr3:33537737-33759848	0.787	$9.78x10^{-3}$
Monocytes	FCN1	chr9:137801431-137809809	2.65	$<2.23x10^{-308}$
	CLEC12A	chr12:10103915-10148293	2.36	$<2.23x10^{-308}$
	CLEC7A	chr12:10269376-10282857	1.70	$<2.23x10^{-308}$
	NAAA	chr4:76831809-76862204	1.26	$8.29x10^{-88}$
	C10orf54	chr10:73507316-73533255	1.30	$7.40x10^{-78}$
	WARS	chr14:100800125-100843142	1.31	$1.36x10^{-74}$
	ATG16L2	chr11:72525353-72554719	1.14	$6.80x10^{-54}$
	ANKRD13D	chr11:67056018-67069956	1.20	$2.93x10^{-51}$
	FGD3	chr9:95709733-95798518	0.887	$5.18x10^{-46}$
	ADRBK1	chr11:67033881-67054027	0.991	$4.30x10^{-33}$
	SIK3	chr11:116714118-116969153	0.724	$1.81x10^{-17}$
	CHD1	chr5:98190908-98262240	0.696	$2.43x10^{-12}$
	MSN	chrX:64808257-64961791	0.648	$6.64x10^{-11}$
	C1orf63	chr1:25568728-25664704	0.646	$8.00x10^{-10}$
	ZNF106	chr15:42705021-42783321	0.708	$5.01x10^{-8}$
	FBXW7	chr4:153242410-153457253	0.551	$2.03x10^{-6}$
	MBD2	chr18:51679079-51751158	0.764	$2.48x10^{-4}$
	KMT2C	chr7:151832010-152133090	0.428	$4.29x10^{-4}$
	R3HDM2	chr12:57643392-57824788	0.608	$6.41x10^{-4}$
	ERICH1	chr8:564746-688106	0.574	$7.64x10^{-3}$
	SMARCC1	chr3:47626762-47823596	0.474	0.019201696
	BIRC6	chr2:32582096-32843966	0.394	0.040919656
Myeloid 1	EIF2B5	chr3:183852826-184402546	2.06	$<2.23x10^{-308}$
	MPPED2	chr11:30406040-30608419	1.99	$<2.23x10^{-308}$
	RYR1	chr19:38924339-39078204	1.95	$<2.23x10^{-308}$
	GGTA1P	chr9:124207269-124262306	1.91	$<2.23x10^{-308}$
	CCDC141	chr2:179694484-179914813	1.84	$<2.23x10^{-308}$
	WLS	chr1:68564142-68698803	1.67	$<2.23x10^{-308}$
	CMKLR1	chr12:108681821-108733118	1.61	$<2.23x10^{-308}$
	CTSC	chr11:88026760-88070955	1.51	$<2.23x10^{-308}$
	CSGALNACT1	chr8:19261672-19615540	1.50	$<2.23x10^{-308}$
	HECTD2	chr10:93170096-93274586	1.23	$<2.23x10^{-308}$
	PIGX	chr3:196366646-196462878	1.21	$<2.23x10^{-308}$
	GLIS3	chr9:3824127-4348392	1.28	$1.60x10^{-279}$
	TCF12	chr15:57210821-57591479	0.943	$1.61x10^{-258}$

	DIRC2	chr3:122513642-122599986	1.08	$4.33x10^{-241}$
	TMCC1	chr3:129366635-129612419	1.01	$6.49x10^{-219}$
	C2CD5	chr12:22601517-22697480	0.977	$5.81x10^{-181}$
	NCOA1	chr2:24714783-24993571	0.638	$4.57x10^{-113}$
	ARID3A	chr19:925781-975939	0.896	$2.14x10^{-112}$
	TBC1D5	chr3:17198654-18486309	0.644	$8.04x10^{-112}$
	FKBP5	chr6:35541362-35696360	0.703	$7.16x10^{-85}$
	CCDC91	chr12:28286182-28732883	0.619	$2.99x10^{-78}$
	KLF7	chr2:207938861-208031991	0.618	$1.21x10^{-71}$
	SESN1	chr6:109307640-109416022	0.719	$9.89x10^{-70}$
	PHKB	chr16:47495034-47735434	0.611	$8.23x10^{-65}$
	RERE	chr1:8412457-8877702	0.462	$1.81x10^{-63}$
	OXR1	chr8:107282473-107764922	0.730	$5.09x10^{-60}$
	NUBPL	chr14:31959162-32330430	0.744	$4.07x10^{-56}$
	ZNF644	chr1:91380859-91487829	0.577	$1.92x10^{-46}$
	AGFG1	chr2:228336868-228425930	0.585	$1.50x10^{-45}$
	COMMD10	chr5:115420688-115748459	0.536	$2.06x10^{-38}$
	COG5	chr7:106842000-107204959	0.452	$1.37x10^{-36}$
	WDR70	chr5:37379314-37753537	0.469	$1.62x10^{-33}$
	DYRK1A	chr21:38738092-38889753	0.410	$6.36x10^{-28}$
	GOLGA4	chr3:37284668-37408242	0.379	$2.96x10^{-27}$
	KIAA1432	chr9:5629025-5776557	0.465	$1.51x10^{-21}$
	NIPBL	chr5:36876861-37066515	0.307	$8.55x10^{-21}$
	FUS	chr16:31191431-31203127	0.325	$9.97x10^{-19}$
	ATF7IP	chr12:14518610-14651697	0.355	$2.96x10^{-15}$
	USP9X	chrX:40944888-41095832	0.374	$1.27x10^{-14}$
	NR3C1	chr5:142657496-142815077	0.262	$1.95x10^{-13}$
	PUM2	chr2:20448452-20551995	0.279	$5.29x10^{-13}$
	EXOC4	chr7:132937829-133751342	0.266	$9.78x10^{-13}$
	PDCD6IP	chr3:33839844-33911194	0.328	$6.69x10^{-8}$
	GSK3B	chr3:119540170-119813264	0.270	$1.11x10^{-6}$
	MLLT10	chr10:21823094-22032559	0.272	$3.34x10^{-6}$
	POU2F1	chr1:167190066-167396582	0.347	$1.28x10^{-5}$
	ANKRD12	chr18:9136226-9285983	0.273	$4.67x10^{-3}$
Myeloid 3	PRKAG2	chr7:151253197-151574210	0.502	$1.40x10^{-17}$
	MAP2K1	chr15:66679155-66784650	0.536	$2.54x10^{-15}$
	RAP1GDS1	chr4:99182535-99365012	0.319	$2.13x10^{-11}$
	KMT2E	chr7:104654626-104754808	0.364	$2.98x10^{-10}$
	ASXL2	chr2:25956622-26101385	0.351	$4.37x10^{-10}$
	ST3GAL1	chr8:134467091-134584183	0.374	$8.43x10^{-10}$
	TCF25	chr16:89940000-89977792	0.327	$1.70x10^{-9}$
	CHD2	chr15:93426526-93571237	0.357	$1.18x10^{-8}$
	CACUL1	chr10:120433679-120514761	0.398	$4.60x10^{-8}$
	HEATR5B	chr2:37195526-37311485	0.372	$2.74x10^{-7}$
	NUFIP2	chr17:27582854-27621136	0.319	$1.04x10^{-6}$
	ARID1A	chr1:27022524-27108595	0.305	$2.93x10^{-6}$

	PRR14L	chr22:32072242-32146126	0.313	$2.68x10^{-5}$
	EP300	chr22:41487790-41576081	0.262	$2.11x10^{-4}$
	TRAPPC9	chr8:140742586-141468678	0.279	$3.28x10^{-4}$
	ARFGEF2	chr20:47538427-47653230	0.253	$8.56x10^{-4}$
	STX8	chr17:9153788-9479908	0.250	$1.21x10^{-3}$
	SP3	chr2:174771187-174830430	0.273	$170x10^{-3}$
	MDM4	chr1:204485511-204542871	0.302	$1.71x10^{-3}$
	TFCP2	chr12:51487446-51566926	0.253	$2.85x10^{-3}$
	CSNK1A1	chr5:148871760-148931007	0.254	$3.82x10^{-3}$
	ITFG1	chr16:47188298-47498060	0.262	0.0148
	ARFGEF1	chr8:68085747-68255912	0.255	0.0242
Myeloid 4	C1QA	chr1:22962999-22966101	2.05	$5.42x10^{-278}$
	C1QC	chr1:22970123-22974603	1.86	$2.34x10^{-264}$
	VSIG4	chrX:65241580-65259967	1.45	$1.51x10^{-160}$
	MAFB	chr20:39314488-39317880	1.46	$2.08x10^{-110}$
	TAGLN2	chr1:159887897-159895522	1.15	$9.60x10^{-70}$
	TPT1	chr13:45911008-45915505	1.09	$1.02x10^{-40}$
	S100A6	chr1:153507075-153508720	1.04	$8.03x10^{-40}$
	RPL37A	chr2:217362912-217443903	0.917	$5.43x10^{-23}$
	S100A10	chr1:151955391-151966866	0.865	$5.29x10^{-19}$
	RPLP2	chr11:809647-812880	0.780	$1.53x10^{-16}$
	RPL11	chr1:24018269-24022915	0.823	$2.63x10^{-14}$
	RPS24	chr10:79793518-79816570	0.858	$3.27x10^{-14}$
	MYL6	chr12:56551945-56557280	0.495	0.0317
Neutrophils	NAMPT	chr7:105888731-105926772	4.17	$<2.23x10^{-308}$
	KCNJ15	chr21:39529128-39679279	4.16	$<2.23x10^{-308}$
	GLT1D1	chr12:129337972-129469509	3.56	$<2.23x10^{-308}$
	LUCAT1	chr5:90598846-90610219	3.35	$<2.23x10^{-308}$
	TNFRSF10C	chr8:22941868-22974950	3.31	$<2.23x10^{-308}$
	TMEM154	chr4:153539784-153601317	3.08	$<2.23x10^{-308}$
	EPHB1	chr3:134316643-134979309	3.81	$5.93x10^{-255}$
	S100A8	chr1:153362508-153363664	2.93	$3.30x10^{-212}$
	RNF24	chr20:3907956-3996229	3.17	$2.09x10^{-198}$
	MXD1	chr2:70124820-70170077	2.96	$7.17x10^{-180}$
	S100A9	chr1:153330330-153333503	2.86	$1.59x10^{-165}$
	SLC25A37	chr8:23386318-23432976	3.13	$3.35x10^{-108}$
	LIMK2	chr22:31608225-31676066	2.52	$3.28x10^{-70}$
	XPO6	chr16:28109300-28223241	2.62	$1.40x10^{-69}$
	SOD2	chr6:160090089-160183561	1.87	$1.40x10^{-16}$
	UBR2	chr6:42531800-42661242	1.61	$2.43x10^{-10}$
	RICTOR	chr5:38938021-39074510	1.70	$3.98x10^{-9}$
	BRAF	chr7:140419127-140624564	0.343	$8.32x10^{-3}$
NK cells	GNLY	chr2:85912298-85925977	3.30	$<2.23x10^{-308}$
	PPP2R2B	chr5:145967936-146464347	2.11	$<2.23x10^{-308}$
	SPON2	chr4:1160720-1202750	1.96	$9.77x10^{-278}$
	MACF1	chr1:39546988-39952849	0.591	$2.84x10^{-13}$

	ITCH	chr20:32951041-33099198	0.765	1.89x10^{-4}
	GPBP1	chr5:56469775-56560506	0.250	4.05x10^{-4}
	ZRANB2	chr1:71528974-71546980	0.257	9.57x10^{-4}
	MPHOSPH8	chr13:20207788-20247599	0.262	2.02x10^{-3}
	PCM1	chr8:17780349-17885478	0.276	2.05x10^{-3}
	PPP6R2	chr22:50781733-50883514	0.293	3.23x10^{-3}
	HNRNPC	chr14:21677295-21737653	0.296	3.66x10^{-3}
	PRRC2B	chr9:134269480-134375584	0.282	7.42x10^{-3}
	CREBBP	chr16:3775055-3930727	0.260	0.0236
Perivascular	RGS6	chr14:72399156-73030654	4.00	<2.23x10^{-308}
	COL25A1	chr4:109731877-110223813	3.93	<2.23x10^{-308}
	KCNAB1	chr3:155755490-156256545	3.40	<2.23x10^{-308}
	MYO1B	chr2:192109911-192290115	2.93	<2.23x10^{-308}
	POSTN	chr13:38136720-38172981	2.46	<2.23x10^{-308}
	NR2F2-AS1	chr15:96670598-96870590	2.42	<2.23x10^{-308}
	FRMD3	chr9:85857905-86153461	2.36	<2.23x10^{-308}
	ENOX1	chr13:43787654-44361044	2.28	<2.23x10^{-308}
	NOTCH3	chr19:15270444-15311792	2.24	<2.23x10^{-308}
	IL1RAPL1	chrX:28605516-29974840	2.24	<2.23x10^{-308}
	DPY19L2	chr12:63952693-64062719	2.17	<2.23x10^{-308}
	CACNA1C	chr12:2079952-2802108	2.08	<2.23x10^{-308}
	TRPC6	chr11:101322295-101743293	2.07	<2.23x10^{-308}
	VIPR1	chr3:42530791-42579059	2.01	<2.23x10^{-308}
	COL18A1	chr21:46825052-46933634	1.96	<2.23x10^{-308}
	STEAP4	chr7:87905744-87936206	1.96	<2.23x10^{-308}
	BAI3	chr6:69345259-70099403	1.94	<2.23x10^{-308}
	SEMA5A	chr5:9035138-9546187	1.93	<2.23x10^{-308}
	CASC15	chr6:21665003-22214734	1.86	<2.23x10^{-308}
	LMOD1	chr1:201865580-201915715	1.85	<2.23x10^{-308}
	SYNPO2	chr4:119809996-119982402	1.82	<2.23x10^{-308}
	TBX2	chr17:59477257-59486827	1.81	<2.23x10^{-308}
	PDE1A	chr2:183004763-183387919	1.77	<2.23x10^{-308}
	ARHGEF17	chr11:73019334-73080136	1.76	<2.23x10^{-308}
	AGTR1	chr3:148415571-148460795	1.75	<2.23x10^{-308}
	DCBLD1	chr6:117774980-117891021	1.73	<2.23x10^{-308}
	SLC12A2	chr5:127419458-127525380	1.72	<2.23x10^{-308}
	RCAN2	chr6:46188475-46459709	1.68	<2.23x10^{-308}
	GRID1	chr10:87359312-88126250	1.66	<2.23x10^{-308}
	RNF152	chr18:59475296-59561480	1.63	<2.23x10^{-308}
	PDE1C	chr7:31790793-32338941	1.63	<2.23x10^{-308}
	GJC1	chr17:42875816-42908184	1.62	<2.23x10^{-308}
	RGS5	chr1:163080911-163291577	1.61	<2.23x10^{-308}
	SLC7A2	chr8:17354597-17428082	1.59	<2.23x10^{-308}
	NR2F2	chr15:96869167-96883492	1.56	<2.23x10^{-308}
	PARM1	chr4:75858305-75975325	1.53	<2.23x10^{-308}
	PAWR	chr12:79968759-80084877	1.45	<2.23x10^{-308}

HES4	chr1:934342-935552	1.44	$<2.23\times10^{-308}$
TINAGL1	chr1:32042116-32053288	1.40	$<2.23\times10^{-308}$
CYTH3	chr7:6201407-6312275	1.56	7.46×10^{-266}
PDE5A	chr4:120415550-120550146	1.47	9.73×10^{-259}
DAAM2	chr6:39760142-39872648	1.61	4.98×10^{-237}
CHSY3	chr5:129240165-129522327	1.90	5.84×10^{-230}
CCDC102B	chr18:66382446-66722426	1.31	2.69×10^{-215}
SH3RF1	chr4:170015407-170192256	1.53	1.60×10^{-208}
NMD3	chr3:160822484-160971320	1.31	8.17×10^{-201}
SH2D3C	chr9:130500596-130541020	0.816	2.02×10^{-158}
FNBP1L	chr1:93913688-94020218	1.17	1.31×10^{-151}
SPRED2	chr2:65537985-65659771	1.13	5.29×10^{-143}
EEF1DP3	chr13:32420978-32527609	1.10	7.60×10^{-137}
ST5	chr11:8714898-8932498	1.01	8.66×10^{-134}
LAMB1	chr7:107564244-107643700	1.05	2.50×10^{-133}
TFPI	chr2:188328957-188430487	0.953	8.81×10^{-132}
ENAH	chr1:225674537-225840844	0.982	1.31×10^{-123}
FARP1	chr13:98794816-99102027	0.842	3.43×10^{-102}
SNRK	chr3:43328004-43466256	0.959	2.39×10^{-100}
KIAA1324L	chr7:86506222-86689015	0.901	1.70×10^{-99}
LPHN2	chr1:81771845-82458120	0.983	1.10×10^{-92}
PTPN13	chr4:87515468-87736324	0.985	6.66×10^{-90}
MPRIP	chr17:16945859-17120993	0.797	3.92×10^{-75}
7-Sep	chr7:35840542-35944917	0.780	3.03×10^{-74}
SLC38A2	chr12:46751972-46766650	0.797	1.66×10^{-71}
MYL9	chr20:35169887-35178228	0.802	1.15×10^{-68}
RERG	chr12:15260717-15501609	0.939	1.45×10^{-66}
PLSCR4	chr3:145910126-145968966	0.719	4.61×10^{-66}
NID1	chr1:236139130-236228462	0.778	1.18×10^{-56}
C9orf3	chr9:97488983-97849441	0.705	9.40×10^{-54}
RPS21	chr20:60962172-60963576	0.485	4.27×10^{-53}
RBMS2	chr12:56915713-56984745	0.611	2.78×10^{-52}
PELI2	chr14:56584532-56768244	0.548	2.77×10^{-40}
VCL	chr10:75757872-75879918	0.622	1.52×10^{-36}
CLIC4	chr1:25071848-25170815	0.502	2.11×10^{-36}
CAMSAP2	chr1:200708686-200829832	0.469	2.14×10^{-35}
TSPAN14	chr10:82213922-82292879	0.584	4.30×10^{-35}
GOLGA8A	chr15:34671269-34880704	0.519	7.55×10^{-32}
RSU1	chr10:16632610-16859527	0.480	1.40×10^{-31}
PTRF	chr17:40554470-40575535	0.371	6.33×10^{-28}
NCKAP1	chr2:183773843-183903586	0.487	1.84×10^{-24}
PPP1R12A	chr12:80167343-80329240	0.415	8.89×10^{-23}
UBR1	chr15:43235095-43398311	0.412	5.86×10^{-22}
PDS5B	chr13:33160564-33352157	0.298	1.68×10^{-21}
STX12	chr1:28099694-28150963	0.360	2.34×10^{-20}
GAB1	chr4:144257915-144395721	0.403	8.36×10^{-20}

	GFPT1	chr2:69546905-69614382	0.514	$2.07x10^{-18}$
	SHPRH	chr6:146185381-146285559	0.386	$4.84x10^{-18}$
	FOXK1	chr7:4683388-4811074	0.412	$7.42x10^{-18}$
	CTC-228N24.3	chr5:127276118-127418864	0.385	$3.28x10^{-16}$
	CAMTA1	chr1:6845384-7829766	0.265	$5.52x10^{-16}$
	SUPT3H	chr6:44777054-45345690	0.299	$3.37x10^{-15}$
	MBD5	chr2:148778580-149275805	0.338	$5.06x10^{-15}$
	MGMT	chr10:131265448-131566271	0.354	$1.27x10^{-14}$
	RYK	chr3:133784147-133969689	0.310	$8.77x10^{-14}$
	KTN1	chr14:56025790-56168244	0.285	$1.49x10^{-13}$
	LINC00486	chr2:33050510-33151760	0.255	$8.16x10^{-13}$
	AEBP2	chr12:19556979-19873735	0.293	$1.71x10^{-11}$
	UBE2E1	chr3:23847394-23932807	0.284	$8.07x10^{-8}$
Preadipocytes	KCND2	chr7:119913722-120390385	2.59	$<2.23x10^{-308}$
	ABCA10	chr17:67143355-67240987	2.56	$<2.23x10^{-308}$
	CFH	chr1:196621008-196716634	2.05	$<2.23x10^{-308}$
	PRICKLE1	chr12:42852140-42984157	1.91	$<2.23x10^{-308}$
	BOC	chr3:112929850-113006303	1.81	$<2.23x10^{-308}$
	PPL	chr16:4932508-5010742	1.79	$<2.23x10^{-308}$
	PODN	chr1:53527854-53551174	1.65	$<2.23x10^{-308}$
	C3	chr19:6677715-6730573	1.62	$<2.23x10^{-308}$
	ANKS1B	chr12:99120235-100378432	1.20	$7.96x10^{-108}$
	IL16	chr15:81451916-81605104	1.13	$4.77x10^{-96}$
	SPATA6	chr1:48761044-48937845	0.977	$2.52x10^{-74}$
	LUC7L3	chr17:48796905-48833574	0.320	$6.68x10^{-10}$
	LINC-PINT	chr7:130626519-130794935	0.361	$1.16x10^{-3}$
	NFAT5	chr16:69598997-69738569	0.310	$1.33x10^{-3}$

[*] Abbreviation for chromosome.

[†] Average log_2 fold change in assigned cell type cluster compared to other cell type clusters (see Methods).

[‡] Bonferroni adjusted *p*-value.

Table S4: Characteristics of the cell-type marker genes in WHRadjBMI co-expression network, as reported by WGCNA[39] and ranked by network membership.

Gene name	Chr[*]:start-end (hg19)	$\log_2$ fold change[†]	Network membership[‡]	WHRadjBMI correlation[§]	Fasting insulin correlation[§]
ETFA[‖]	chr15:76507696-76603813	0.363	0.898	-0.244	-0.454
ACVR1C[‖]	chr2:158383279-158485517	0.317	0.871	-0.185	-0.402
PCCA[‖]	chr13:100741269-101182686	1.04	0.846	-0.201	-0.356
ACSS3[‖]	chr12:81331594-81650533	1.05	0.840	-0.171	-0.405
ANO6[‖]	chr12:45609770-45834187	0.707	0.833	-0.215	-0.366
VEGFA[‖]	chr6:43737921-43754224	0.594	0.801	-0.155	-0.386
HSDL2[‖]	chr9:115142217-115234690	0.962	0.799	-0.167	-0.309
TWIST1[‖]	chr7:19060614-19157295	0.852	0.784	-0.160	-0.392
PFKFB3[‖]	chr10:6186881-6277495	1.13	0.783	-0.184	-0.390
CRLS1[‖]	chr20:5986736-6020699	0.583	0.774	-0.104	-0.314
GHR[‖]	chr5:42423879-42721979	1.95	0.761	-0.100	-0.364
GLUL[¶]	chr1:182350839-182361341	0.869	0.750	-0.206	-0.371
PRKAR2B[‖]	chr7:106685094-106802256	1.14	0.739	-0.142	-0.372
MPDZ[**]	chr9:13105703-13279589	1.19	0.729	-0.133	-0.296
DAPK2[‖]	chr15:64199235-64364232	1.38	0.728	-0.227	-0.375
BCKDHB[‖]	chr6:80816364-81055987	0.375	0.727	-0.145	-0.256
PHLPP1[††]	chr18:60382672-60647666	1.19	0.724	-0.116	-0.386
MCCC1[‖]	chr3:182733006-182833863	0.437	0.723	-0.135	-0.265
RCL1[‖]	chr9:4792869-4885917	0.576	0.716	-0.163	-0.299
MARC1[‖]	chr1:220960101-220987735	1.15	0.711	-0.224	-0.297
ETFDH[‖]	chr4:159593277-159630775	0.343	0.710	-0.069	-0.286
HADHB[¶]	chr2:26466038-26513336	0.286	0.702	-0.124	-0.252
TRHDE-AS1[‖]	chr12:72647288-72668687	2.08	0.701	-0.135	-0.291
SLC19A3[‖]	chr2:228549926-228582728	1.68	0.698	-0.096	-0.413
AQP7[‖]	chr9:33384765-33402643	2.03	0.683	-0.153	-0.258
WDR20[‖]	chr14:102605840-102691184	0.304	0.672	-0.094	-0.223

CIDEA‖	chr18:12254318-12277594	0.986	0.671	-0.161	-0.392
HADHA¶	chr2:26413504-26467594	0.312	0.669	-0.177	-0.353
SULF1††	chr8:70378859-70573150	1.61	0.657	-0.185	-0.300
EYS‖	chr6:64429876-66417118	0.699	0.656	-0.140	-0.224
SIK2‖	chr11:111473115-111601577	1.64	0.653	-0.038	-0.252
ACO1‖	chr9:32384618-32454767	0.891	0.638	-0.175	-0.250
VWA8‖	chr13:42140973-42535256	0.255	0.635	-0.146	-0.180
KCNIP2‖	chr10:103585731-103603677	1.25	0.631	-0.096	-0.271
NRIP1‖	chr21:16333556-16437321	0.863	0.628	-0.111	-0.287
LRIG1‖	chr3:66429221-66551687	0.880	0.615	-0.117	-0.262
SLC16A7‖	chr12:59989848-60176395	0.657	0.602	-0.185	-0.267
NEDD4L‖	chr18:55711599-56068772	1.25	0.597	-0.170	-0.318
EMC3‖	chr3:10004221-10052800	0.815	0.592	-0.034	-0.304
GFPT1‡‡	chr2:69546905-69614382	0.514	0.579	-0.096	-0.281
ACO2¶	chr22:41865129-41924993	0.272	0.575	-0.113	-0.203
RTN3‖	chr11:63448918-63527363	0.850	0.544	-0.136	-0.225
ADIPOQ‖	chr3:186560463-186576252	1.30	0.538	-0.071	-0.243
NEK6¶	chr9:127019885-127115586	0.728	-0.505	0.097	0.377
CORO1C¶	chr12:109038885-109125372	1.10	-0.538	0.256	0.327
SPARC‖	chr5:151040657-151066726	0.943	-0.541	0.290	0.371
ACTN1¶	chr14:69340860-69446157	0.763	-0.632	0.211	0.379

* Abbreviation for chromosome.

† Average $\log_2$ fold change in adipocytes compared to other cell types (see Methods).

‡ Pearson correlation coefficient with network eigengene as reported by WGCNA[39].

§ Pearson correlation coefficient with phenotype as reported by WGCNA[39].

‖ Adipocyte marker gene from snRNA-seq data[33,43,44] (see Methods).

¶ Macrophage marker gene from snRNA-seq data[33,43,44] (see Methods).

** Endothelial marker gene from snRNA-seq data[33,43,44] (see Methods).

†† Fibroblast marker gene from snRNA-seq data[33,43,44] (see Methods).

‡‡ Perivascular marker gene from snRNA-seq data[33,43,44] (see Methods).

Table S5: KEGG pathway enrichment results (passing FDR<0.05) from WebGestalt[75] for the WHRadjBMI co-expression network genes.

Gene set	Description of the pathway	Enrichment Ratio	*p*-value	FDR
hsa00280	Valine, leucine, and isoleucine degredation	14.91	$<2.2x10^{-16}$	$<2.2x10^{-16}$
hsa00640	Propanoate metabolism	15.43	$3.19x10^{-12}$	$5.17x01^{-10}$
hsa01200	Carbon metabolism	6.15	$1.28x10^{-9}$	$1.38x10^{-7}$
hsa00020	Citrate cycle (TCA cycle)	11.98	$2.55x10^{-8}$	$2.07x10^{-6}$
hsa01100	Metabolic pathways	1.94	$5.58x10^{-8}$	$3.62x10^{-6}$
hsa00630	Glyoxylate and dicoarboxylate metabolism	11.05	$3.23x10^{-7}$	$1.74x10^{-5}$
hsa00071	Fatty acid degredation	7.80	$1.48x10^{-6}$	$6.83x10^{-5}$
hsa01212	Fatty acid metabolism	5.55	$2.23x10^{-4}$	$9.03x10^{-3}$
hsa00650	Butanoate metabolism	7.77	$3.66x10^{-4}$	0.0132
hsa03320	PPAR signaling pathway	4.28	0.00113	0.0366
hsa04932	Non-alcoholic fatty liver disease (NAFLD)	2.87	0.00149	0.0439

Table S6: Gene Ontology cellular component enrichment results (passing FDR<0.05) from WebGestalt[75] for the WHRadjBMI co-expression network genes.

Gene set	Description of the cellular component	Enrichment Ratio	*p*-value	FDR
GO:005739	Mitochondrion	4.40	$<2.2x10^{-16}$	$<2.2x10^{-16}$
GO:0031967	Organelle envelope	3.26	$<2.2x10^{-16}$	$<2.2x10^{-16}$
GO:0031975	Envelope	3.26	$<2.2x10^{-16}$	$<2.2x10^{-16}$
GO:0044429	Mitochondrial part	5.64	$<2.2x10^{-16}$	$<2.2x10^{-16}$
GO:0005740	Mitochondrial envelope	4.57	$<2.2x10^{-16}$	$<2.2x10^{-16}$
GO:0031966	Mitochondrial membrane	4.77	$<2.2x10^{-16}$	$<2.2x10^{-16}$
GO:0019866	Organelle inner membrane	5.26	$<2.2x10^{-16}$	$<2.2x10^{-16}$
GO:0005759	Mitochondrial matrix	8.11	$<2.2x10^{-16}$	$<2.2x10^{-16}$
GO:0005743	Mitochondrial inner membrane	5.59	$<2.2x10^{-16}$	$<2.2x10^{-16}$
GO:0098798	Mitochondrial protein complex	7.32	$<2.2x10^{-16}$	$<2.2x10^{-16}$
GO:1990204	Oxidoreductase complex	9.88	$2.22x10^{-13}$	$2.31x10^{-11}$
GO:0044455	Mitochondrial membrane part	6.03	$1.64x10^{-12}$	$1.56x10^{-10}$
GO:0045239	Tricarboxylic acid cycle enzyme complex	30.16	$8.34x10^{-10}$	$7.34x10^{-8}$
GO:0045240	Dihydrolipoyl dehydrogenase complex	33.61	$6.04x10^{-9}$	$4.94x10^{-7}$
GO:0098800	Inner mitochondrial membrane protein complex	6.09	$2.43x10^{-7}$	$1.86x10^{-6}$
GO:0000313	Organellar ribosome	7.33	$2.78x10^{-7}$	$1.87x10^{-5}$
GO:0005761	Mitochondrial ribosome	7.33	$2.78x10^{-7}$	$1.87x10^{-5}$
GO:0043209	Myelin sheath	5.16	$5.69x10^{-7}$	$3.62x10^{-5}$
GO:0045252	Oxoglutarate dehydrogenase complex	37.34	$1.45x10^{-6}$	$8.72x10^{-5}$
GO0032592	Integral component of mitochondrial membrane	7.52	$2.77x10^{-6}$	$1.59x10^{-4}$
GO:0098573	Intrinsic component of mitochondrial membrane	7.41	$3.15x10^{-6}$	$1.66x10^{-4}$
GO:0030062	Mitochondrial tricarboxylic acid cycle enzyme complex	32.01	$3.33x10^{-6}$	$1.66x10^{-4}$
GO:0045254	Pyruvate dehydrogenase complex	32.01	$3.33x10^{-6}$	$1.66x10^{-4}$
GO:0031304	Intrinsic component of mitochondrial inner membrane	10.32	$4.19x10^{-6}$	$1.92x10^{-4}$

GO:0031305	Integral component of mitochondrial inner membrane	10.32	4.19x10^{-6}	1.92x10^{-4}
GO:0000314	Organellar small ribosomal subunit	12.45	6.60x10^{-6}	2.80x10^{-4}
GO:0005763	Mitochondrial small ribosomal subunit	12.45	6.60x10^{-6}	2.80x10^{-4}
GO:0009295	Nucleoid	9.56	7.13x10^{-6}	2.81x10^{-4}
GO:0042645	Mitochondrial nucleoid	9.56	7.13x10^{-6}	2.81x10^{-4}
GO:0031968	Organelle outer membrane	3.77	4.37x10^{-5}	1.67x10^{-3}
GO:0019867	Outer membrane	3.73	4.86x10^{-5}	1.69x10^{-3}
GO:0005947	Mitochondrial alpha-ketoglutarate dehydrogenase complex	33.61	5.48 x10^{-5}	1.69 x10^{-3}
GO:0005749	Mitochondrial respiratory chain complex II, succinate dehydrogenase complex (ubiquinone)	33.61	5.48 x10^{-5}	1.69 x10^{-3}
GO:0045257	Succinate dehydrogenase complex (ubiquinone)	33.61	5.48 x10^{-5}	1.69 x10^{-3}
GO:0045273	Respiratory chain complex II	33.61	5.48 x10^{-5}	1.69 x10^{-3}
GO:0045281	Succinate dehydrogenase complex	33.61	5.48 x10^{-5}	1.69 x10^{-3}
GO:0045283	Fumarate reductase complex	33.61	5.48 x10^{-5}	1.69 x10^{-3}
GO:0005741	Mitochondrial outer mmbrane	3.89	6.48x10^{-5}	1.95 x10^{-3}
GO:1902494	Catalytic complex	1.83	8.09x10^{-5}	2.37 x10^{-3}
GO:0005777	Peroxisome	4.34	0.00011	2.94 x10^{-3}
GO:0042579	Microbody	4.34	0.00011	2.94 x10^{-3}
GO:0098803	Respiratory chain complex	5.33	0.00013	3.41x10^{-3}
GO:0005840	Ribosome	3.03	0.00065	0.0153
GO:0098796	Membrane protein complex	1.76	0.0015	0.0323

Table S7: Stratified LD Score Regression[55,56] results for WHRadjBMI, T2D, and BMI using the *cis* variants (+/-500kb from the ends of the gene) of the WHRadjBMI co-expression network genes.

Trait	N	Prop SNPs*	Prop $h^{2\dagger}$	Prop h^2 SE‡	Enrichment	Enrichment SE	Enrichment *p*-value
WHRadjBMI – Combined	694,649	0.105	0.169	0.0153	1.61	0.146	4.90×10^{-5}
WHRadjBMI – Male	315,284	0.105	0.154	0.0148	1.46	0.140	1.52×10^{-3}
WHRadjBMI – Female	379,501	0.105	0.178	0.0163	1.69	0.155	2.11×10^{-5}
T2D – Combined	389,738	0.105	0.157	0.0203	1.49	0.193	9.56×10^{-3}
T2D – Male	178,809	0.105	0.161	0.0240	1.53	0.228	0.0177
T2D – Female	210,929	0.105	0.160	0.0304	1.52	0.289	NS
BMI – Combined	806,834	0.105	0.102	0.00560	0.97	0.0532	NS

* Proportion of SNPs in the *cis*-regions (+/-500kb from the ends of the gene) of the WHRadjBMI co-expression network genes.
† Proportion of heritability explained by the variants in the *cis*-regions (+/-500kb from the ends of the gene) of the WHRadjBMI co-expression network genes.
‡ Standard error of the proportion of heritability.

Table S8: Characteristics of the TFs in WHRadjBMI co-expression network, as reported by WGCNA[39] and ranked by network membership.

Gene name	Chr*:start-end (hg19)	Network membership†	WHRadjBMI correlation‡	Fasting insulin correlation‡
HLF	chr17:53342373-53402426	0.794	-0.195	-0.381
TWIST1	chr7:19060614-19157295	0.784	-0.160	-0.392
KLF15	chr3:126061478-126076285	0.775	-0.215	-0.300
PPARA	chr22:46546424-46639653	0.762	-0.160	-0.362
PER3	chr1:7844380-7905237	0.759	-0.186	-0.332
CCNH	chr5:86687311-86708836	0.716	-0.152	-0.304
SIX4	chr14:61176246-61191066	0.712	-0.204	-0.270
TBX15	chr1:119425669-119532179	0.712	-0.204	-0.311
HOMEZ	chr14:23741666-23768656	0.708	-0.272	-0.316
PPARG	chr3:12328867-12475855	0.666	-0.257	-0.308
GTF2E2	chr8:30435835-30515768	0.655	-0.165	-0.292
XPNPEP3	chr22:41253081-41363838	0.655	-0.118	-0.277
ZNF3	chr7:99661656-99680171	0.633	-0.127	-0.296
IRX1	chr5:3596168-3601517	0.574	-0.173	-0.263

* Abbreviation for chromosome.
† Pearson correlation coefficient with network eigengene as reported by WGCNA[39].
‡ Pearson correlation coefficient with phenotype as reported by WGCNA[39].

Table S9: Significant TWAS[58] heritability estimates (*p*<0.01) for the TFs in the WHRadjBMI co-expression network.

Gene Name	Heritability	Heritability standard error	*p*-value
TBX15	0.101	0.0313	$4.07x10^{-6}$
GTF2E2	0.0703	0.0253	$1.43x10^{-3}$
XPNPEP3	0.302	0.0602	$1.18x10^{-38}$
IRX1	0.0813	0.0237	$1.04x10^{-4}$
ZNF3	0.0821	0.0326	$1.85x10^{-5}$

Table S10: TWAS[58] *p*-values and Z-scores for associations of TFs (with significant TWAS[58] heritability (p<0.01)) with WHRadjBMI.

Gene Name	TWAS Model[†]	Z-score	*p*-value
TBX15	Bayesian sparse linear mixed models (bslmm)	15.2	2.11×10^{-52}
GTF2E2	Least absolute shrinkage and selection operator (lasso)	-0.103	NS[*]
XPNPEP3	Least absolute shrinkage and selection operator (lasso)	-3.05	2.26×10^{-3}
IRX1	Best linear unbiased predictor	4.61	4.03×10^{-6}
ZNF3	Least absolute shrinkage and selection operator (lasso)	2.18	NS[*]

[*] NS indicates a non-significant Bonferroni corrected *p*-value>0.017.
[†] Best model for expression imputation chosen by TWAS[58].

Table S11: Significantly differentially expressed genes (FDR<0.05) in the WHRadjBMI co-expression network between the baseline and follow-up time points in the KOBS cohort[30-33] ranked by *p*-value.

Gene name	Chr*:start-end (hg19)	$\log_2$ fold change†	*p*-value	FDR
PHF13	chr1:6673745-6684093	0.343	$4.73x10^{-19}$	$1.70x10^{-16}$
GSDMB‡	chr17:38060848-38076107	0.671	$6.22\ x10^{-18}$	$1.48x10^{-15}$
ZNF3	chr7:99661656-99680171	0.160	$1.64\ x10^{-17}$	$3.34x10^{-15}$
TP73-AS1‡	chr1:3652548-3663900	0.168	$7.21\ x10^{-16}$	$7.37x10^{-14}$
HLF‡	chr17:53342373-53402426	0.631	$1.56\ x10^{-15}$	$1.46x10^{-13}$
CHKA‡	chr11:67820326-67888911	0.287	$2.03\ x10^{-15}$	$1.82x10^{-13}$
ORMDL3‡	chr17:38077294-38083854	0.424	$2.06\ x10^{-14}$	$1.35x10^{-12}$
CDKN1C	chr11:2904443-2907111	0.407	$2.51\ x10^{-14}$	$1.58x10^{-12}$
MLX‡	chr17:40719086-40725257	0.160	$5.77\ x10^{-14}$	$3.17x10^{-12}$
LRRC47	chr1:3696784-3713068	0.134	$1.37\ x10^{-13}$	$6.67x10^{-12}$
NEK6‡	chr9:127019885-127115586	-0.307	$5.82\ x10^{-13}$	$2.21x10^{-11}$
CORO1C‡	chr12:109038885-109125372	-0.257	$3.03\ x10^{-12}$	$9.57x10^{-11}$
CA3‡	chr8:86285665-86361269	1.327	$3.23\ x10^{-12}$	$1.01x10^{-10}$
TM7SF2	chr11:64879317-64883856	0.525	$3.61\ x10^{-12}$	$1.11x10^{-10}$
PMM1	chr22:41972898-41985894	0.285	$4.75\ x10^{-12}$	$1.40x10^{-10}$
SIRT3‡	chr11:215458-236931	0.171	$1.10\ x10^{-11}$	$2.95x10^{-10}$
GNG2‡	chr14:52292913-52446060	-0.365	$1.37\ x10^{-11}$	$3.54x10^{-10}$
EIF4EBP1‡	chr8:37887859-37917883	0.364	$1.51\ x10^{-11}$	$3.86x10^{-10}$
DAPK2‡	chr15:64199235-64364232	0.419	$1.79\ x10^{-11}$	$4.47x10^{-10}$
VPS72	chr1:151142463-151167797	0.125	$2.20\ x10^{-11}$	$5.39x10^{-10}$
LETMD1‡	chr12:51441745-51454207	0.159	$6.80\ x10^{-11}$	$1.45x10^{-9}$
SRSF4‡	chr1:29474255-29508499	0.127	$1.18\ x10^{-10}$	$2.37x10^{-9}$
TTC36	chr11:118398187-118401912	0.930	$1.36\ x10^{-10}$	$2.66x10^{-9}$
PDK2‡	chr17:48172101-48189516	0.287	$1.73\ x10^{-10}$	$3.30x10^{-9}$
MSC‡	chr8:72753784-72756703	-0.541	$2.18\ x10^{-10}$	$4.03x10^{-9}$
MED9‡	chr17:17380300-17396540	0.147	$2.67\ x10^{-10}$	$4.85x10^{-9}$
EIF1	chr17:39845137-39848920	0.139	$3.77\ x10^{-10}$	$6.50x10^{-9}$
SLC43A1	chr11:57252007-57283259	0.252	$5.44\ x10^{-10}$	$8.94x10^{-9}$
ZNF16‡	chr8:146155744-146176274	0.170	$7.30\ x10^{-10}$	$1.16x10^{-8}$
MRPL10‡	chr17:45900638-45908900	0.134	$7.50\ x10^{-10}$	$1.19x10^{-8}$
GLYCTK‡	chr3:52321105-52329272	0.422	$7.61\ x10^{-10}$	$1.20x10^{-8}$
CD248	chr11:66081958-66084515	-0.406	$8.38\ x10^{-10}$	$1.31x10^{-8}$
TMEM104	chr17:72772622-72835918	-0.179	$1.65\ x10^{-9}$	$2.40x10^{-8}$
MRPS9	chr2:105654441-105716418	0.124	$2.80\ x10^{-9}$	$3.88x10^{-8}$
PHLPP1	chr18:60382672-60647666	0.246	$3.09\ x10^{-9}$	$4.24x10^{-8}$
ZFYVE21	chr14:104182067-104200005	0.115	$4.50\ x10^{-9}$	$5.86x10^{-8}$
SLC27A2‡	chr15:50474393-50528592	1.114	$7.33\ x10^{-9}$	$9.01x10^{-8}$
GPR146‡	chr7:1084212-1098897	0.270	$7.82\ x10^{-9}$	$9.53x10^{-8}$
DLST	chr14:75348594-75370448	0.109	$8.96\ x10^{-9}$	$1.07x10^{-7}$
ANKRD53‡	chr2:71205510-71212626	0.380	$8.98\ x10^{-9}$	$1.08x10^{-7}$

ADCK3[‡]	chr1:227085237-227175246	0.310	9.59 x10^{-9}	1.14x10^{-7}
AASS	chr7:121715701-121784334	0.276	1.86 x10^{-8}	2.05x10^{-7}
RP11-61I13.3[‡]	chr6:39849580-39867847	0.459	2.06 x10^{-8}	2.24x10^{-7}
NDRG4[‡]	chr16:58496750-58547532	0.594	2.49 x10^{-8}	2.63x10^{-7}
PER3[‡]	chr1:7844380-7905237	0.361	7.41 x10^{-8}	6.87x10^{-7}
ANG[‡]	chr14:21152336-21167130	0.236	9.36 x10^{-8}	8.43x10^{-7}
TMEM52[‡]	chr1:1849029-1850712	0.409	1.14 x10^{-7}	1.01x10^{-6}
HPD[‡]	chr12:122277433-122301502	-0.432	1.15 x10^{-7}	1.01x10^{-6}
TMEM25[‡]	chr11:118401756-118417995	0.269	1.30 x10^{-7}	1.13x10^{-6}
THYN1	chr11:134118173-134123264	0.146	1.40 x10^{-7}	1.20x10^{-6}
TMEM132C[‡]	chr12:128751948-129192460	0.320	1.66 x10^{-7}	1.40x10^{-6}
RPAIN	chr17:5322961-5336196	0.136	2.54 x10^{-7}	2.05x10^{-6}
SLC35G2[‡]	chr3:136537489-136574734	0.229	2.67 x10^{-7}	2.13x10^{-6}
PRKAG2-AS1	chr7:151574127-151576299	0.243	2.94 x10^{-7}	2.32x10^{-6}
C1orf50[‡]	chr1:43232940-43263968	0.119	3.05 x10^{-7}	2.40x10^{-6}
DSEL	chr18:65173819-65184217	-0.236	3.84 x10^{-7}	2.94x10^{-6}
RP11-387H17.4[‡]	chr17:38083995-38095854	1.021	4.00 x10^{-7}	3.04x10^{-6}
KRT5[‡]	chr12:52908359-52914471	-0.701	6.04 x10^{-7}	4.39x10^{-6}
PCBD1[‡]	chr10:72642037-72648541	0.161	6.69 x10^{-7}	4.80x10^{-6}
GPR180	chr13:95254157-95286899	-0.238	1.01 x10^{-6}	6.85x10^{-6}
SLC41A1	chr1:205758221-205782876	0.162	1.22 x10^{-6}	8.14x10^{-6}
NMNAT3[‡]	chr3:139279022-139396859	0.186	1.28 x10^{-6}	8.49x10^{-6}
TBX15	chr1:119425669-119532179	0.367	1.48 x10^{-6}	9.59x10^{-6}
NIPSNAP3B	chr9:107526438-107539738	0.258	1.74 x10^{-6}	1.11x10^{-5}
MOCS1[‡]	chr6:39867354-39902290	0.225	2.18 x10^{-6}	1.36x10^{-5}
RASL10B	chr17:34058668-34070540	0.658	2.53 x10^{-6}	1.55x10^{-5}
VEGFA[‡]	chr6:43737921-43754224	0.208	3.62 x10^{-6}	2.13x10^{-5}
IMMT[‡]	chr2:86371055-86422893	-0.091	3.80 x10^{-6}	2.23x10^{-5}
FHOD3	chr18:33877677-34360018	0.428	7.09 x10^{-6}	3.87x10^{-5}
URAHP[‡]	chr16:90106169-90114181	0.259	1.00 x10^{-5}	5.26x10^{-5}
LBP	chr20:36974759-37005665	-0.608	1.02 x10^{-5}	5.32x10^{-5}
RCL1[‡]	chr9:4792869-4885917	0.178	1.36 x10^{-5}	6.80x10^{-5}
AKAP1	chr17:55162453-55198710	0.171	1.63 x10^{-5}	7.99x10^{-5}
SLC25A21-AS1	chr14:37641093-37643016	0.284	1.68 x10^{-5}	8.19x10^{-5}
STOX1	chr10:70587298-70655188	0.454	1.70 x10^{-5}	8.29x10^{-5}
ADH1A	chr4:100197524-100212185	0.464	2.21 x10^{-5}	1.04x10^{-4}
BFAR[‡]	chr16:14726672-14763093	0.075	2.30 x10^{-5}	1.08x10^{-4}
AP5S1	chr20:3801178-3805949	-0.122	2.45 x10^{-5}	1.14x10^{-4}
IFT46[‡]	chr11:118415243-118443685	0.107	2.65 x10^{-5}	1.22x10^{-4}
C17orf53	chr17:42219274-42239844	0.247	2.73 x10^{-5}	1.25x10^{-4}
PRDX3	chr10:120927215-120938345	-0.161	2.89 x10^{-5}	1.31x10^{-4}
ACSS3	chr12:81331594-81650533	0.237	2.94 x10^{-5}	1.33x10^{-4}
ST6GALNAC6[‡]	chr9:130647600-130667687	0.117	3.17 x10^{-5}	1.43x10^{-4}
RP11-689P11.2[‡]	chr4:8483997-8514337	0.287	3.77 x10^{-5}	1.66x10^{-4}
SNX3	chr6:108532426-108582464	-0.115	4.08 x10^{-5}	1.78x10^{-4}

MRS2‡	chr6:24403153-24425810	-0.123	4.25 x10^{-5}	1.84x10^{-4}
SRP68‡	chr17:74035184-74068734	0.064	5.00 x10^{-5}	2.13x10^{-4}
ELP2‡	chr18:33709407-33757909	0.068	5.17 x10^{-5}	2.19x10^{-4}
STXBP1‡	chr9:130374544-130457460	0.117	5.31 x10^{-5}	2.25x10^{-4}
HADHB‡	chr2:26466038-26513336	-0.089	5.61 x10^{-5}	2.35x10^{-4}
GINS3‡	chr16:58328984-58440048	0.183	6.29 x10^{-5}	2.60x10^{-4}
UNG	chr12:109535379-109548797	0.124	6.32 x10^{-5}	2.61x10^{-4}
USP13	chr3:179370543-179507189	0.150	8.53 x10^{-5}	3.40x10^{-4}
DNAJC19‡	chr3:180701497-180707562	0.085	8.94 x10^{-5}	3.55x10^{-4}
HADH‡	chr4:108910870-108956331	0.191	1.10 x10^{-4}	4.26x10^{-4}
PDP2‡	chr16:66912492-66929657	-0.196	1.25 x10^{-4}	4.78x10^{-4}
UQCRC2‡	chr16:21963981-21994981	0.081	1.32 x10^{-4}	5.00x10^{-4}
GHITM‡	chr10:85899196-85913001	-0.090	1.34 x10^{-4}	5.07x10^{-4}
APBB1IP‡	chr10:26727132-26856732	-0.185	1.46 x10^{-4}	5.47x10^{-4}
LONRF1‡	chr8:12579403-12613582	0.241	1.69 x10^{-4}	6.24x10^{-4}
GLIS1	chr1:53971910-54199877	0.352	1.87 x10^{-4}	6.81x10^{-4}
CENPV‡	chr17:16245848-16256970	0.160	1.88 x10^{-4}	6.85x10^{-4}
SPARC	chr5:151040657-151066726	-0.218	1.96 x10^{-4}	7.10x10^{-4}
NEDD4L‡	chr18:55711599-56068772	0.200	2.01 x10^{-4}	7.26x10^{-4}
CSNK2A2‡	chr16:58191811-58231824	0.068	2.12 x10^{-4}	7.60x10^{-4}
KLF15‡	chr3:126061478-126076285	0.253	2.64 x10^{-4}	9.21x10^{-4}
GYG2P1	chrY:14475147-14532255	0.428	3.05 x10^{-4}	1.04x10^{-3}
UTS2B‡	chr3:190984957-191048325	0.347	3.12 x10^{-4}	1.07x10^{-3}
ANKRD4‡6	chr8:101521980-101572012	-0.140	3.14 x10^{-4}	1.07x10^{-3}
RP11-474O21.5	chr1:12678906-12679250	0.236	3.81 x10^{-4}	1.27x10^{-3}
TUBB2A‡	chr6:3153903-3157760	-0.259	4.77 x10^{-4}	1.55x10^{-3}
ISCA1	chr9:88879461-88897676	-0.088	5.56 x10^{-4}	1.77x10^{-3}
FBXL5	chr4:15606162-15683302	-0.119	5.61 x10^{-4}	1.78x10^{-3}
RAI2	chrX:17818169-17879457	0.182	5.82 x10^{-4}	1.84x10^{-3}
MKNK2‡	chr19:2037470-2051243	0.110	7.35 x10^{-4}	2.27x10^{-3}
SCOC‡	chr4:141178440-141306880	-0.142	8.51 x10^{-4}	2.57x10^{-3}
GFPT1‡	chr2:69546905-69614382	-0.142	8.58 x10^{-4}	2.59x10^{-3}
MCCC1‡	chr3:182733006-182833863	0.140	9.11 x10^{-4}	2.73x10^{-3}
MRPS35	chr12:27863706-27909228	-0.073	9.26 x10^{-4}	2.77x10^{-3}
GLUL‡	chr1:182350839-182361341	0.222	9.54 x10^{-4}	2.85x10^{-3}
GPATCH11	chr2:37311594-37326387	-0.143	9.93 x10^{-4}	2.95x10^{-3}
PDHX‡	chr11:34937376-35042138	-0.126	1.02 x10^{-3}	3.03x10^{-3}
HSDL2‡	chr9:115142217-115234690	-0.127	1.05 x10^{-3}	3.10x10^{-3}
IARS2‡	chr1:220267444-220321380	-0.080	1.08 x10^{-3}	3.19x10^{-3}
GPD1L	chr3:32147181-32210205	0.164	1.10 x10^{-3}	3.22x10^{-3}
TMEM220‡	chr17:10602332-10633633	0.092	1.23 x10^{-3}	3.56x10^{-3}
TARSL2	chr15:102193801-102264807	0.093	1.24 x10^{-3}	3.59x10^{-3}
MRPS18A	chr6:43639040-43655528	0.073	1.26 x10^{-3}	3.64x10^{-3}
AK4‡	chr1:65613232-65697828	-0.150	1.28 x10^{-3}	3.70x10^{-3}
ADAMTS9-AS2	chr3:64670585-64997143	0.174	1.42 x10^{-3}	4.05x10^{-3}

CPT2	chr1:53662101-53679869	-0.096	1.43 $x10^{-3}$	$4.06x10^{-3}$
TXLNG‡	chrX:16804550-16862642	-0.112	1.45 $x10^{-3}$	$4.12x10^{-3}$
LRRC41‡	chr1:46726868-46769280	0.053	1.54 $x10^{-3}$	$4.34x10^{-3}$
PPARA‡	chr22:46546424-46639653	0.098	1.54 $x10^{-3}$	$4.34x10^{-3}$
DNAJA3	chr16:4475806-4506776	0.064	1.65 $x10^{-3}$	$4.61x10^{-3}$
ACAT1‡	chr11:107992243-108018503	0.100	1.99 $x10^{-3}$	$5.43x10^{-3}$
PAIP2B	chr2:71409869-71454213	0.140	2.07 $x10^{-3}$	$5.62x10^{-3}$
RBPMS-AS1	chr8:30239635-30242917	0.233	2.20 $x10^{-3}$	$5.93x10^{-3}$
UQCC1	chr20:33890369-33999944	0.055	2.33 $x10^{-3}$	$6.22x10^{-3}$
ARPC1A‡	chr7:98923521-98985787	0.054	2.38 $x10^{-3}$	$6.36x10^{-3}$
DHTKD1‡	chr10:12110971-12165224	0.093	2.40 $x10^{-3}$	$6.40x10^{-3}$
MRPL32	chr7:42971799-42988557	0.074	2.87 $x10^{-3}$	$7.48x10^{-3}$
TRHDE-AS1	chr12:72647288-72668687	-0.184	3.85 $x10^{-3}$	$9.70x10^{-3}$
MARC2‡	chr1:220921567-220958150	0.087	3.95 $x10^{-3}$	$9.90x10^{-3}$
GPHN‡	chr14:66974125-67648520	0.101	3.96 $x10^{-3}$	$9.93x10^{-3}$
PMPCB	chr7:102937869-102969958	0.062	4.28 $x10^{-3}$	0.0106
APMAP‡	chr20:24943561-24973615	0.093	4.58 $x10^{-3}$	0.0113
BTG3	chr21:18965971-18985265	0.095	4.77 $x10^{-3}$	0.0117
TOMM70A‡	chr3:100082275-100120242	-0.079	5.13 $x10^{-3}$	0.0124
ATP5F1‡	chr1:111991486-112005395	-0.058	5.70 $x10^{-3}$	0.0136
NDUFA5‡	chr7:123177051-123198309	-0.097	6.10 $x10^{-3}$	0.0144
TMEM230	chr20:5080486-5093749	-0.073	6.37 $x10^{-3}$	0.0150
HDDC2	chr6:125541108-125623282	0.066	7.95 $x10^{-3}$	0.0182
MPC1‡	chr6:166778407-166796486	0.076	8.33 $x10^{-3}$	0.0190
SLC25A27‡	chr6:46620678-46645930	0.107	8.34 $x10^{-3}$	0.0190
TCEB3	chr1:24069645-24088549	-0.060	8.40 $x10^{-3}$	0.0191
NDFIP2‡	chr13:80055287-80130210	-0.104	8.56 $x10^{-3}$	0.0194
HOMEZ‡	chr14:23741666-23768656	0.082	8.75 $x10^{-3}$	0.0198
TMLHE	chrX:154719776-154899605	0.091	8.78 $x10^{-3}$	0.0198
PTPN3‡	chr9:112137746-112260590	0.142	9.01 $x10^{-3}$	0.0203
RDH10	chr8:74206847-74237516	-0.131	9.24 $x10^{-3}$	0.0208
DPP3‡	chr11:66247484-66277130	-0.081	9.74 $x10^{-3}$	0.0217
KTN1-AS1‡	chr14:55965996-56046828	0.112	0.0106	0.0234
DLD‡	chr7:107531415-107572175	-0.094	0.0110	0.0242
RP11-61A14.2	chr16:66921918-66922834	-0.150	0.0118	0.0257
SDHC	chr1:161284047-161332984	-0.064	0.0119	0.0259
SYAP1	chrX:16737755-16783459	-0.059	0.0120	0.0260
ADH1B	chr4:100226121-100242558	0.167	0.0126	0.0272
TUSC1	chr9:25676396-25678856	0.075	0.0132	0.0283
ALDH6A1	chr14:74523553-74551196	0.107	0.0164	0.0341
FAM13A‡	chr4:89647106-90032549	0.128	0.0167	0.0347
MRPL45	chr17:36452989-36479101	0.043	0.01684	0.0349
HSPA9‡	chr5:137890571-137911133	-0.058	0.01710	0.0354
PAXIP1-AS1	chr7:154795158-154797413	0.085	0.0181	0.0371
ABHD15	chr17:27887565-27894155	0.077	0.0182	0.0373

ADRBK2[‡]	chr22:25960816-26125261	0.127	0.0190	0.0387
MCCC2[‡]	chr5:70883115-70954531	0.064	0.0191	0.0388
TBC1D20	chr20:416124-443197	0.039	0.0191	0.0390
RGS3[‡]	chr9:116207011-116360018	0.190	0.0193	0.0392
NFU1[‡]	chr2:69622882-69664760	0.072	0.0195	0.0397
ACAD8[‡]	chr11:134123389-134135749	0.047	0.0196	0.0397
DNAH9[‡]	chr17:11501748-11873065	0.167	0.0197	0.0399
ETFA[‡]	chr15:76507696-76603813	0.078	0.0204	0.0412
VDAC2	chr10:76969912-76991206	0.053	0.0236	0.0467

[*] Abbreviation for chromosome.

[†] Average $\log_2$ fold change in expression between baseline and follow-up time points in the KOBS cohort[31-33] (see Methods).

[‡] Gene promoter (+2kb/-1kb from the TSS) contains a TBX15 motif (see Methods).

Table S12: Significantly differentially expressed genes (FDR<0.05) in the WHRadjBMI co-expression network in the *TBX15* knockdown experiment ranked by *p*-value.

Gene name	Chr*:start-end (hg19)	log2 fold change†	*p*-value	FDR
TBX15	chr1:119425669-119532179	-1.527	$1.13x10^{-18}$	$3.35x10^{-16}$
STRADB‡	chr2:202252581-202345569	0.698	$1.81x10^{-13}$	$2.69x10^{-11}$
EIF4EBP2	chr10:72164135-72188374	-0.454	$1.02x10^{-11}$	$1.01x10^{-9}$
MET‡	chr7:116312444-116438440	0.478	$3.69x10^{-11}$	$2.74x10^{-9}$
DSEL	chr18:65173819-65184217	-0.539	$7.78x10^{-11}$	$4.62x10^{-9}$
VEGFA‡	chr6:43737921-43754224	-0.299	$3.09x10^{-11}$	$1.53x10^{-6}$
STXBP1‡	chr9:130374544-130457460	-0.315	$1.98x10^{-7}$	$8.42x10^{-6}$
PHGDH‡	chr1:120202421-120286838	0.266	$5.61x10^{-7}$	$1.92 x10^{-5}$
ISCA1	chr9:88879461-88897676	0.302	$5.82x10^{-7}$	$1.92 x10^{-5}$
SNRNP27‡	chr2:70120692-70132707	-0.438	$1.01x10^{-6}$	$2.95 x10^{-5}$
IMMP2L	chr7:110303110-111202573	-0.418	$1.09x10^{-6}$	$2.95 x10^{-5}$
TMEM189‡	chr20:48697663-48770335	0.312	$1.25x10^{-6}$	$3.09x10^{-5}$
THYN1	chr11:134118173-134123264	-0.280	$2.01x10^{-6}$	$4.59x10^{-5}$
CFL2	chr14:35179593-35184029	0.271	$2.48x10^{-6}$	$5.26x10^{-5}$
UQCRC2‡	chr16:21963981-21994981	-0.184	$3.94x10^{-6}$	$7.79x10^{-5}$
PPARA‡	chr22:46546424-46639653	0.297	$5.24x10^{-6}$	$9.72x10^{-5}$
PCBD1‡	chr10:72642037-72648541	-0.239	$7.22x10^{-6}$	$1.26 x10^{-4}$
FLNA‡	chrX:153576892-153603006	0.249	$8.17x10^{-6}$	$1.35x10^{-4}$
CYB5A‡	chr18:71920530-71959251	-0.207	$1.32x10^{-5}$	$2.07x10^{-4}$
LONRF1‡	chr8:12579403-12613582	-0.281	$1.41x10^{-5}$	$2.09x10^{-4}$
GNG2‡	chr14:52292913-52446060	-0.207	$1.55x10^{-5}$	$2.19x10^{-4}$
APMAP‡	chr20:24943561-24973615	0.182	$1.75x10^{-5}$	$2.30x10^{-4}$
RAI2	chrX:17818169-17879457	0.426	$1.78x10^{-5}$	$2.30x10^{-4}$
KLF15‡	chr3:126061478-126076285	0.459	$2.18x10^{-5}$	$2.69x10^{-4}$
ABHD15	chr17:27887565-27894155	0.285	$2.68x10^{-5}$	$3.19x10^{-4}$
TXLNG‡	chrX:16804550-16862642	-0.191	$3.54x10^{-5}$	$4.04x10^{-4}$
BCKDHB‡	chr6:80816364-81055987	-0.264	$4.63x10^{-5}$	$4.78x10^{-4}$
HMGN3	chr6:79910962-79944406	-0.216	$4.67x10^{-5}$	$4.78x10^{-4}$
ACADSB‡	chr10:124768495-124817827	-0.210	$4.66x10^{-5}$	$4.78x10^{-4}$
TMEM42‡	chr3:44903361-44907162	0.266	$5.81x10^{-5}$	$5.56x10^{-4}$
MAN2A2‡	chr15:91445448-91465814	0.233	$5.70x10^{-5}$	$5.56x10^{-4}$
OSBPL1A‡	chr18:21742008-21977844	-0.155	$6.51x10^{-5}$	$6.04x10^{-4}$
SUCLG2	chr3:67410884-67705038	0.168	$7.22x10^{-5}$	$6.50x10^{-4}$
APBB1IP‡	chr10:26727132-26856732	-0.197	$1.10x10^{-4}$	$9.59x10^{-4}$
MKNK2‡	chr19:2037470-2051243	-0.158	$1.13x10^{-4}$	$9.62x10^{-4}$
AUH‡	chr9:93976097-94124195	-0.255	$1.47x10^{-4}$	$1.18x10^{-3}$
SRP68‡	chr17:74035184-74068734	0.148	$1.43x10^{-4}$	$1.18x10^{-3}$
GRPEL1	chr4:7060633-7069924	-0.173	$1.83x10^{-4}$	$1.43x10^{-3}$
IFT46‡	chr11:118415243-118443685	-0.193	$1.87x10^{-4}$	$1.43x10^{-3}$

GPD1L	chr3:32147181-32210205	0.173	$2.38x10^{-4}$	$1.77x10^{-3}$
TWIST1‡	chr7:19060614-19157295	-0.171	$3.00x10^{-4}$	$2.12x10^{-3}$
DHTKD1‡	chr10:12110971-12165224	0.207	$2.98x10^{-4}$	$2.12x10^{-3}$
PMPCB	chr7:102937869-102969958	-0.152	$3.21x10^{-4}$	$2.16x10^{-3}$
TMEM104	chr17:72772622-72835918	0.163	$3.20x10^{-4}$	$2.16x10^{-3}$
CTH	chr1:70876901-70905534	0.219	$3.30x10^{-4}$	$2.17x10^{-3}$
BTG3	chr21:18965971-18985265	-0.195	$3.50x10^{-4}$	$2.26x10^{-3}$
PFKFB3	chr10:6186881-6277495	0.184	$3.72x10^{-4}$	$2.27x10^{-3}$
XPNPEP3‡	chr22:41253081-41363838	-0.151	$3.61x10^{-4}$	$2.27x10^{-3}$
GTF2E2‡	chr8:30435835-30515768	-0.142	$3.74x10^{-4}$	$2.27x10^{-3}$
LRRC41‡	chr1:46726868-46769280	0.127	$3.82x10^{-4}$	$2.27x10^{-3}$
DPP3‡	chr11:66247484-66277130	-0.163	$4.19x10^{-4}$	$2.44x10^{-3}$
TARSL2	chr15:102193801-102264807	-0.195	$4.50x10^{-4}$	$2.57x10^{-3}$
ATPAF1‡	chr1:47098409-47139539	-0.188	$4.90x10^{-4}$	$2.74x10^{-3}$
CCNH‡	chr5:86687311-86708836	-0.179	$5.28x10^{-4}$	$2.90x10^{-3}$
FAM89A‡	chr1:231154704-231175992	0.305	$5.53x10^{-4}$	$2.99x10^{-3}$
TMEM230	chr20:5080486-5093749	-0.120	$5.80x10^{-4}$	$3.07x10^{-3}$
VPS72	chr1:151142463-151167797	-0.161	$7.24x10^{-4}$	$3.77x10^{-3}$
PPP2R5A	chr1:212458879-212535200	0.167	$8.03x10^{-4}$	$4.04x10^{-3}$
MRS2‡	chr6:24403153-24425810	-0.156	$8.03x10^{-4}$	$4.04x10^{-3}$
PER3‡	chr1:7844380-7905237	-0.208	$8.65x10^{-4}$	$4.28x10^{-3}$
PPARG	chr3:12328867-12475855	-0.140	$8.86x10^{-4}$	$4.31x10^{-3}$
PRAF2‡	chrX:48928813-48931730	-0.231	$9.20x10^{-4}$	$4.41x10^{-3}$
SRSF4‡	chr1:29474255-29508499	-0.159	$9.98x10^{-4}$	$4.67x10^{-3}$
SLC41A1	chr1:205758221-205782876	-0.155	$1.01x10^{-3}$	$4.67x10^{-3}$
DAPK2‡	chr15:64199235-64364232	0.445	$1.09x10^{-3}$	$4.96x10^{-3}$
ISOC1	chr5:128430444-128449721	-0.171	$1.26x10^{-3}$	$5.65x10^{-3}$
GPATCH11	chr2:37311594-37326387	-0.163	$1.28 x10^{-3}$	$5.67x10^{-3}$
NDUFB5‡	chr3:179322478-179345435	-0.123	$1.39 x10^{-3}$	$6.06x10^{-3}$
HIBADH	chr7:27565061-27702614	-0.117	$1.46 x10^{-3}$	$6.20x10^{-3}$
MRPL32	chr7:42971799-42988557	-0.165	$1.44 x10^{-3}$	$6.20x10^{-3}$
PHLPP1	chr18:60382672-60647666	0.177	$1.52 x10^{-3}$	$6.35x10^{-3}$
ANKRD53‡	chr2:71205510-71212626	0.426	$1.60 x10^{-3}$	$6.62x10^{-3}$
MTHFD1‡	chr14:64854749-64926722	0.112	$1.69 x10^{-3}$	$6.68x10^{-3}$
SDHC	chr1:161284047-161332984	0.195	$1.65 x10^{-3}$	$6.68x10^{-3}$
HSPD1‡	chr2:198351305-198381461	-0.111	$1.67 x10^{-3}$	$6.68x10^{-3}$
L2HGDH	chr14:50704281-50779266	-0.211	$1.80 x10^{-3}$	$6.93x10^{-3}$
CHCHD3	chr7:132469629-132766848	-0.147	$1.78 x10^{-3}$	$6.93x10^{-3}$
IARS2‡	chr1:220267444-220321380	0.123	$1.94 x10^{-3}$	$7.39x10^{-3}$
HOMER3‡	chr19:19040010-19052070	-0.139	$2.12 x10^{-3}$	$7.97x10^{-3}$
MLX‡	chr17:40719086-40725257	-0.116	$2.16 x10^{-3}$	$8.03x10^{-3}$
TMEM100‡	chr17:53796988-53809482	0.243	$2.64 x10^{-3}$	$9.67x10^{-3}$

EIF4EBP1‡	chr8:37887859-37917883	0.134	2.94×10^{-3}	0.0106
ABHD5‡	chr3:43731605-43775863	-0.139	3.06×10^{-3}	0.0109
C1orf43‡	chr1:154179182-154193104	-0.103	3.09×10^{-3}	0.0109
ZNF16‡	chr8:146155744-146176274	0.248	3.12×10^{-3}	0.0109
DLD‡	chr7:107531415-107572175	-0.109	3.19×10^{-3}	0.0110
ORMDL3‡	chr17:38077294-38083854	0.134	3.54×10^{-3}	0.0121
CAPN1‡	chr11:64948037-64979477	0.117	3.63×10^{-3}	0.0123
VWA8‡	chr13:42140973-42535256	-0.124	4.12×10^{-3}	0.0135
GLUL‡	chr1:182350839-182361341	0.0897	4.11×10^{-3}	0.0135
GBAS‡	chr7:56019486-56067874	-0.131	4.06×10^{-3}	0.0135
RP11-61A14.3‡	chr16:66923072-66924996	-0.181	4.25×10^{-3}	0.0137
ACTN1‡	chr14:69340860-69446157	-0.102	4.52×10^{-3}	0.0144
ARPC1A‡	chr7:98923521-98985787	0.0967	4.99×10^{-3}	0.0158
MRPS9	chr2:105654441-105716418	-0.135	5.28×10^{-3}	0.0163
RASL10B	chr17:34058668-34070540	0.743	5.25×10^{-3}	0.0163
MRPL39	chr21:26957968-26979829	-0.119	5.84×10^{-3}	0.0177
MAP3K5	chr6:136878185-137113656	0.157	5.82×10^{-3}	0.0177
LRPPRC	chr2:44113647-44223144	-0.0919	6.09×10^{-3}	0.0183
PDHX‡	chr11:34937376-35042138	-0.121	6.41×10^{-3}	0.0190
HADH‡	chr4:108910870-108956331	0.105	6.71×10^{-3}	0.0197
NUDT1‡	chr7:2281857-2290781	0.156	7.03×10^{-3}	0.0205
MOCS1‡	chr6:39867354-39902290	0.172	7.26×10^{-3}	0.0209
PHF13	chr1:6673745-6684093	0.149	7.36×10^{-3}	0.0210
BFAR‡	chr16:14726672-14763093	0.105	7.67×10^{-3}	0.0217
NKIRAS1‡	chr3:23933151-23988082	-0.134	7.76×10^{-3}	0.0217
HADHB‡	chr2:26466038-26513336	0.0999	7.96×10^{-3}	0.0221
EMC3‡	chr3:10004221-10052800	-0.110	8.25×10^{-3}	0.0227
PDHB	chr3:58413357-58419584	-0.0998	8.90×10^{-3}	0.0242
PRDX6	chr1:173446405-173457946	0.0861	9.07×10^{-3}	0.0245
CHKA‡	chr11:67820326-67888911	0.139	0.0100	0.0268
CORO1C‡	chr12:109038885-109125372	0.0825	0.0108	0.0287
CENPV‡	chr17:16245848-16256970	0.126	0.0112	0.0294
LONP2‡	chr16:48278207-48397033	-0.0848	0.0120	0.0303
GRSF1‡	chr4:71681499-71705662	0.0857	0.0118	0.0303
SLC25A27‡	chr6:46620678-46645930	-0.194	0.0119	0.0303
NRIP1	chr21:16333556-16437321	0.126	0.0119	0.0303
GMCL1	chr2:70056774-70108528	0.150	0.0122	0.0307
SCO1	chr17:10583654-10601692	0.0878	0.0129	0.0323
SULF1‡	chr8:70378859-70573150	-0.149	0.0130	0.0323
CD248	chr11:66081958-66084515	-0.129	0.0141	0.0345
C9orf16‡	chr9:130922539-130926207	-0.181	0.0143	0.0348
ANKRD46‡	chr8:101521980-101572012	0.225	0.0152	0.0368

LSM6	chr4:147096837-147121152	-0.135	0.0161	0.0386
CCDC50	chr3:191046866-191116459	0.0816	0.0175	0.0415
GGCT	chr7:30536237-30591095	-0.107	0.0180	0.0424
MRPS27‡	chr5:71515236-71616473	-0.0739	0.0191	0.0446
C11orf1‡	chr11:111749659-111756699	-0.138	0.0208	0.0483
PRDX3	chr10:120927215-120938345	0.109	0.0218	0.0499
ADH1B	chr4:100226121-100242558	0.351	0.0218	0.0499

* Abbreviation for chromosome.

† Average $\log_2$ fold change in expression in human primary preadipocytes transfected with the *TBX15* siRNA when compared to the cells transfected with the negative control siRNA (see Methods).

‡ Gene promoter (+2kb/-1kb from the TSS) contains a TBX15 motif (see Methods).
